# *Salmonella* uses sulfate reductases with unique catalytic activity to promote gut colonization in mice

**DOI:** 10.1038/s41564-026-02443-y

**Published:** 2026-08-05

**Authors:** Ju-Sim Kim, Siva Uppalapati, Alyssa Margolis, Michael McClelland, Hanan Elajaili, Eva Nozik, Lin Liu, Andres Vazquez-Torres

**Affiliations:** 1 Department of Immunology and Microbiology, University of Colorado School of Medicine, Aurora, CO USA; 2 Department of Microbiology and Molecular Genetics, University of California, Irvine, School of Medicine, Irvine, CA USA; 3 Cardiovascular Pulmonary Research Laboratories and Pediatric Critical Care Medicine, University of Colorado Anschutz Medical Campus, Aurora, CO USA; 4 Veterans Affairs, Eastern Colorado Health Care System, Aurora, CO USA

**Keywords:** Bacterial pathogenesis, Pathogens

## Abstract

Non-typhoidal *Salmonella* use molybdenum cofactor-containing MopB- or DMSO reductase-family members to respire chemically diverse substrates, including formate, nitrate and methionine sulfoxide, during infection. The DmsABC enzymatic complex encodes one such DMSO reductase to promote oxidative stress resistance. The *Salmonella* genome encodes several gene paralogues but their role in virulence is unclear. Here we characterize three *Salmonella* MopB-family extracytoplasmic sulfate reductases, which we call Xsr1A, Xsr2A and Xsr3A. Infection experiments in mice and macrophages show that these sulfate reductases support *Salmonella* growth and virulence in the gut and during systemic infection, countering the oxidative effects of host respiratory burst activity. Further experiments show that they are molybdenum cofactor-independent enzymes, and instead depend on the nearby redox-active [4Fe–4S] prosthetic group for catalytic activity. Orthologues of these sulfate reductases were found across distant evolutionary branches, suggesting that [4Fe–4S]-dependent catalysis may occur across the ubiquitous MopB superfamily. Our findings offer insights into the modular evolution of redox centres in the widespread MopB superfamily.

## Abstract (web summary)

Extracytoplasmic MopB-family sulfate reductases using Fe–S clusters instead of molybdenum as a cofactor enable sulfate assimilation in *Salmonella* to support gut colonization and virulence, and help to counter host-imposed oxidative stress.

## Main

Bacterial pathogens balance metabolism and energy output in response to the terminal electron acceptors available in the host. Molecular oxygen (O_2_) is the most energetically favourable, and thus the preferred, terminal electron acceptor for facultative anaerobic bacteria such as non-typhoidal *Salmonella enterica* serovar Typhimurium^1^, a common cause of gastroenteritis in immunocompetent hosts and extraintestinal disease in immunocompromised people. However, *Salmonella* experiences O_2_ shortages in the host. Aerobic respiration by enterocytes lining the intestine dramatically curtails the concentration of O_2_ diffusing into the gut lumen^2^. Consumption of O_2_ in the respiratory burst of the phagocyte NADPH oxidase also induces hypoxia in the *Salmonella*-containing vacuole of macrophages^3,4^. With restricted O_2_ availability, non-typhoidal *Salmonella* must exploit thermodynamically less favourable terminal electron acceptors such as nitrate, tetrathionate or methionine sulfoxide^5,6^. Tetrathionate, nitrate and methionine sulfoxide reductases belong to the ancient dimethyl sulfoxide (DMSO) reductase superfamily (that is, MopB), whose members were already present in the last universal common ancestor^7^. The DMSO reductase superfamily probably underwent diversification during the Archaean–Paleoproterozoic oxygenation interval, providing evolving bacterial lineages and Eukaryota with a panoply of energetic solutions to the redox and terminal electron acceptor diversification that arose following the introduction of O_2_ in the Great Oxidation Event (GOE) of the Paleoproterozoic Era.

DMSO reductase family members sustain bacterial energy conservation under O_2_-limiting conditions by serving as high-potential terminal reductases that keep quinone-based respiration running, thereby preserving redox balance, proton motive force and ATP synthesis. DMSO reductase family members are, however, not limited to cellular energetics. Recent investigations have revealed that MopB-family members contribute to *Salmonella* pathogenesis in ways that are independent of traditional ATP synthesis. For example, the canonical DMSO reductase encoded in the *dmsABC* operon reduces methionine sulfoxide generated from the reaction of phagocyte NADPH oxidase-engendered reactive oxygen species (ROS) and host cell methionine^6^. Reduction of methionine sulfoxide by the DmsABC enzymatic complex helps *Salmonella* balance redox, controls pH homeostasis and promotes H_2_S synthesis. H_2_S is also synthesized by the MopB-family member thiosulfate reductases^8^. The bioactive H_2_S gas increases *Salmonella* fitness by inhibiting members of the resident microbiota and promoting antioxidant defence^6,8^.

The quintessential anaerobic fumarate nitrate reduction (FNR) regulator is key to the adaptation of intracellular *Salmonella* to oxidative products of the phagocyte NADPH oxidase^9^, but the mechanisms by which this regulator shields *Salmonella* from the noxious effects of oxidative stress are incompletely understood. The four paralogues of *dmsA* (*STM14_1809*, *STM14_1811*, *STM14_3103* and *STM14_5178*) encoded in the *S.* Typhimurium genome are among the most activated loci by FNR^9^. The function of the four additional *dmsA* paralogues is currently unknown. We undertook genetic, biochemical and biophysical approaches to identify the roles and substrate preference of three *dmsA* paralogues in non-typhoidal *Salmonella*.

## Results

### DmsA paralogues enable colonization of gut and viscera

**Fig. 1:**
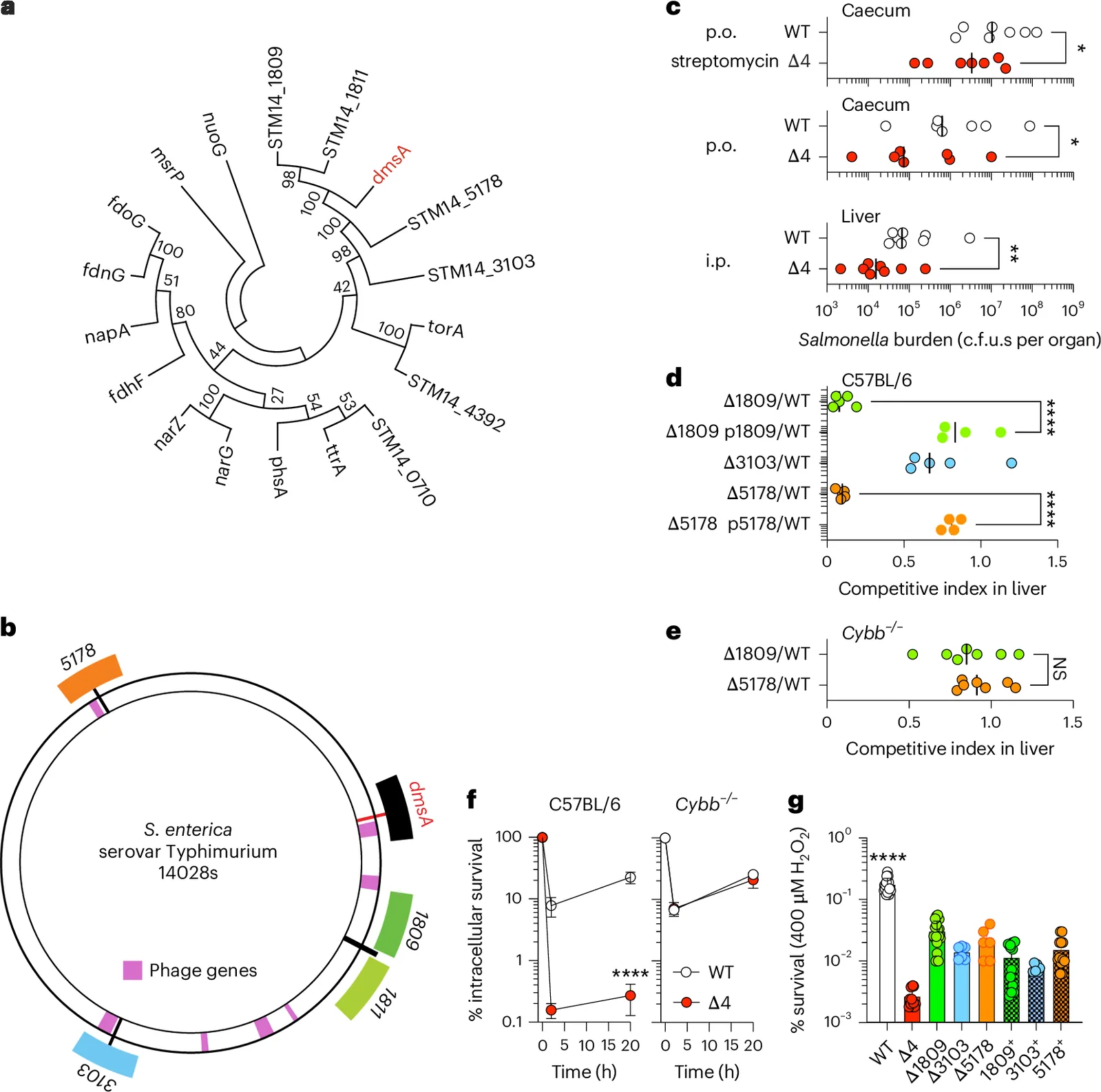
DmsA paralogues protect *Salmonella* against the phagocyte NADPH oxidase. **a**, Phylogenetic tree constructed using neighbour joining in MEGAx software with 500 bootstrap replicates based on amino-acid sequences of the DMSO reductase family members in the genome of *S. enterica* serovar Typhimurium strain 14028s. Branch lengths in the tree are proportional to evolutionary distances calculated using the Poisson correction method. Numbers in the tree represent the bootstrap values. **b**, Mapping of *dmsA* paralogues in the genome along with adjacent prophage elements. **c**, Δ4 indicates deletion of all four *dmsA* paralogues. **c**–**e**, *Salmonella* strains were evaluated for virulence in 6–8-week-old C57BL/6 (**c**,**d**) and *Cybb*^*−/−*^ (**e**) mice. Male and female mice were assigned randomly to the experimental groups. Bacterial fitness was assessed after 4 (**c**; p.o.) or 3 days (i.p., **d**,**e**) post-infection. Data are mean ± s.d. (**c**, *n* = 7, 7, 7, 7, 9, 9; **d**, *n* = 5, 4, 5, 5, 4; **e**, *n* = 7,7). **f**, Percent survival of *Salmonella* in periodate-elicited macrophages from C57BL/6 or *Cybb*^−/−^ mice with peritoneal exudate cells grown at 1% O_2_. Data are mean ± s.d. (C57BL/6 *n* = 7; *Cybb*^−/−^ *n* = 10). **g**, Survival of *Salmonella* after 2 h of treatment with 400 μM H_2_O_2_. Some strains carried a single *dmsA* paralogue in their genomes (for example, *1809*^*+*^). Data are mean ± s.d. (*n* = 18, 12, 18, 6, 6, 12, 6, 12). NS, not significant; *P < 0.5, **P < 0.01, ****P < 0.0001 by two-tailed paired *t*-test (**c**), two-tailed unpaired *t*-test (**e**), two-way ANOVA (**f**, d.f. = 1, *F* = 36.63) or one-way ANOVA (**d**, d.f. = 8, *F* = 32.58; **g**, d.f. = 82,6, *F* = 18.79). Source data

**Extended Data Fig. 1:**
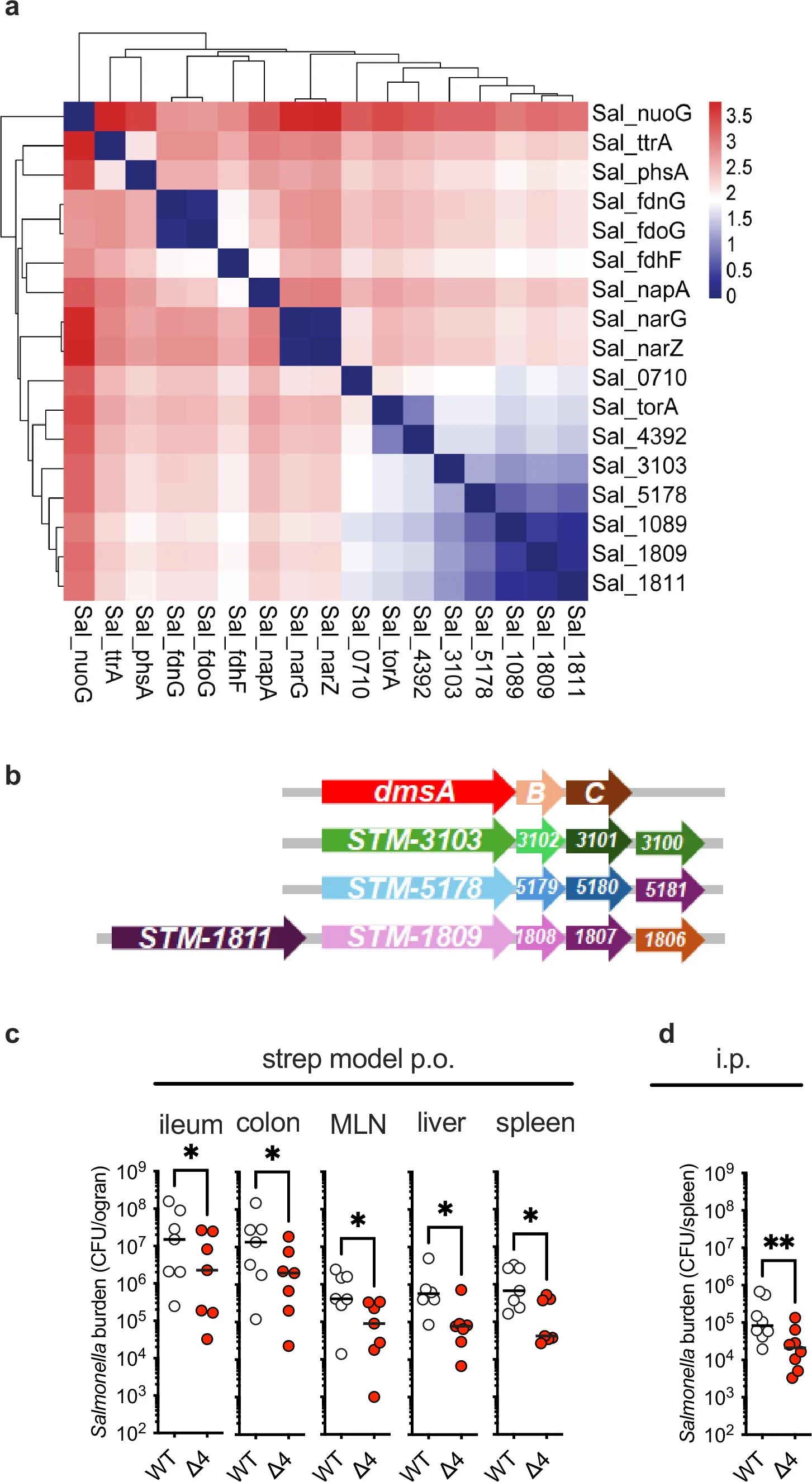
Operon organization of DmsA paralogs and their contribution to *Salmonella* fitness. **a**. Hierarchical clustering and heatmap analysis of *Salmonella* MopB-family proteins. Pairwise evolutionary distances were calculated as patristic distances derived from a maximum-likelihood phylogeny inferred from a trimmed multiple sequence alignment. Hierarchical clustering using average linkage reveals distinct MopB subfamilies corresponding to known respiratory enzyme systems. Heatmap colors represent evolutionary distances. **b**. Scheme of *dmsA* paralog operons. **c**. Competition of Δ*4* (quadruple mutant in all four *dmsA* paralogs) and wild-type *Salmonella* in the ileum, colon, MLN, liver, and spleen of C57BL/6 mice in a streptomycin-treated model of oral infection. The bacterial burden was assessed by calculating the CFUs 4 days post-infection after p.o. inoculation. The data are mean ± SD from 7 mice in 2 independent experiments. **d**. Virulence of Δ4 *Salmonella* was assessed by counting bacterial burden in the spleens of C57BL/6 3 days after i.p. inoculation of ~500 CFU mixture of equal numbers of wild-type and mutant *Salmonella*. The data are presented as mean ± SD from 8 mice in 2 individual experiments. *, **, *P* < 0.1, 0.01, respectively, as estimated by paired *t*-test (**c, d**). Source data

**Extended Data Fig. 2:**
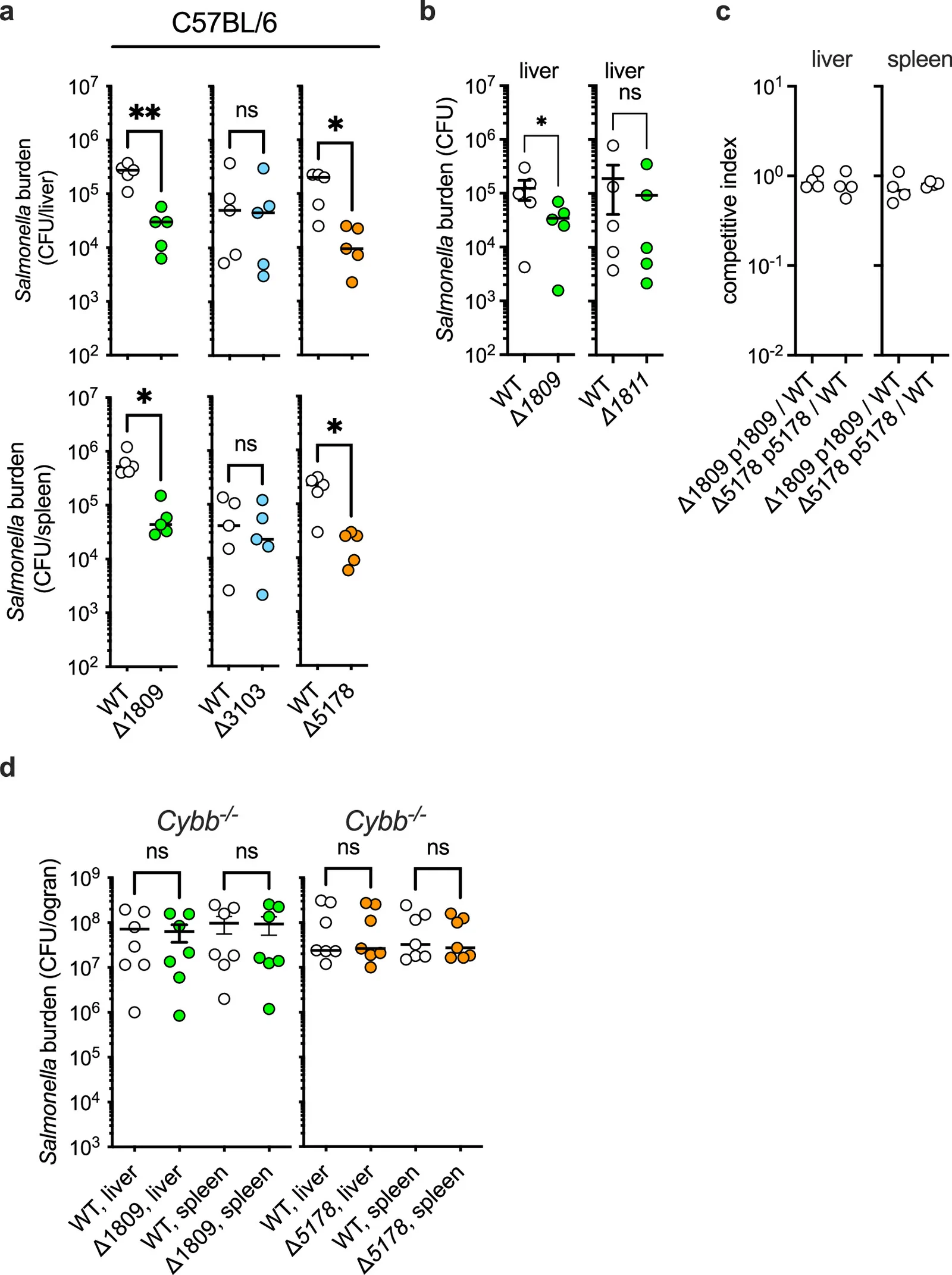
DmsA paralogs enhance *Salmonella* resistance to phagocyte NADPH oxidase-driven oxidative stress. **a, b, c, d**. The bacterial burden was quantified in the spleens and livers of C57BL/6 mice 3 days after i.p. inoculation with ~300 CFU of a mixture containing equal numbers of wild-type *Salmonella* and isogenic mutants (**a, b, d**). Competitive index (**c**) was calculated 3 days after i.p. infection. Data represent mean ± SD from 2 independent experiments (a liver and spleen n = 5 mice; **b** n = 4 mice; c n = 5 mice; d liver and spleen n = 7 mice). *, **, *P* < 0.1, 0.01 as estimated by paired *t*-test (**a, b, c**) or one-way ANOVA (**d**). Source data

**Extended Data Fig. 3:**
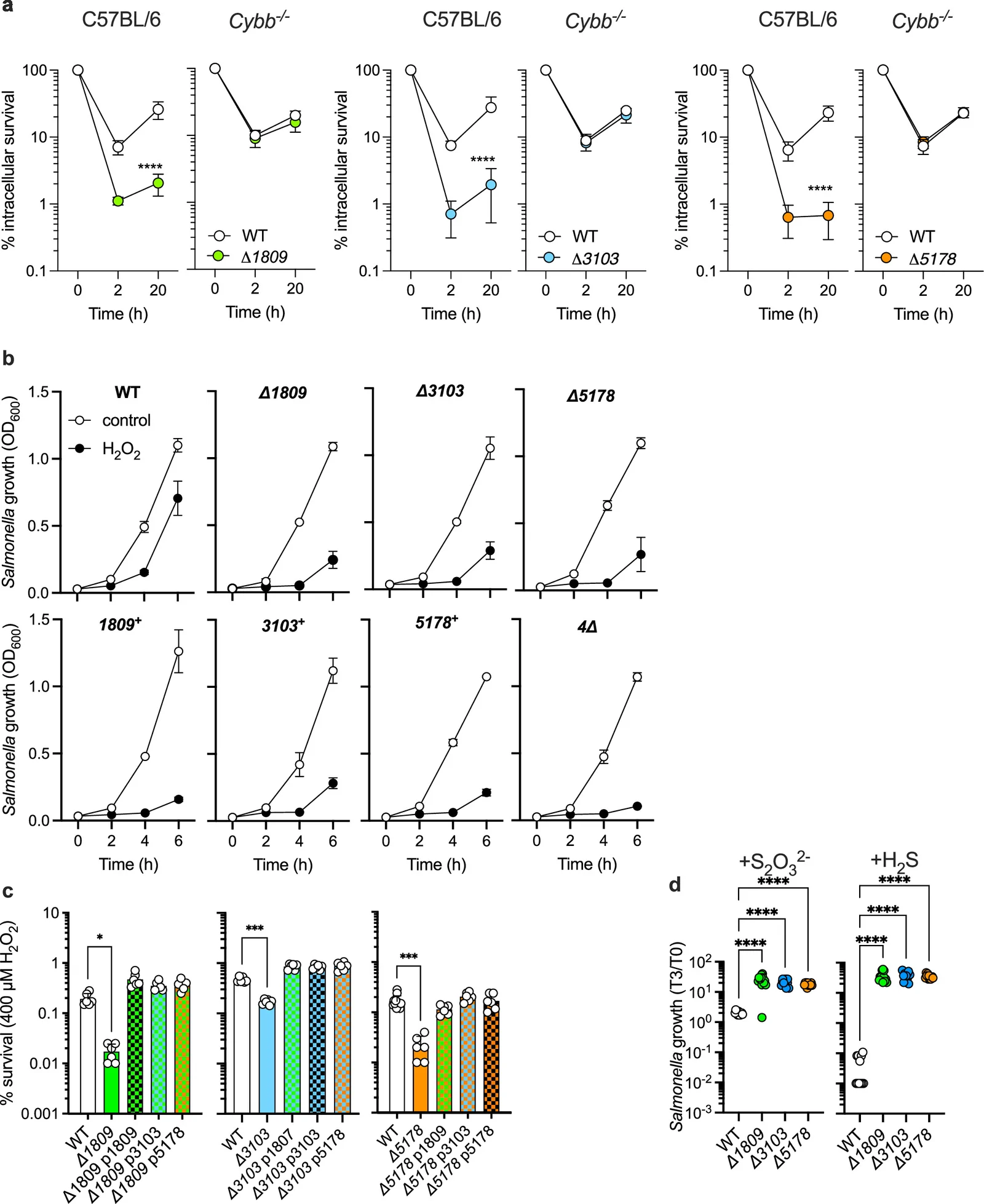
Susceptibility of *dmsA* paralog mutants to oxidative stress. **a**. The percent survival of single *dmsA* paralog deletion mutants was assessed in periodate-elicited macrophages from C57BL/6 and *Cybb*^−/−^ mice. CFU were enumerated at 0, 2, and 20 h post-infection to determine bacterial replication. The data are mean ± SD from 2 independent experiments (C57BL/6, n = 7; *Cybb*^−/−^, n = 10). **b**. Growth, as measured by OD_600_, of *dmsA* paralog mutants in MOPS-GLC-sulfate minimal medium supplemented with 400 μM H_2_O_2_. The strains carried single (for example, Δ1809) or quadruple (that is, Δ4) mutations in *dmsA* paralogs. Strains expressing single *dmsA* paralogs in the genome (for example, 1809^+^) were constructed by deleting the other two paralogs. Data are represented as mean ± SD from 2 biological replicates in 2 independent experiments. **c**. Single *dmsA* paralog deletion mutants were cultured aerobically in LB broth at 37 °C and challenged with 400 μM H_2_O_2_ in PBS for 2 h. Bacterial survival was determined by CFU enumeration. Cross-complementation was performed using the *dmsA* paralog cloned into the low-copy plasmid pWSK29. The data are mean ± SD; n = 6 (right panel WT n = 12) from 3-4 individual experiments. *, ***, **** *P* < 0.1, 0.001, 0.0001, respectively, were calculated by two-way (**a**) or one-way ANOVA (**c**). **d**. Effect of exogenous addition of 100 μM sodium thiosulfate (Na_2_S_2_O_3_) or sodium sulfide (Na_2_S) on the growth of wild-type and *dmsA* paralog *Salmonella* mutants. Mutants were cultured in MOPS-GLC medium containing thiosulfate or sulfide for 3 h at 37 °C under ambient air without shaking. Bacterial viability was assessed by CFU enumeration. The data are mean ± SD; n = 12 from 3 independent experiments. **** *P* < 0.000 by one-way ANOVA. Source data

Our recent investigations have demonstrated that the *dmsA* gene product (STM14_1089*)*, in addition to its canonical DMSO reductase activity, acts as a methionine sulfoxide reductase^6^. The *Salmonella* genome encodes four additional *dmsA* paralogues organized in three distinct gene clusters (Fig. 1a,b, Extended Data Fig. 1a,b and Supplementary Fig. 1). We hypothesized that *Salmonella* may utilize the uncharacterized *dmsA* paralogues during its associations with vertebrate hosts. To test this idea, we used λ-Red recombination to construct a strain deficient in the operons of all four paralogues (designated ‘Δ4’). The Δ4 *Salmonella* strain was attenuated when inoculated orally (p.o.) into streptomycin-treated or untreated C57BL/6 mice together with equal numbers of wild-type (WT) isogenic bacteria (Fig. 1c and Extended Data Fig. 1c), suggesting that the *dmsA* paralogues help *Salmonella* colonize the gut of mice across conditions of microbiota integrity and antibiotic-induced dysbiosis. The Δ4 *Salmonella* strain was also attenuated after intraperitoneal (i.p.) inoculation (Fig. 1c and Extended Data Fig. 1d), indicating that the gene products of the *dmsA* paralogues not only enhance *Salmonella* gut colonization but also enable its establishment in deep tissues. Individual *dmsA* paralogue mutants revealed that proteins encoded by *STM14_1809* and *STM14_5178* contributed to a greater extent to the fitness of *Salmonella* in systemic tissue than *STM14_1811* and *STM14_3103* (Fig. 1d and Extended Data Fig. 2a,b). The ΔSTM14_1809 and ΔSTM14_5178 *Salmonella* strains recovered fitness in C57BL/6 mice when complemented with the low-copy pWSK29 plasmid bearing the *STM14_1811–1807* or *STM14_5178–5180* operons (Fig. 1d and Extended Data Fig. 2c). The ΔSTM14_1809 and ΔSTM14_5178 mutant strains also became virulent in *Cybb*^−/−^ mice (Fig. 1e and Supplementary Fig. 2d) and *Cybb*^−/−^ macrophages (Extended Data Fig. 3a) deficient in the gp91*phox* subunit of the phagocyte NADPH oxidase^10,11^, suggesting that the products of *STM14_1809* and *STM14_5178* confer a selective advantage in the oxidative environment generated by the respiratory burst of myeloid cells. The Δ4 strain was also attenuated in *Cybb* suficient macrophages (Fig. 1f). The Δ4 strain was more susceptible to bactericidal concentrations of hydrogen peroxide (H_2_O_2_) than any of the single *dmsA* paralogue mutants (Fig. 1g). *Salmonella* strains expressing single *dmsA* paralogues (for example, *1809*^+^) were also hypersusceptible to the bacteriostatic activity of H_2_O_2_ **(**Extended Data Fig. 3c). Single *dmsA* paralogue mutants complemented with the low-copy pWSK29 plasmid expressing *STM14_1809*, *STM14_3103* or *STM14_5178* operons became as resistant to H_2_O_2_ killing as WT *Salmonella* (Extended Data Fig. 3b). Together, these results demonstrate that the presence of additional dmsA-family members has enhanced the capacity of *Salmonella* to withstand the oxidative stress generated by the phagocyte NADPH oxidase. Our investigations suggest that the additional dmsA paralogues may each play similar roles in *Salmonella* resistance to oxidative stress.

### DmsA paralogues facilitate extracellular sulfate utilization

**Fig. 2:**
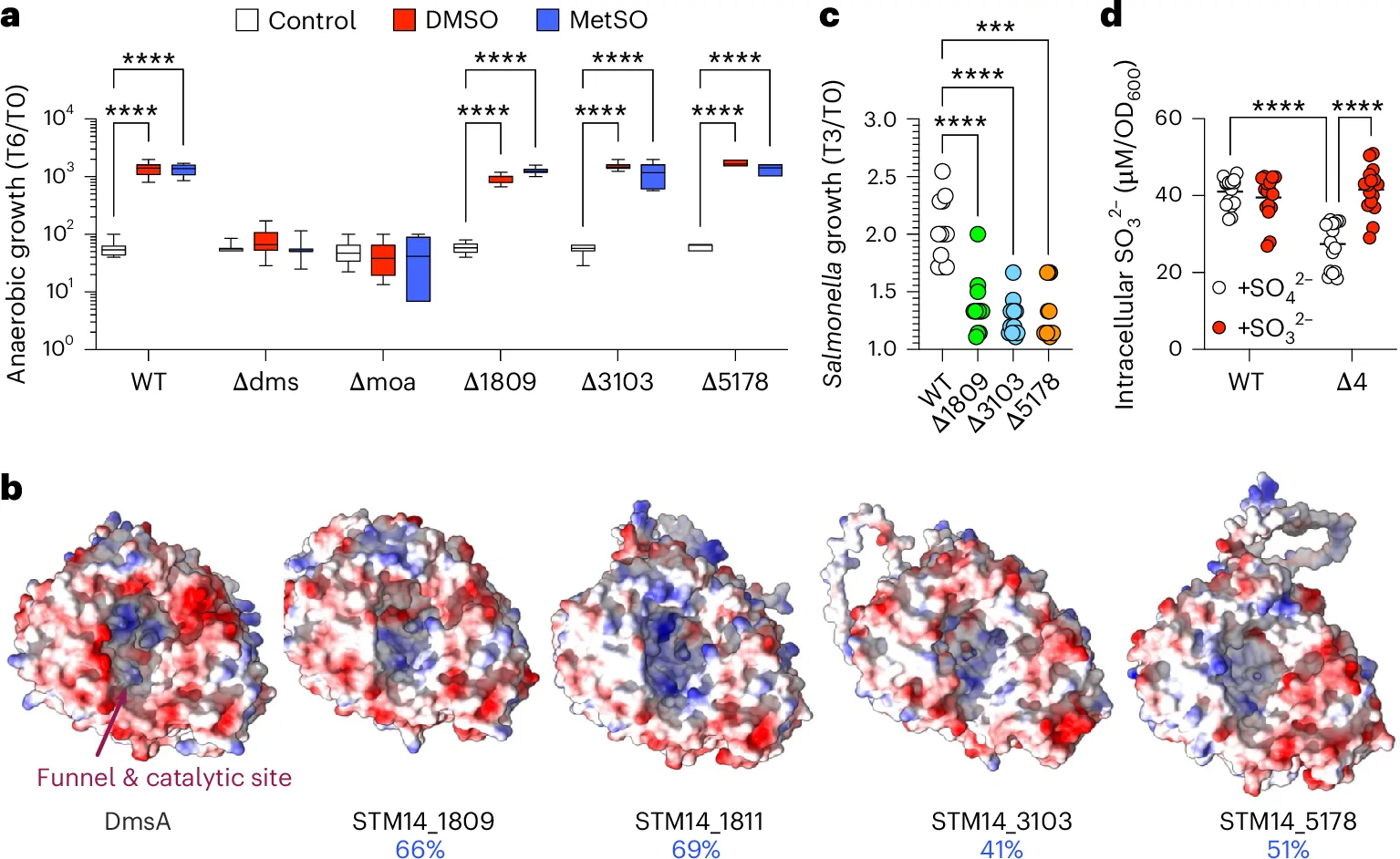
DmsA paralogues exhibit sulfate reductase activity. **a**, Anaerobic growth of *Salmonella* in MOPS-CAA medium containing 40 mM DMSO or L-methionine sulfoxide (L-MetSO). Data are mean ± s.d. (*n* = 12 (Δ1809) and *n* = 6 (Δ5178)). **b**, Surface electrostatic potential maps of DmsA paralogues were predicted using AlphaFold_3_ and visualized with UCSF ChimeraX. DmsA was used as the reference structure. Electrostatic surfaces are depicted, with negative potentials in red and positive potentials in blue. The percentage identity of each paralogue relative to DmsA is shown in blue. **c**, Growth of *Salmonella* in MOPS-GLC medium at 37 °C for 3 h under aerobiosis without shaking. Data are the mean ± s.d.; *n* = 12; d.f. = 11, *F* = 25.61. **d**, Intracellular sulfite concentrations of wild-type and Δ4 *Salmonella* strains grown overnight in MOPS-GLC medium supplemented with 250 µM of either sulfate or sulfite under ambient air without shaking. Data are mean ± s.d. of 2 individual experiments (*n* = 16). Symbols in **a**, **c** and **d** depict biological replicates. ***P < 0.001, ****P < 0.0001 by two-way ANOVA (**a**, d.f. = 2, *F* = 309.8; **d**, d.f. = 1, *F* = 17.82, 21.24), or one-way ANOVA (**c**, d.f. = 3, *F* = 25.61). Source data

On the basis of the substrates used by the DmsABC enzymatic complex^6^, we tested whether the DmsA paralogues reduce methionine sulfoxide or DMSO. Strains bearing deletions in *STM14_1809, STM14_3103* and *STM14_5178* gene clusters respired anaerobically on DMSO or methionine sulfoxide as efficiently as wild-type controls (Fig. 2a), suggesting that the DmsA paralogues have different substrate preferences from the DmsABC complex. Consistent with the possibility that these enzymes exhibit substrate preferences distinct from those of DmsA, the DmsA paralogues share ~40–70% amino-acid identity (Fig. 2b and Supplementary Fig. 1) and display notable charge differences in the funnel leading to the MOCO-catalytic site (Fig. 2b). Mutants lacking *STM14_1809, STM14_3103* and *STM14_5178* consistently grew poorly in MOPS-glucose (GLC) media when compared to wild-type controls (Fig. 2c), suggesting that DmsA paralogues may enable *Salmonella* to utilize a substrate present in this medium. Sulfate is the only sulfur donor in the MOPS-GLC medium, suggesting that the absence of *dmsA* paralogues may impair its utilization. The Δ4 *Salmonella* strain did, however, grow to higher cell densities than wild-type *Salmonella* controls in MOPS-GLC medium supplemented with the sulfur donor thiosulfate or hydrogen sulfide (H_2_S) (Extended Data Fig. 3d), suggesting that the DmsA paralogues may aid with the utilization of sulfate. Further supporting this idea, the Δ4 *Salmonella* strain growing in sulfate as the sole sulfur source contained lower levels of intracellular sulfite than wild-type controls (Fig. 2d). Wild-type and Δ4 *Salmonella* grown in MOPS-GLC medium containing sulfite as sole sulfur source harboured similar intracellular concentrations of sulfite (Fig. 2d).

### DmsA paralogues are extracytoplasmic sulfate reductases

**Fig. 3:**
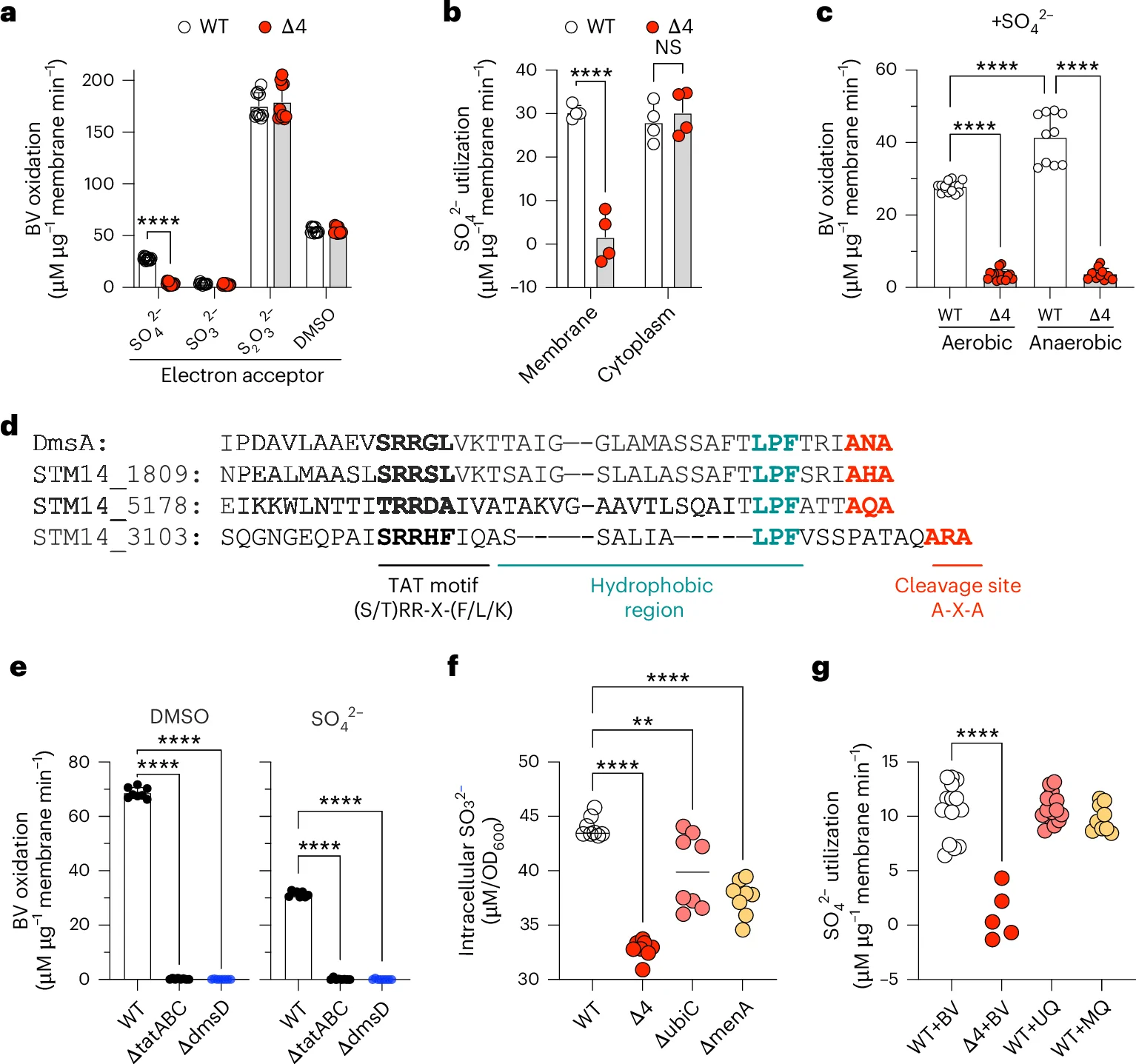
DmsA paralogues are extracytoplasmic sulfate reductases. **a**,**c**,**e**, Reductase activity in membranes isolated from the indicated *Salmonella* strains was measured spectrophotometrically at 570 nm by monitoring benzyl viologen (BV) oxidation in reactions containing sulfate (SO_4_^2−^), sulfite (SO_3_^2−^), thiosulfate (S_2_O_3_^2−^) or DMSO. Membranes were obtained from bacteria grown in LB broth under aerobic (**c**) or anaerobic (**a**, **c**, **e**) conditions. Data are represented as mean ± s.d. of 5–6 independent experiments (**a**, sulfate *n* = 14; sulfite, thiosulfate and DMSO *n* = 10; **c**, aerobic *n* = 14, anaerobic *n* = 10; **e**, *n* = 8). **b**, Evaluation of sulfate reduction from the membrane and cytoplasm. Sulfate utilization was measured in isolated membrane and cytoplasmic fractions of *Salmonella*. Data represent mean ± s.d.; *N* = 4 from 2 individual experiments. **d**, The N-terminal signal peptides targeting the twin-arginine translocation (TAT) of DmsA, STM14_1809, STM14_5178 and STM14_3103 were aligned using Clustal W after predicting the TAT signal peptide and cleavage site using SignalP (v.5.0). TAT signal peptides contain a highly conserved twin-arginine (RR) motif (SRRXFLK), a hydrophobic region and an AXA cleavage site. **f**, Intracellular sulfite concentrations were quantified in *Salmonella* grown overnight in MOPS-GLC medium supplemented with sulfate under ambient air without shaking. Data are mean ± s.d. of 8 independent experiments. **g**, Sulfate reductase activity in isolated membranes was evaluated by quantifying sulfate utilization in the presence of different electron carriers. The enzyme activity demonstrated dependency on both ubiquinone (UQ) and menaquinone (MQ), while benzyl viologen (BV) served as a control. In the reaction, UQ and MQ compounds were treated at a concentration of 1 mg ml^−1^. Data in **g** are mean ± s.d.; *n* = 8 from 2–3 independent experiments. Symbols in **a**–**c**, **e** and **g** depict biological replicates. **P < 0.01, ****P < 0.0001 by two-way ANOVA (**a**, d.f. = 1, *F* = 10.50; **b**, d.f. = 1, *F* = 34.76; **c**, d.f. = 13,3, *F* = 1.22, 361.2) or by one-way ANOVA (**e**, d.f. = 2, *F* = 9672; **f**, d.f. = 3, *F* = 43.73; **g**, d.f. = 3, *F* = 35.37). Source data

**Extended Data Fig. 4:**
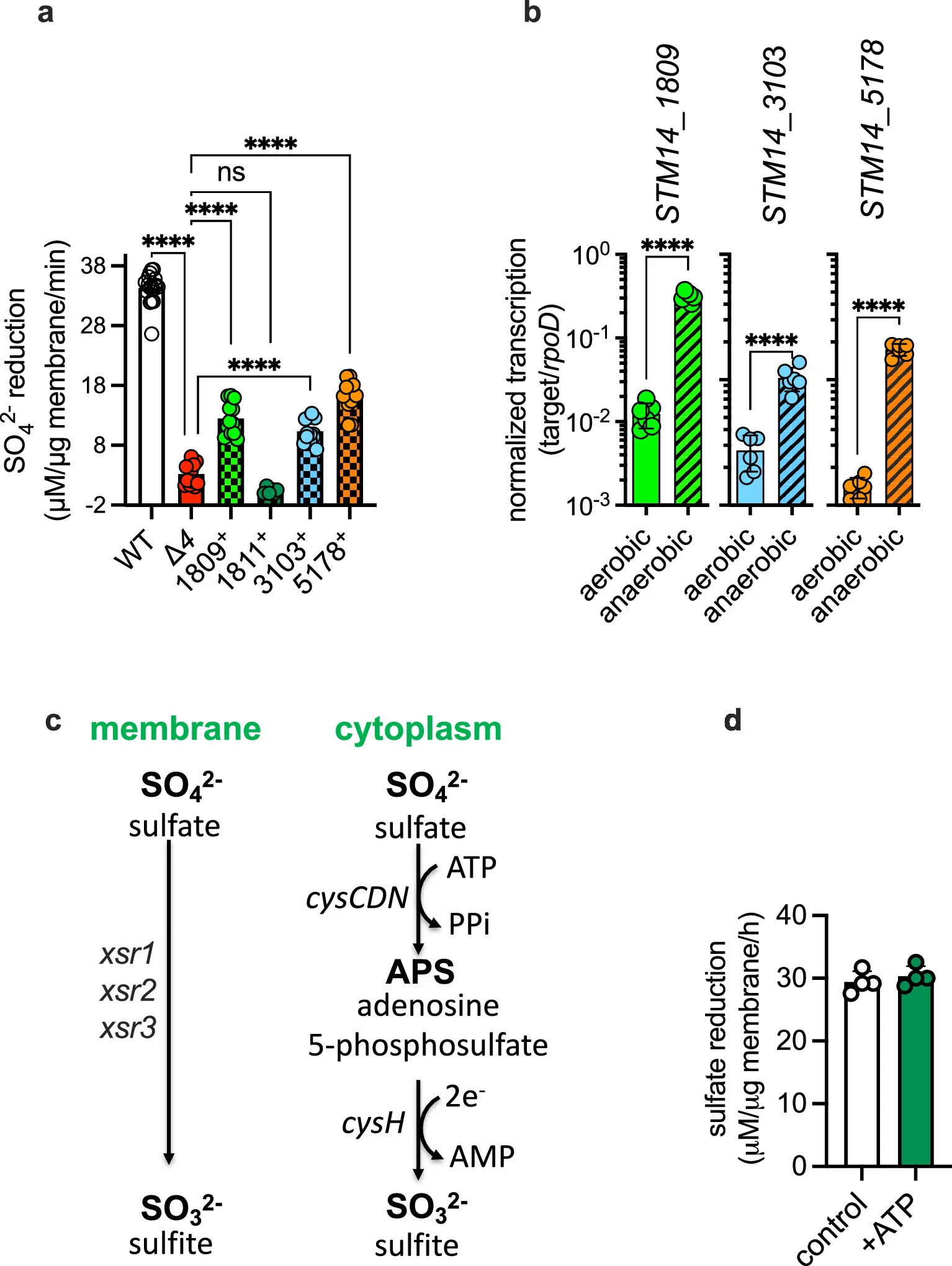
DmsA paralogs function as membrane-associated sulfate reductases, indirectly facilitating sulfur acquisition to support the growth of *Salmonella*. **a**. Sulfate reductase activities were measured in membranes isolated from the indicated strains. The strains carried quadruple (that is, Δ4) mutations in *dmsA* paralogs. Strains expressing a single *STM14_1809, STM14_3103 or STM14_5178* paralog gene within the genome (for example, 1809^+^) were constructed by deleting the remaining three *dmsA* paralog genes. The Δ*4* p1811 strain was constructed by complementing the Δ4 strain with the pWSK29 plasmid bearing the *STM14_1811* gene. Sulfate reductase activity in isolated membranes was evaluated by quantifying sulfate utilization in the presence of sulfate. The data are mean ± SD; n = 12 from 4 independent experiments. **b**. Transcripts of *dmsA* paralog genes were quantified by real-time qRT-PCR using RNA isolated from *Salmonella* grown aerobically or anaerobically in MOPS-GLC-minimal medium supplemented with 250 μM sulfate. The data are mean ± SD; n = 6 from 2 independent experiments. **c**. Pathway of sulfate reduction in the cell membrane and cytoplasm of *Salmonella*. **d**. Evaluation of ATP-dependent sulfate reduction. Sulfate utilization was measured in isolated membrane fractions of *Salmonella* in the presence of 0.2 mM ATP. Data represent mean ± SD; n = 4 from 2 individual experiments. **** *P* < 0.0001 as estimated by one-way ANOVA (**a, b**) or unpaired *t* test (**d**). Source data

Given the apparent deficiency of the Δ4 strain in sulfate utilization, we tested whether DmsA paralogues may facilitate anaerobic respiration of this naturally occurring and abundant oxyanion. We monitored the oxidation of the electron donor benzyl viologen by membranes isolated from wild-type or dmsA paralogue-deficient *Salmonella* in the presence of various terminal electron acceptors. Membranes from Δ4 *Salmonella* respired normally on thiosulfate or DMSO (Fig. 3a), demonstrating that PhsABC and DmsABC members of the DMSO reductase superfamily are functional in the absence of *STM14_1811*, *STM14_1809, STM14_3103* and *STM14_5178*. However, membranes isolated from the Δ4 *Salmonella* strain failed to respire on sulfate (*P* < 0.0001) (Fig. 3a), whereas membranes expressing a single *STM14_1809-*, *STM14_3103-* and *STM14_5178-*encoded DmsA paralogue supported intermediate levels of sulfate reductase activity (Extended Data Fig. 4a). Cumulatively, these data indicate that *STM14_1809, STM14_3103* and *STM14_5178* gene products encode hitherto unknown sulfate reductases. In sharp contrast, membranes from the Δ4 strain expressing the *STM14_1811* gene lacked sulfate reductase activity, although they exhibited normal DMSO reduction (Extended Data Fig. 4b). These findings suggest that the DmsA paralogue encoded by the *STM14_1811* locus does not support sulfate reductase activity. Given these results, the STM14_1811 gene product was not further studied here.

Cytoplasmic extracts isolated from Δ4 *Salmonella* and wild-type controls exhibited comparable sulfate reductase activity (Fig. 3b), indicating that the cytoplasmic sulfate reductase CysIJ is expressed normally in the Δ*4 Salmonella* strain. Membranes of aerobically and anaerobically grown *Salmonella* reduced sulfate, with the latter exhibiting higher activity (Fig. 3c). The increased sulfate reductase activity present in membranes isolated from anaerobically grown *Salmonella* may reflect the induction of *STM14_1809*, *STM14_3103* and *STM14_5178* genes (Extended Data Fig. 4b). Collectively, these findings indicate that membrane-bound extracytoplasmic sulfate reductases are active aerobically and, more prominently, anaerobically.

The catalytic subunit of DMSO reductase family members is secreted into the periplasm via the twin-arginine (RR) secretion system^12^. RR signatures and cleavage sites in the N terminus of STM14_3103, STM14_5178 and STM14_1809 are consistent with the idea that these oxidoreductases are substrates of the TAT secretion system (Fig. 3d). As previously observed for the DmsABC enzymatic complex, sulfate reductase activity was completely lost in membranes isolated from a Δ*tatABC* mutant (Fig. 3e, right panel), supporting the idea that the membrane-bound sulfate reductases encoded in the *STM14_3103*, *STM14_5178* and *STM14_1809* operons are substrates of the TAT secretion system. As expected^13^, DMSO reductase requires the TAT secretion system and the DmsD chaperone encoded in *STM14_1806* (Fig. 3e, left panel). Deletion of *dmsD* also abolished sulfate reductase activity in the membranes (Fig. 3e, right panel). Collectively, the bioinformatic, genetic and biochemical analyses support the idea that the sulfate reductases described here are secreted into the periplasm.

We next tested whether membrane-bound sulfate reductases draw reducing power from quinones. To test this idea, we measured sulfate reductase activity in membranes isolated from Δ*ubiC* or Δ*menA Salmonella*, which are deficient in key steps in the biosynthesis of ubiquinone or menaquinone, respectively^14^. Membranes isolated from ΔubiC and ΔmenA *Salmonella* strains harboured intermediate sulfate reductase levels (Fig. 3f), suggesting that extracytoplasmic sulfate reductases can draw electrons from either ubiquinone or menaquinone. In support of this notion, the addition of ubiquinone or menaquinone supported excellent sulfate utilization by membrane-bound sulfate reductases in a biochemically reconstituted in vitro system (Fig. 3g). These experiments demonstrate that extracytoplasmic sulfate reductases can use either menaquinone or ubiquinone, a situation previously reported for periplasmic nitrate reductase^15^. The enzymology of the sulfate reductase present in the membrane appears to involve a catalytic mechanism that is different from the cytoplasmic CysIJ pathway, in which reduction of sulfate to sulfite involves ATP-dependent synthesis of adenosine-5’-phosphosulfate and 3’-phosphoadenosine 5’-phosphosulfate intermediates (Extended Data Fig. 4c and ref. ^16^). Accordingly, the addition of ATP did not enhance the sulfate reductase activity in *Salmonella* membranes (Extended Data Fig. 4d).

Cumulatively, the bioinformatic, genetic and biochemical data presented in this section strongly suggest that the open reading frames of unknown function encoded in the *STM14_3103*, *STM14_5178* and *STM14_1809* operons are extracytoplasmic sulfate reductases exported into the periplasm via the TAT secretion system. Therefore, henceforth, we named these loci extracytoplasmic sulfate reductases: *xsr1ABC, xsr2ABC* and *xsr3ABC* for the complexes encoded by the *STM14_3103*, *STM14_5178* and *STM14_1809* operons, respectively.

### Xsr3A relies on an [Fe–S] instead of the MOCO cofactor

**Fig. 4:**
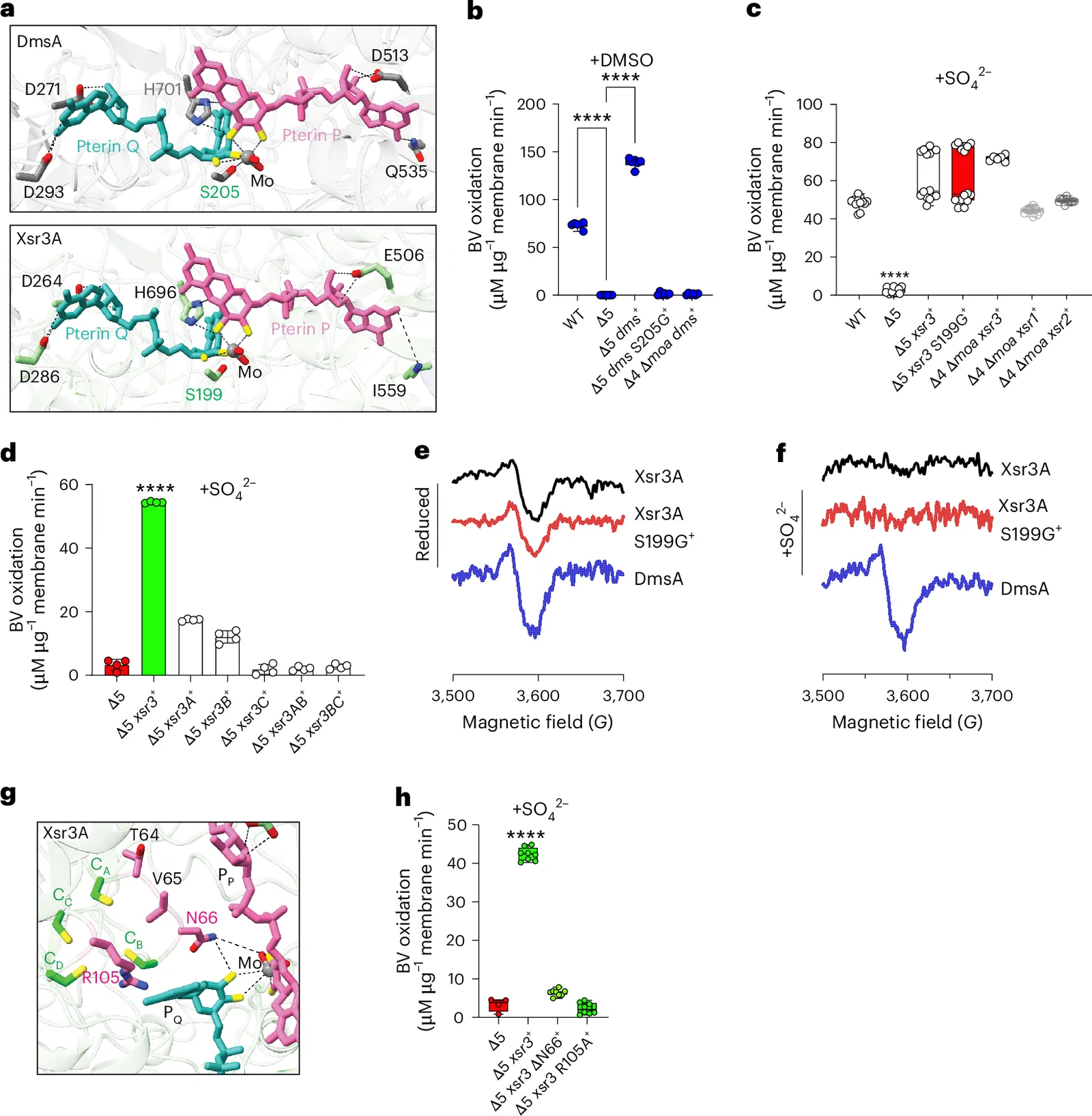
An iron–sulfur-dependent extracytoplasmic sulfate reductase in *Salmonella* functions independently of molybdenum cofactor. **a**, The predicted Mo-bis PGD is conserved in DmsA and Xsr3A. Coordinating serine residues (grey) and pterin (magenta and cyan)-interacting residues are shown. Distance to the active site is very similar (highlighted) in DmsA and the Xsr3A paralogue. **b**,**c**,**h**, The membranes used for EPR spectroscopy were evaluated for DMSO and sulfate reductase activities by measuring the oxidation of benzyl viologen (BV) in the presence of DMSO (**b**) or sulfate (**c**,**h**). The Δ5 strain carries a quintuple deletion of *dmsA* and its four paralogues. Data are mean ± s.d. of 2–7 independent experiments (**b**, *n* = 6; **c**, WT *n* = 12, Δ5 = 20, p*xsr3* *n* = 14, p*xsr3 S199G* *n* = 14, p*xsr3* Δ*moa* *n* = 6, p*xsr1* Δ*moa* *n* = 12, p*xsr2* Δ*moa* *n* = 12; **h**, Δ5 = 4, others *n* = 8). **c**, *Δ5 xsr3*^*+*^: minima 50.356, maxima 78.404, centre 60.021; *Δ5 xsr3 S199G*^*+*^: minima 45.754, maxima 80.090, centre 61.976. **h**, *Δ5*: minima 0.876, maxima 4.577, centre 3.305; *Δ5 xsr3*^*+*^: minima 40.265, maxima 44.591, centre 42.382; *Δ5 xsr3 R105A*^*+*^: minima 0.662, maxima 4.490, centre 2.368. **d**, Sulfate reductase activities were assessed by monitoring benzyl viologen oxidation in membranes isolated from the Δ*5* mutant and its complemented strains expressing individual genes, gene pairs or the entire *xsr3ABC* operon. Data presents mean ± s.d. of 2 independent experiments (*n* = 4). Data points in **d** depict biological replicates. **e**,**f**, EPR spectra of [4Fe–4S] clusters were obtained from membranes reduced with 2 mM dithionite, either alone (**e**, reduced) or in combination with 50 mM sulfate (**f**, SO_4_^2−^). ^∗∗∗∗^*P* < 0.0001 by one-way ANOVA (**b**, d.f. = 4, *F* = 2,916; **c**,**d**, d.f. = 6, *F* = 970; **h**, d.f. = 3, *F* = 1,349). **g**, Structural model of Xsr3A showing [Fe–S] coordination relative to the Mo-bis PGD prosthetic group. Conserved carbon atoms and residues, Asn^66^ and Arg^105^, interact with the pterin ring system. Source data

**Extended Data Fig. 5:**
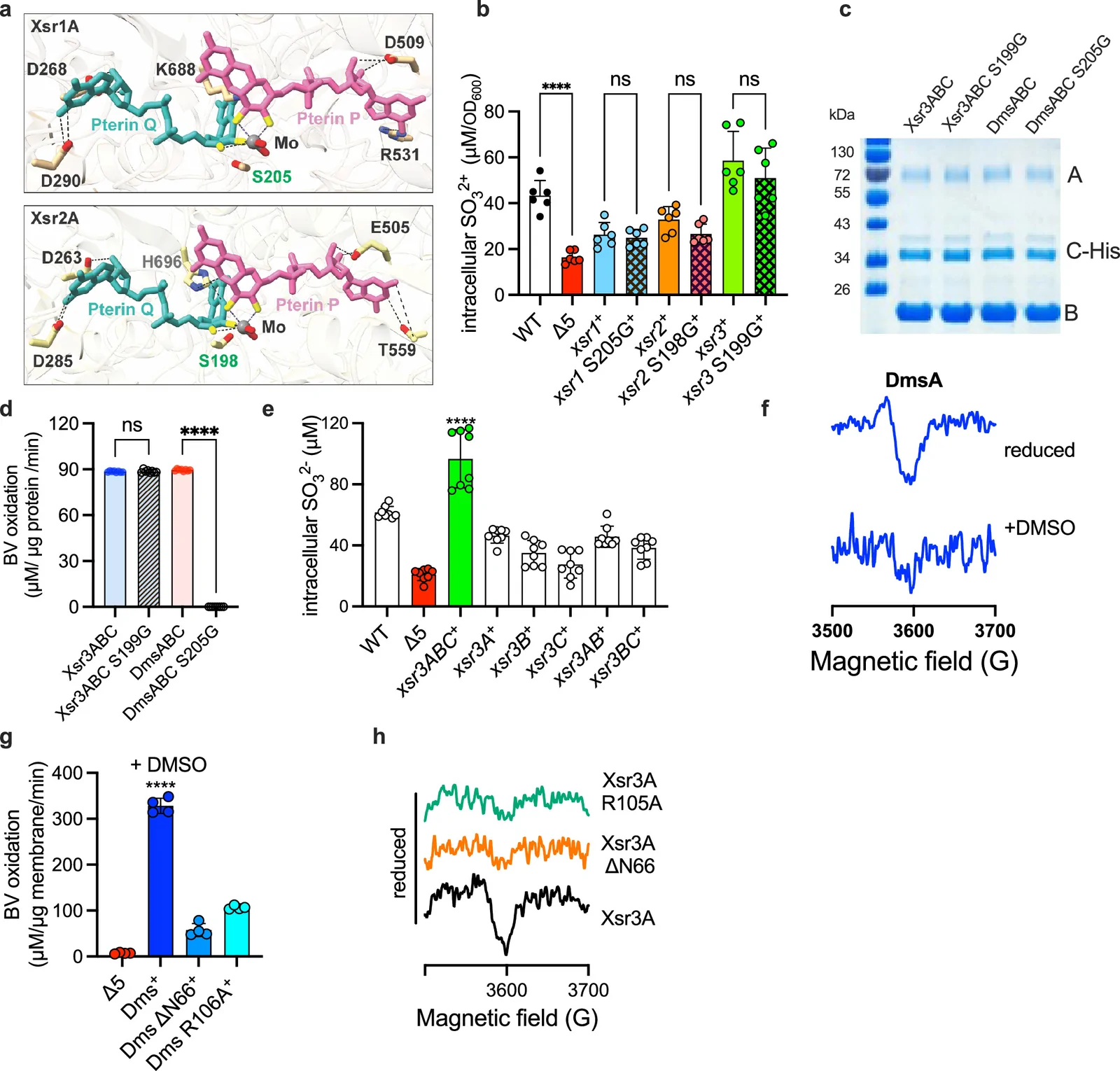
The function of periplasmic sulfate reductase in *Salmonella* is dependent on the FS0 [4Fe-4S], not the molybdenum cofactor. **a**. The predicted Mo-bis PGD in Xsr1A and Xsr2A as determined by AlphaFold_3_. **b, e**. Intracellular sulfite concentrations were measured in wild-type *Salmonella*, the Δ5 mutant deficient in *dmsA* and all 4 *dmsA* paralogs, and various complemented strains after overnight growth under hypoxic conditions in MOPS-GLC medium supplemented with sulfate. The data are presented as mean ± SD from 2 individual tests (**b**, n = 6; e, n = 8). **c**. Purified DmsABC, Xsr3ABC, and their variant protein complexes were separated on a 12% SDS-PAGE gel and visualized by Coomassie brilliant blue staining. **d, g**. Sulfate reductase activity was assessed by monitoring benzyl viologen (BV) oxidation in isolated membranes. The activity (**d**) was measured using purified DmsABC, Xsr3ABC, or their variant protein complexes. The Δ5 (**g**) mutant strains express individual *dmsABC* or *xsr3ABC* operon from a low-copy plasmid pWSK29. Data presents mean ± SD from 2 independent experiments (n = 4). **f, h**. EPR spectra of membranes isolated from Δ5 *Salmonella* strains expressing individual *dmsABC* (**f**) or *xsr3ABC* and its variants in low-copy plasmid pWSK29 were recorded either alone (reduced) or in the presence of 50 mM DMSO (**f**, +DMSO) after reduction with 2 mM dithionite. **** *P* < 0.0001, respectively, as estimated by one-way ANOVA (**b, d, e, g**). Source data

In the following investigations, we extended the biochemical and biophysical characterization of the Xsr extracytoplasmic sulfate reductases. The catalytic molybdenum in DMSO reductases is hexacoordinated by the dithiolenes of the pterin guanosine dinucleotide, the hydroxy side chain of a nearby serine residue and the oxygen in the substrate^17^. The key serine residue is conserved in DmsA, Xsr1A, Xsr2A and Xsr3A (Fig. 4a and Extended Data Fig. 5a). Complementation of the Δ5 strain deficient in *dmsABC* and the 4 *dmsABC* paralogues with a low-copy plasmid expressing the wild-type or *dmsA* S205G variant revealed that the coordinating Ser^205^ is vital for the reduction of DMSO by DmsA (Fig. 4b). Supporting the essential role of the MOCO cofactor in DMSO reductase activity, reduction of DMSO by DmsABC required the products of the *moaABCDE* operon (Fig. 4b). However, unexpectedly, the corresponding serine residues were irrelevant for the sulfate-reducing capacity of Xsr1A, Xsr2A and Xsr3A (Fig. 4c and Extended Data Fig. 5b). To independently assess the role of the Mo-coordinating serine residue in the catalytic activity of DmsA paralogues, we purified DmsABC and XsrABC complexes from membranes of *Salmonella* grown under anaerobic conditions (Extended Data Fig. 5c). These data confirmed that the Mo-coordinating serine residue is vital for DmsABC activity but dispensable for Xsr3ABC (Extended Data Fig. 5d). Even more unexpectedly, the *moaABCDE* gene products, which catalyse MOCO cofactor synthesis, were dispensable for the activity of the extracytoplasmic sulfate reductases Xsr1, Xsr2 and Xsr3 (Fig. 4c). Together, these data demonstrate that, in contrast to canonical DMSO reductase, extracytoplasmic sulfate reductases do not use the MOCO cofactor during catalysis.

Given the unexpected dispensability of the MOCO cofactor for the activity of the Xsr paralogues, we examined whether catalytic (A), electron-transfer Fe–S (B) and quinone-interacting, membrane (C) subunits are needed for sulfate reductase activity. Because Xsr3 was the most similar to DmsABC, we chose Xsr3 for in-depth analysis. All A, B and C subunits were needed for the enzymatic activity of Xsr3 in vivo or in membrane fractions (Fig. 4d and Extended Data Fig. 5e), suggesting that extracytoplasmic sulfate reductases must still utilize a redox-active centre at the A subunit. The [Fe–S] cluster is the only other redox centre present in the catalytic A subunit of DMSO reductase family members^18^. To determine whether the [Fe–S] cluster is responsible for the reduction of sulfate, we performed an electron paramagnetic resonance (EPR) biophysical approach. To minimize interference from biophysically related redox centres, a Δ5 strain lacking all *dmsA* and its four paralogues was used as the background in which a plasmid harbouring *dmsABC* was expressed. In parallel, the *xsr3ABC* operon was expressed in the Δ5 strain. The resulting strains were designated Δ5 dms^+^ and Δ5 xsr3^+^, respectively. Membranes isolated from Δ5 xsr3^+^ or Δ5 xsr3^+^ S199G *Salmonella* strains harboured the [Fe–S] EPR signal (Fig. 4e, top traces) characteristic of the canonical DmsA protein (Fig. 4e, bottom trace). The addition of sulfate oxidized the [Fe–S] redox centre of the wild-type Xsr3A and Xsr3A S199G variant, but not the DmsA protein (Fig. 4f, top two traces), demonstrating specific binding of sulfate to the [4Fe–4S] cluster of Xsr3A. These EPR findings also demonstrate that binding of sulfate to Xsr3A does not require the presence of a functional Mo-binding serine residue. The [Fe–S] of DmsA was, nonetheless, oxidized by its cognate DMSO electron acceptor (Extended Data Fig. 5f), demonstrating the specificity of DmsA for DMSO. These biophysical findings support the idea that the [Fe–S] of the Xsr3 paralogue is the site of sulfate reduction in this extracytoplasmic sulfate reductase. To test this model further, we deleted the asparagine residue of Xsr3A and DmsA at position 66, shortening the distance between Cys^A^ and Cys^B^ residues coordinating the [Fe–S], and independently introduced an alanine substitution in the bridge arginine contributing critical bonding to Cys in the D position (refs. ^19,20^, Fig. 4g). As expected, membranes harbouring the DmsA ΔAsn^66^ or DmsA R106A variants lack DMSO reductase activity (Extended Data Fig. 5g). Likewise, membranes isolated from a *Salmonella* strain expressing either Xsr3A ΔAsn^66^ or Xsr3A R105A variants were not only deficient in sulfate reductase activity (Fig. 4h) but also lacked the characteristic [Fe–S] EPR signal (Extended Data Fig. 5h). Together, these biochemical and biophysical approaches demonstrate that the [Fe–S] cluster of Xsr3A is directly involved in sulfate reduction.

### Xsr sulfate reductases foster sulfur assimilation

**Fig. 5:**
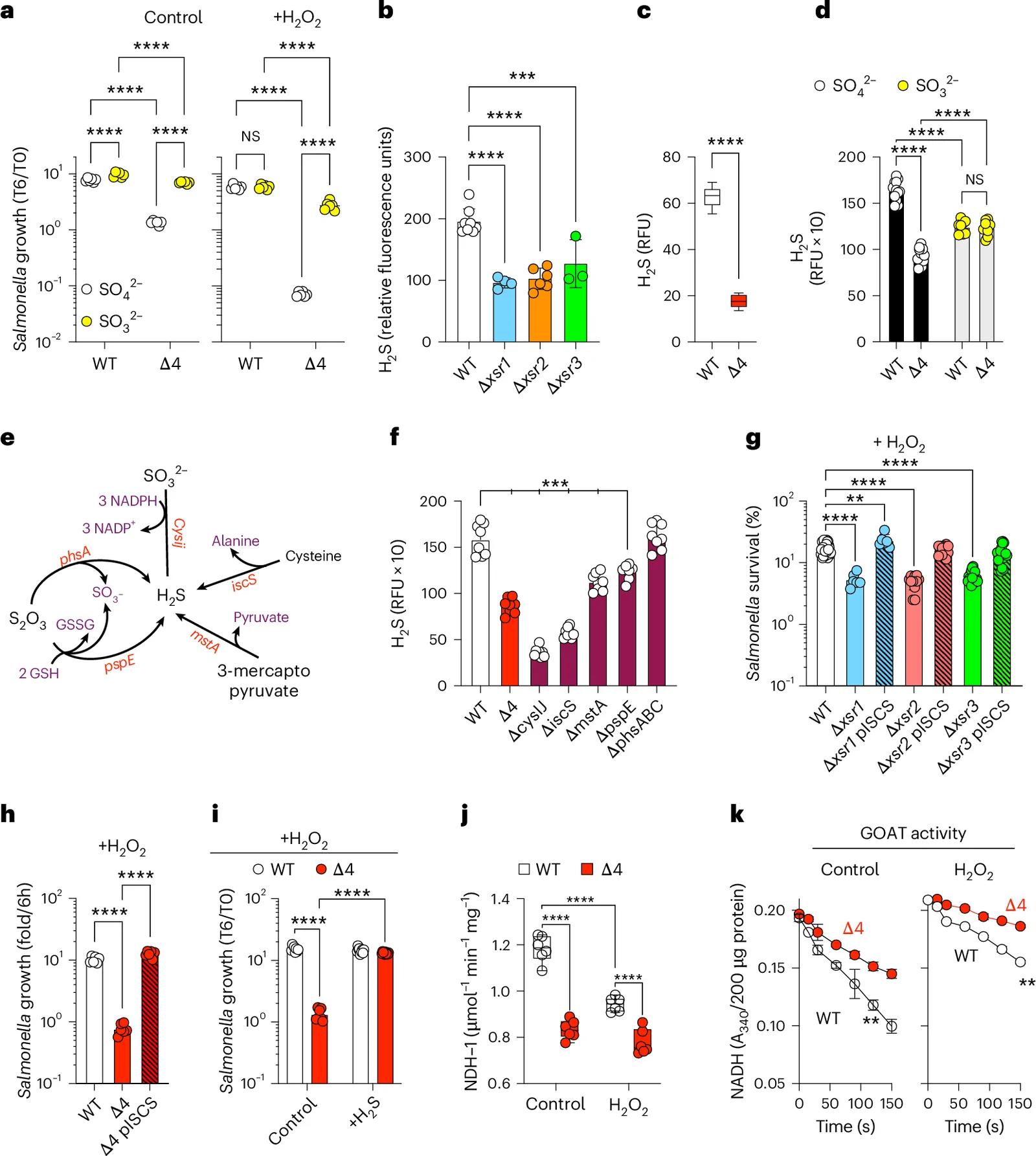
Extracytoplasmic sulfate reductases promote redox balance and resistance to oxidative stress in *Salmonella*. **a**, Susceptibility of *Salmonella* cultured in MOPS-GLC medium supplemented with either sulfate or sulfite was evaluated following treatment with 300 μM H_2_O_2_. Δ*4* is a quadruple mutant of all four *dmsA* paralogues. Bacterial survival was assessed by c.f.u. enumeration. Data are mean ± s.d.; *n* = 6 from 2 individual tests. **b**–**d**,**f**, Quantification of intracellular H_2_S concentrations in *Salmonella* grown in MOPS-GLC medium under ambient air with shaking (**b**,**c**,**f**) or standing (**d**). Culture media contained either sulfate (**b**–**d**,**f**) or sulfite (**d**) as the sole sulfur source. H_2_S was detected using the WSP-5 fluorescent (**b**,**c**) or 7-azido-4-methylcoumarin (**d**,**f**) probes. Relative fluorescence units (RFUs) were normalized to OD_600_. Data are mean ± s.d. of 2–4 independent experiments (**b**, *n* = 8, 6, 4, 3; **c**, *n* = 6; **d**, SO_4_^2−^ *n* = 12, SO_3_^3−^ *n* = 8; **f**, *n* = 8). (**c**, WT: mimima 55.441, maxima 69.114, centre 62.817; Δ*4*: mimima 13.563, maxima 21.340, centre 17.623). **e**, Sulfide production pathways in *Salmonella*. **g**,**h**, Survival of single (**g**) or quadruple (Δ*4*) (**h**,**i**) mutants after treatment with 200 (**g**) or 400 (**h**) μM H_2_O_2_ for 2 h in PBS (**g**) and 6 h in MOPS-GLC medium (**h**,**i**), respectively. Where indicated, the strains carried the pWSK29 plasmid expressing the *iscS* gene that encodes a cysteine desulfurase (pISCS). Data are mean ± s.d. of 3–10 independent experiments (**g**, *n* = 42, 18, 18, 6,6, 12, 12; **h**, *n* = 6). **i**, Effect of the addition of 100 μM sulfide (Na_2_S) on the growth of wild-type and Δ*4 Salmonella* in MOPS-GLC medium containing sulfate at 37 °C without shaking. Some cultures were treated with 100 μM H_2_O_2_. Data represent mean ± s.d. of 2–3 individual experiments (control *n* = 8; +H_2_S *n* = 10). Data points in **b**–**d** and **f**–**i** depict biological replicates. **j**, NDH-I dehydrogenase activity in inverted membranes isolated from *Salmonella* grown in MOPS-GLC-sulfate medium was assessed spectrophotometrically by monitoring the oxidation of deamino-NADH. Data are mean ± s.d.; *n* = 6 from 2 independent experiments (WT control: minima 1.099, maxima 1.243, centre 1.183; WT H_2_O_2_: minima 0.913, maxima 0.984, centre 0.943; Δ*4* control: minima 0.776, maxima 0.889, centre 0.834; Δ*4* H_2_O_2_: minima 0.732, maxima 0.864, centre 0.778) **k**, NADH-dependent GOGAT activity in *Salmonella* grown in MOPS-GLC-sulfate medium was measured spectrophotometrically by monitoring NADH oxidation at 340 nm. Data are presented as mean ± s.d.; *n* = 3 from 2 independent experiments. Some specimens were treated with 400 μM H_2_O_2_ for 30 min before assay (**j**,**k**). ^∗∗^*P* < 0.01, ^∗∗∗^*P* < 0.001, ^∗∗∗∗^*P* < 0.0001 by two-way ANOVA (**a**, d.f. = 1, 1, *F* = 73, 403; **d**, d.f. = 1, 1, *F* = 139, 150; **i**, d.f. = 1, 1, *F* = 23, 59), one-way ANOVA (**b**, d.f. = 3, *F* = 30; **f**, d.f. = 6, *F* = 167; **h**, d.f. = 2, *F* = 214), or two-tailed unpaired *t*-test (**c**, d.f. = 5,5, *F* = 2.2; **k**). Source data

**Extended Data Fig. 6:**
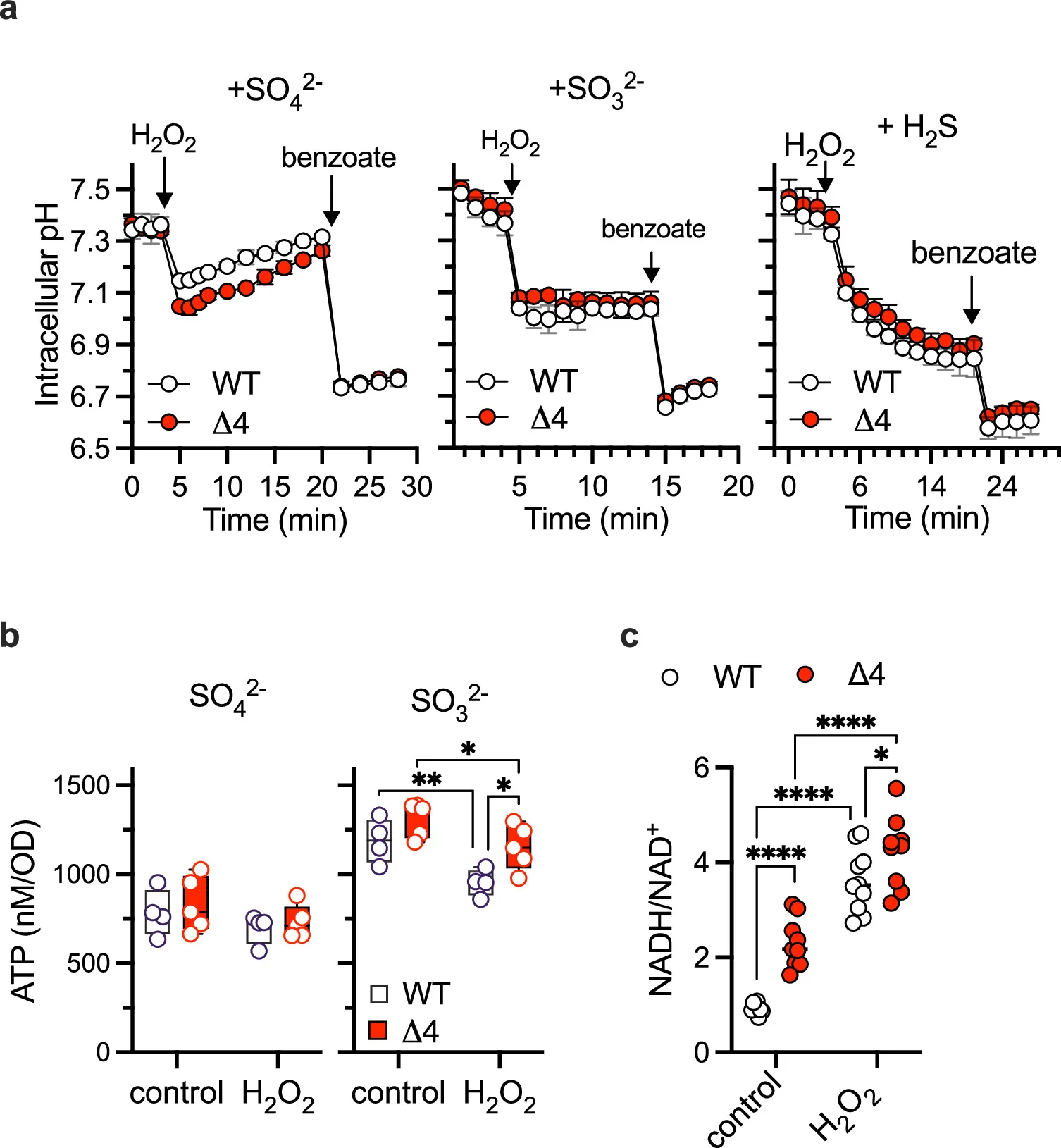
Effect of periplasmic sulfate reductases in the sulfate assimilatory pathway. **a**. Intracellular pH of exponential-phase *Salmonella* cultured in MOPS-GLC minimal medium plus either 250 μM sulfate, 250 μM sulfite, or 100 μM sulfide was measured using ratiometric pH-sensitive reporter pHluorin. Where indicated, the specimens were treated with 400 μM H_2_O_2_ or 40 mM of the protonophore benzoa.te. The data are mean ± SD; n = 3 from 3 independent experiments. **b**. Intracellular ATP levels were measured using a luciferase-based assay from *Salmonella* grown in MOPS-GLC minimal medium supplemented with either sulfate or sulfite. Data represent the mean ± SD from 2 individual experiments (WT n = 4, Δ4 n = 5). (sulfate WT control; minima-634.697, maxima-954.162, centre-831.757, sulfate WT H_2_O_2_; minima-568.906, maxima-754.559, centre-695.414, sulfate Δ*4* control; minima-666.640, maxima-1027.286, centre-831.757, sulfate Δ*4* H_2_O_2_; minima-657.745, maxima-880.605, centre-733.134, sulfite WT control; minima-1041.930, maxima-1333.197, centre-1189.822, sulfite WT H_2_O_2_; minima-859.320, maxima-1041.895, centre-953.781, sulfite Δ*4* control; minima-1181.106, maxima-1387.895, centre-1210.322, sulfite Δ*4* H_2_O_2_; minima-979.384, maxima-1296.313, centre-1151.357) **c**. Intracellular NADH / NAD^+^ ratios were quantified in *Salmonella* grown aerobically in MOPS-GLC minimal medium supplemented with sulfate. The data are the mean ± SD from 2 individual experiments (WT control n = 8; WT H_2_O_2_ n = 10; Δ*4* n = 9). *, **, **** *P* < 0.1, 0.01, 0.0001, respectively, as estimated by one-way ANOVA (**b, c**). Source data

**Extended Data Fig. 7:**
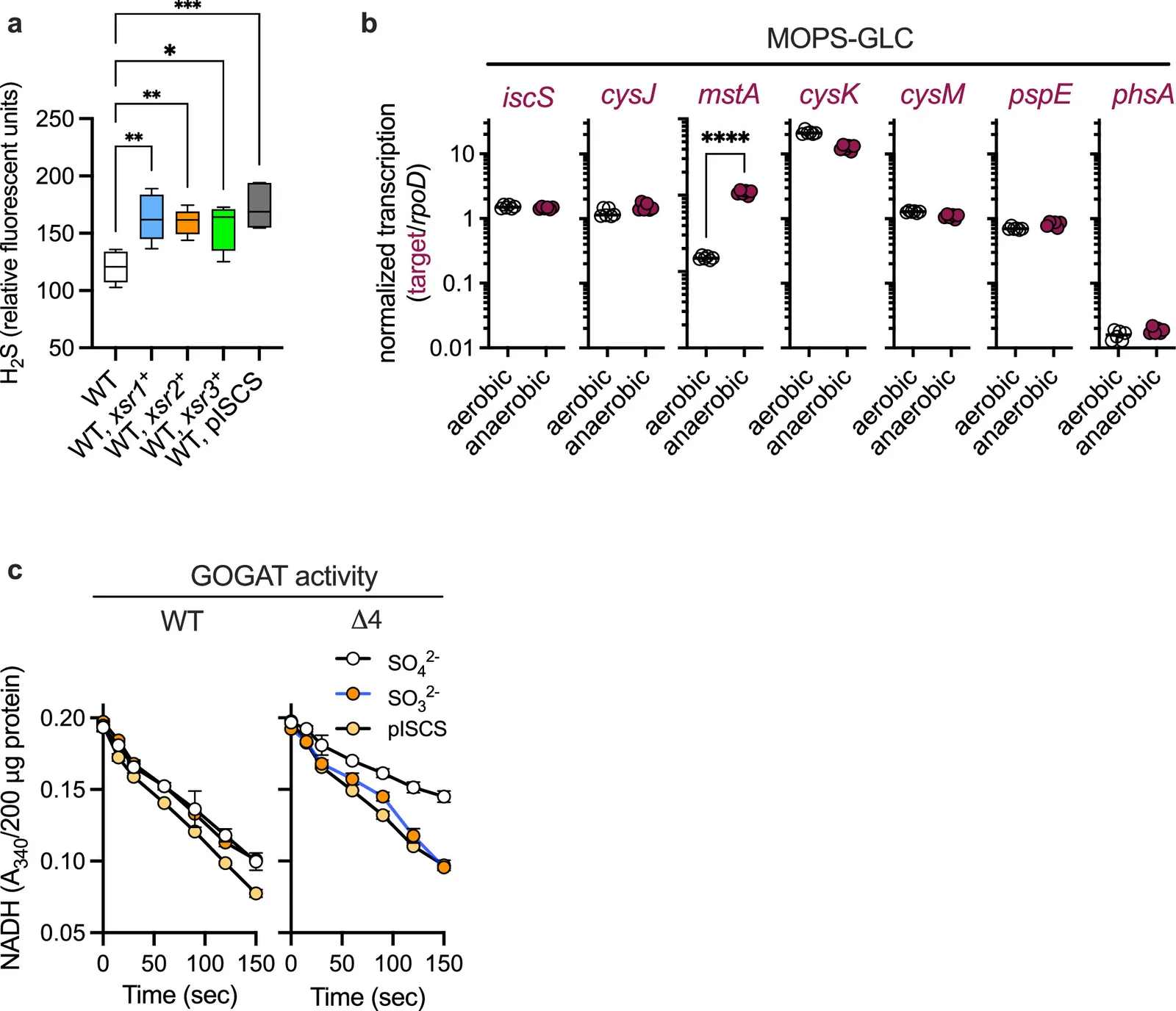
Periplasmic sulfate reductases are involved in the assimilation of sulfur. **a**, Hydrogen sulfide (H_2_S) production was evaluated in wild-type *Salmonella* harboring each individual periplasmic sulfate reductase operon (*xsr1*, *xsr2* and *xsr3*) or the *iscS* gene in pWSK29. Cells were cultured in MOPS-GLC medium containing sulfate. H_2_S was detected using the WSP5 fluorescent probe. The data are the mean ± SD from 2-3 independent experiments (n = 6, WT xsr1^+^ n = 4). (WT; minima-102.772, maxima-135.988, centre-120.390, WT xsr1^+^; minima-136.495, maxima-181.866, centre-163.182, WT xsr2^+^; minima-143.805, maxima-174.574, centre-159.986, WT xsr3^+^; minima-125.332, maxima-165.419, centre-156.655, WT pISCS; minima-154.549, maxima-194.787, centre-172.543) **b**. Expression of indicated genes was quantified by real-time qRT-PCR using RNA isolated from wild-type *Salmonella* cultured aerobically or anaerobically in MOPS-GLC medium supplemented with sulfate. The data are mean ± SD; n = 6 from 2 individual experiments. *, **, ***, **** *P* < 0.1, 0.01, 0.001, 0.0001, respectively, as estimated by one-way ANOVA (**a**), or unpaired *t*-test (**b**). **c**. Glutamate synthase (GOGAT) activity was assessed kinetically by measuring the oxidation of NADH. Specimens were prepared from *Salmonella* grown in MOPS-GLC minimal medium containing either sulfate or sulfite. Selected strains harbored the *iscS* gene from the pWSK29 plasmid. The data are mean ± SD; n = 3 from 2 independent experiments. Symbols in a and c depict biological replicates. Source data

We next examined how extracytoplasmic sulfate reduction promotes *Salmonella* growth and antioxidant defence. Many members of the MopB superfamily couple substrate reduction with energetics. Therefore, we first tested the contribution of Xsr enzymatic complexes to ATP production and ΔpH maintenance. Wild-type and Δ4 strains maintained similar cytoplasmic pH and ATP levels under resting and H_2_O_2_ stress (Extended Data Fig. 6a,b), indicating that these enzymes do not generate ATP via dissimilatory sulfate reduction. The activity of the Xsr enzymatic complexes nonetheless helped *Salmonella* maintain redox balance (Extended Data Fig. 6c). In the assimilatory pathway, sulfate is reduced to hydrogen sulfide (H_2_S) via a sulfite intermediate. H_2_O_2_ had a more profound bacteriostatic effect on the Δ4 *Salmonella* strain than on wild-type controls (Fig. 5a). The addition of sulfite completely rescued the growth defect in untreated or H_2_O_2_-treated Δ4 *Salmonella* (Fig. 5a). Together, these investigations suggest that the synthesis of sulfite enabled by extracytoplasmic sulfate reductases promotes growth and antioxidant defence. Strains of *Salmonella* lacking individual *xsr1*, *xsr2* or *xsr3* gene clusters synthesized reduced levels of H_2_S compared to wild-type controls (Fig. 5b), a defect that was accentuated in the Δ4 strain lacking all extracytoplasmic sulfate reductases (Fig. 5c). Expression of the *xsr1*, *xsr2* or *xsr3* gene clusters from the low-copy pWSK29 plasmid enhanced the H_2_S-producing capacity of wild-type *Salmonella* (Extended Data Fig. 7a). The defective production of H_2_S by the Δ4 *Salmonella* strain is a consequence of its lack of sulfite production, as suggested by the restoration of intracellular H_2_S synthesis upon the addition of sulfite (Fig. 5d). The *Salmonella* genome encodes several H_2_S-generating pathways (Fig. 5e), one of which, *mstA*, is activated under anaerobiosis (Extended Data Fig. 7b). The production of H_2_S by anaerobic *Salmonella* using sulfate as the sole sulfur donor is mediated, in decreasing order of importance, by *cysIJ*, *iscS*, *mstA* and *pspE* gene products (Fig. 5f). As could be expected, in the absence of thiosulfate, the PhsABC complex did not contribute to H_2_S synthesis in MOPS-GLC minimum medium (Fig. 5f). It is likely that the H_2_S synthesized by the Xsr-dependent assimilation of sulfate requires the reduction of sulfite by CysIJ. In support of the idea that Xsr proteins help *Salmonella* by enabling H_2_S production, the expression of *iscS-*encoded cysteine desulfurase (a major source of H_2_S) *in trans* or supplementation of the culture media with an H_2_S donor restored the growth and peroxide resistance of individual *Δxsr* strains and the Δ*4 Salmonella* mutant (Fig. 5g–i).

H_2_S indirectly promotes [Fe–S] cluster biogenesis by enabling synthesis of cysteine, which is the substrate of the desulfurases SufA and IscA^21,22^. Therefore, we compared the activity of the *nuo*-encoded NADH dehydrogenase that harbours 9 [Fe–S], including 7[4Fe–4S] clusters, between wild-type and Δ*4 Salmonella*^23^. The Δ4 *Salmonella* strain contained lower *nuo*-encoded NADH dehydrogenase activity than wild-type controls as assessed by the reduction of deamino-NADH (Fig. 5j). The Δ4 *Salmonella* strain also harboured lower levels of the [4Fe–4S]-containing glutamate synthase (Fig. 5k and Extended Data Fig. 7c). By fostering glutamate synthase and NDH-I enzymatic activities, the assimilation of sulfur by the enzymatic activity of extracytoplasmic sulfate reductases probably contributes to the growth and oxidative stress resistance of *Salmonella*^24–26^.

### DmsA paralogues are widely represented in the MopB lineage

**Fig. 6:**
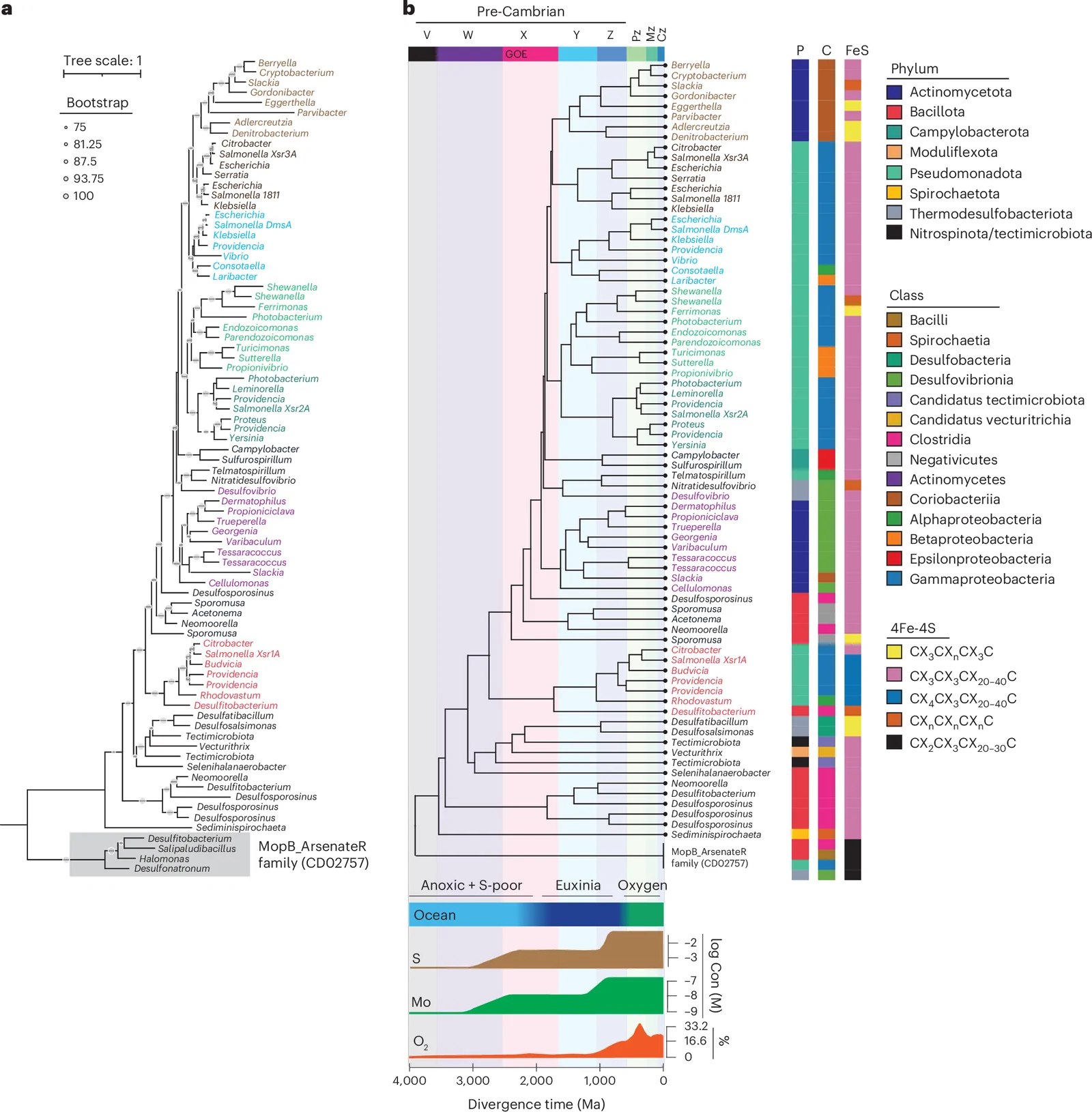
Global phylogeny and temporal diversification of DmsA_EC catalytic subunits. **a**, Maximum-likelihood phylogeny of the DmsA_EC clade within the MopB superfamily. The tree was inferred from a curated 80-sequence alignment comprising 60 non-redundant representatives of the MopB_DmsA_EC conserved-domain family (CD02770), the 5 *Salmonella* Typhimurium 14028s DmsA/XsrA paralogues, 15 reference enterobacterial homologues and 4 arsenate reductase outgroup sequences. Branch lengths are drawn to scale and were estimated under the LG + F + R6 model. The tree is rooted on the arsenate reductase bipartition. All bacterial and archaeal lineages are colour coded and annotated at their respective crowns. **b**, Time-calibrated molecular timetree of DmsA_EC catalytic subunits and their emergence in Earth history. Divergence times were estimated from Fig. 6a topology using a RelTime-ML framework under the LG + Γ+F model, with soft calibration bounds derived from well-supported bacterial sister pairs and published geochemical constraints. Coloured time bars denoting approximate positions of key geobiological transitions (for example, Neo-Archaean sulfur/molybdenum influx, Paleoproterozoic GOE) are indicated schematically along the time axis to illustrate the temporal coincidence between redox evolution and the major DmsA_EC radiations. V, Hadean (4540–4000 Mya); W, Archean (4000–2500 Mya), X, Paleoproterozoic (2500–1600 Mya); Y, Mesoproterozoic (1600–1000 Mya); Z, Neoproterozoic (1000–538.8 Mya); Pz, Paleozoic (541–252 Mya); Mz, Mesozoic (252–66 Mya); Cz, Cenozoic (66 Mya); P, phyla; C, class; FeS, iron sulfur.

**Extended Data Fig. 8:**
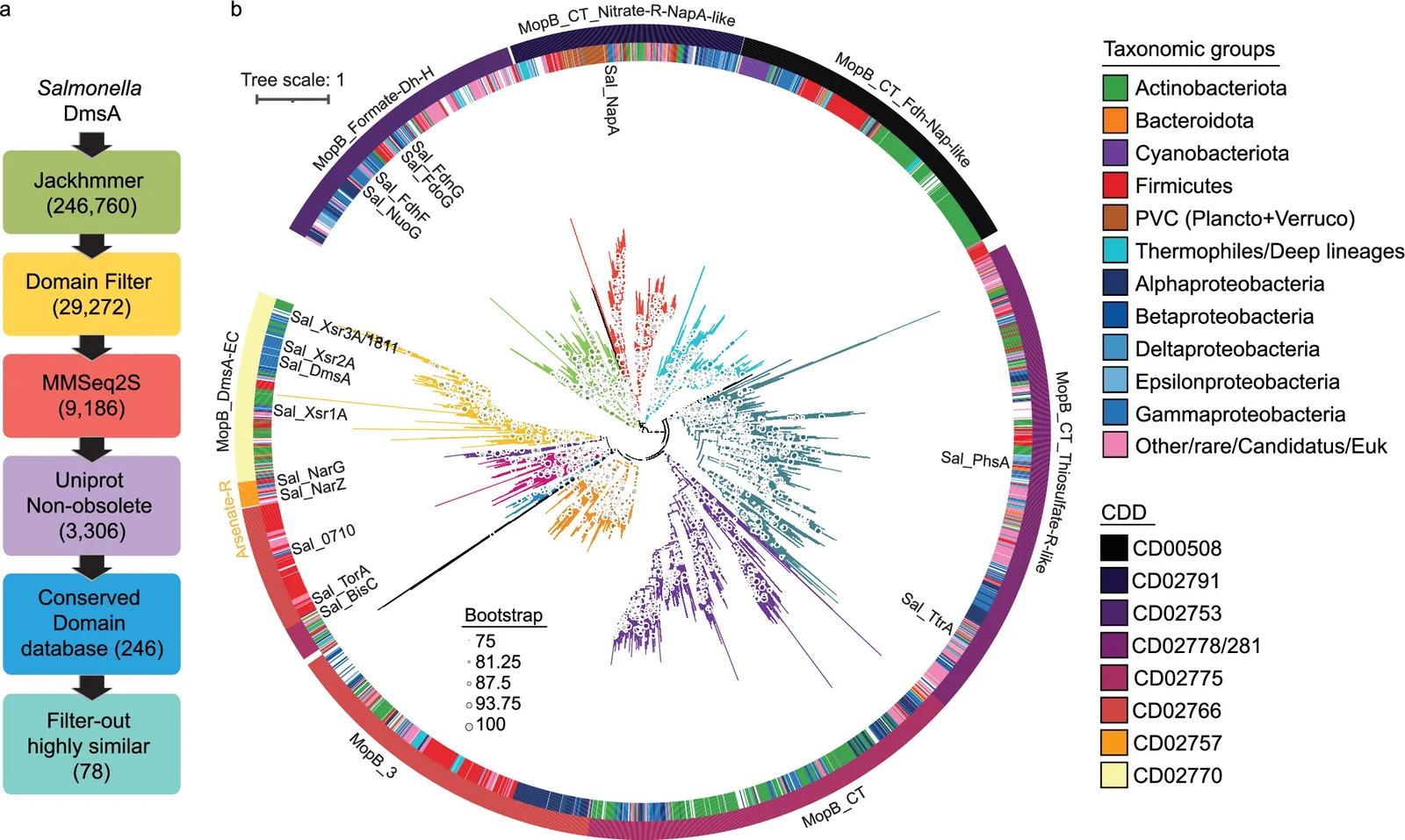
Phylogeny of MopB domain– and 4Fe–4S-containing proteins constructed using 1,000 ultrafast bootstrap approximations. **a**. Workflow used to recover sequences highly similar to the *Salmonella* DmsA catalytic subunit, integrating JackHMMER searches, conserved-domain filtering, and MMseqs2 clustering; only non‑obsolete UniProt entries were retained at the end (sequence counts and details per step are reported in Supplementary Tables SX). **b**. Maximum-likelihood tree of 3,307 MopB-domain hits plus 17 MopB homologs from *Salmonella enterica* serovar Typhimurium 14028 s. Taxonomic affiliations and conserved domain (CDD) assignments are indicated as colored rings. The tree is rooted on the MopB_Formate-Dh-H CD family (CD02753). Source data

**Extended Data Fig. 9:**
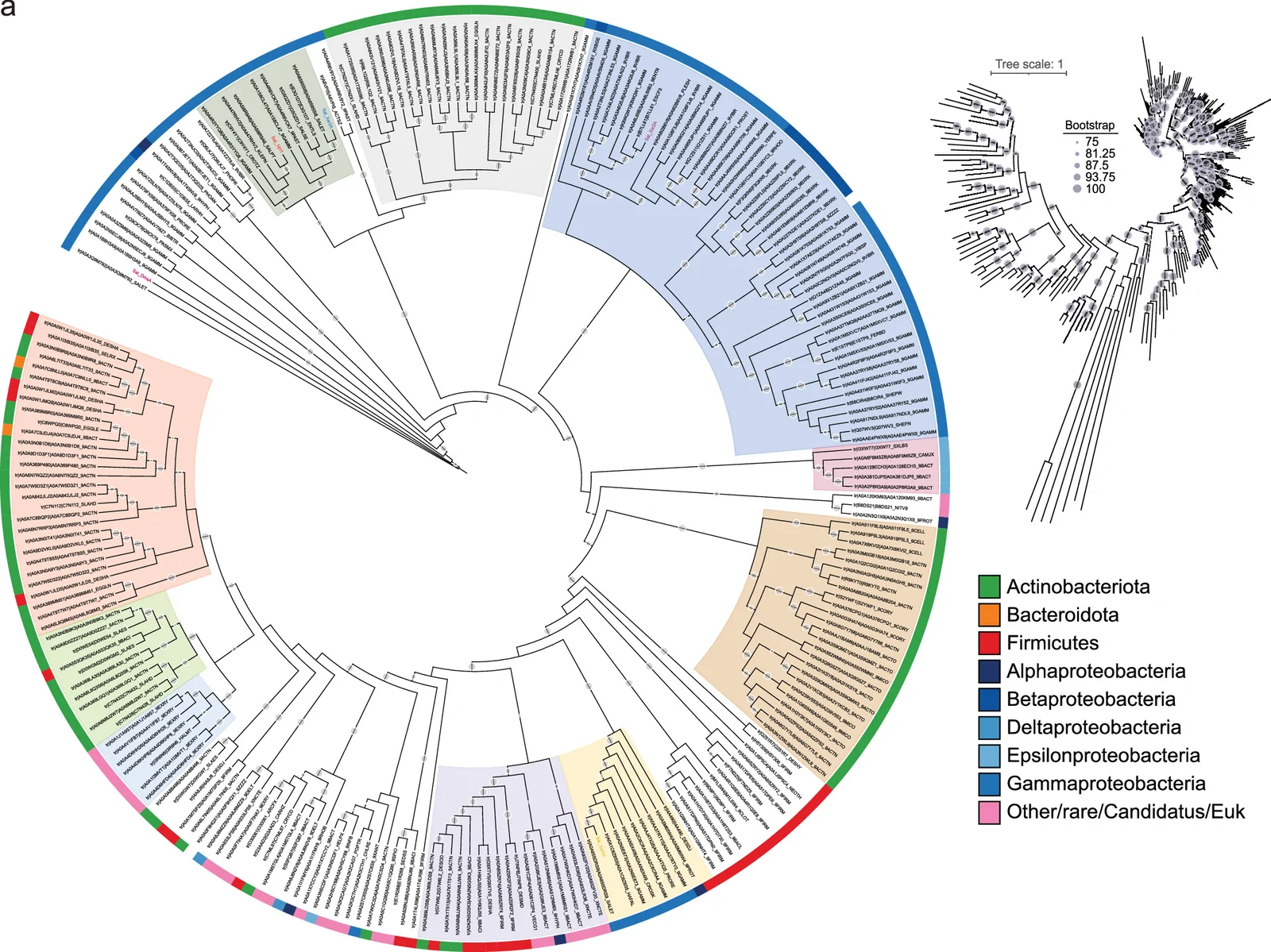
Phylogeny of DmsA-EC (CD02770) proteins constructed using 1,000 ultrafast bootstrap approximations. **a**. Workflow used to recover sequences highly similar to the *Salmonella* DmsA catalytic subunit, integrating JackHMMER searches, conserved-domain filtering, and MMseqs2 clustering; only non‑obsolete UniProt entries were retained at the end (sequence counts and details per step are reported in Supplementary Tables Table SX). **b**. Maximum-likelihood tree of 3,307 MopB-domain hits plus 17 MopB homologs from *Salmonella enterica* serovar Typhimurium 14028 s. Taxonomic affiliations and conserved domain (CDD) assignments are indicated as colored rings. The tree is rooted on the MopB_Formate-Dh-H CD family (CD02753). Source data

**Extended Data Fig. 10:**
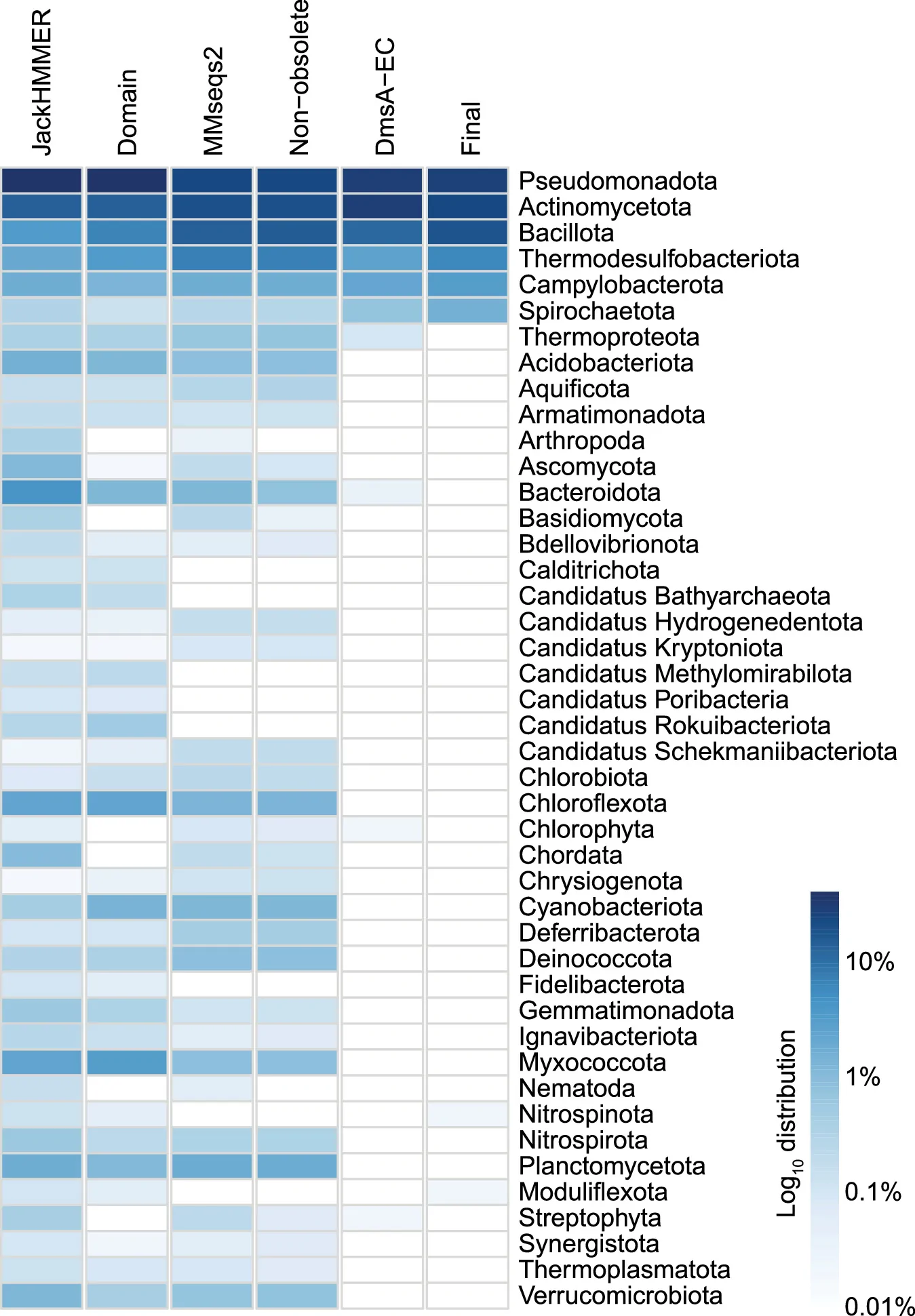
Phylum-level taxonomic distribution of MopB_DmsA_EC homologs across sequential filtering steps. Heatmap displaying the percentage representation of each bacterial and archaeal phylum at each filtering step, with color intensity reflecting the proportion of sequences assigned to that phylum within the respective step on a log_10_ scale. Phyla contributing less than 0.1% of sequences in all filtering steps are excluded for clarity. Homologs of DmsA in other MopB families were present in almost all listed phyla during filtering and curation. In the final two steps, only the DmsA_EC proteins were retained. Apparent absences in specific lineages may reflect gaps in sequencing or database coverage rather than true biological absence. Source data

Our finding that extracytoplasmic sulfate reductases retain canonical MopB architecture yet function independently of the MOCO cofactor raises the question of whether this catalytic remodelling is a lineage-specific innovation in *Salmonella* or reflects a broader evolutionary flexibility within the MopB superfamily. To address this dichotomy, we placed the *Salmonella* DmsA and Xsr paralogues within a broader phylogenetic framework of 3,324 MopB catalytic subunits (Extended Data Fig. 8 and Supplementary Data 1). Within this phylogeny, sequences carrying the Mop_DmsA_EC (DmsA-like extracytoplasmic catalytic subunits) conserved-domain signature (CD02770) formed a single well-supported monophyletic group, branching from MopB_Arsenate-R family proteins (CD02757). DmsA_EC homologues were detected across diverse bacterial and archaeal phyla, encompassing sequences from sediment, environmental and host-associated organisms (Extended Data Figs. 9 and 10). These findings indicate that the DmsA-like architecture is not restricted to Proteobacteria. For a focused phylogenetic analysis, 80 representative proteins were used to infer a maximum-likelihood phylogeny (LG + F + R6; 1,000 UFBoot). An unrooted phylogeny of this subset resolved major, well-separated clades corresponding to DmsA, Xsr1A, Xsr2A, Xsr3A, Coriobacteriia-associated sequences, Actinomycetota and Bacillota-associated sequences, indicating substantial divergence within the DmsA_EC family. To infer branching direction, the tree was rooted using bacterial sequences from the MopB_Arsenate-R family, consistent with previous studies that placed bacterial arsenate reductase-like sequences near the base of MopB family^7^ (Fig. 6a and Supplementry Data 1).

DmsA_EC homologues from Bacillota formed a basal clade to the arsenate reductase outgroup in the rooted topology, branching proximal to the root and forming a sister clade to all remaining bacterial DmsA_EC sequences. Xsr1A homologues formed a distinct cluster, with an Actinomycetota-associated lineage occupying a position closer to the root relative to the remaining Xsr and DmsA homologues. Xsr2A, Xsr3A and DmsA homologues formed well-supported distinct clusters further from the root, indicating that these paralogues share a more recent common ancestor relative to the Actinomycetota-associated lineage. Within the Gammaproteobacteria, DmsA_EC homologues encompassed both environmental (*Ferrimonas* and *Shewanella*) and enteric taxa (*Providencia, Budvicia* and *Citrobacter*) in a paraphyletic clade, with *Salmonella* paralogues partitioned across different lineages (Fig. 6a). *Salmonella* Xsr1A grouped with homologues from *Providencia*, *Budvicia* and *Citrobacter*, while Xsr2A associated with a *Photobacterium*/*Providencia* subgroup. In contrast, *Salmonella* DmsA, Xsr3A and STM14_1811 were grouped within a well-supported Enterobacterales cluster alongside homologues from *E. coli*, *Serratia* and *Klebsiella* (bootstrap 100%). Notably, the Xsr3A subclade is sister to a fully monophyletic *Coriobacteriia* group, with short internal branches and strong nodal support at this bipartition, indicating a shared evolutionary history of DmsA–EC-like proteins between enteric Gammaproteobacteria and Gram‑positive gut specialists. Collectively, the wide phylogenetic distribution of *Salmonella* DmsA_EC paralogues in different lineages of the maximum-likelihood (ML) tree suggests that the diversification of XsrA paralogues occurred progressively within the broader DmsA_EC subfamily.

Dating analysis with soft calibration bounds placed DmsA_EC radiation in a temporal context. While single-gene molecular dating precludes precise temporal assignments, the RelTime-ML analysis suggested that the split between the arsenate reductase outgroup and the Bacillota DmsA_EC clade predated the early Proterozoic, with subsequent Firmicute diversification of Actinomycetota and Gammaproteobacterial lineages occurring progressively thereafter (Fig. 6b). While Gammaproteobacterial radiations that include the basal Xsr1A and Xsr2A branches fell within the Neoproterozoic–early Palaeozoic, the Enterobacterales–Coriobacteriia assemblage containing *Salmonella* DmsA/Xsr3 mapped to the late Paleozoic–Mesozoic. The relative divergence estimates are broadly consistent with major redox transitions reconstructed for Earth history. Strikingly, inspection of N-terminal [4Fe–4S] coordination motifs across the 80 proteins in the tree revealed five distinct cysteine spacing classes distributed randomly across lineages (Fig. 6b). Xsr3A and DmsA share the same [Fe–S] cysteine spacing class but differ in catalytic mechanism, indicating that functional divergence within the DmsA_EC family does not necessarily require changes in [Fe–S] cofactor coordination geometry. Collectively, these observations suggest that MOCO-independent [Fe–S]-dependent catalysis is not a lineage-specific innovation confined to *Salmonella* but reflects a broader evolutionary flexibility within the DmsA_EC clade of the MopB superfamily.

## Discussion

Terminal respiratory enzymes of the MopB superfamily help with the energetics of bacterial cells. Our investigations demonstrate that extracytoplasmic sulfate reductases of the MopB superfamily enhance *Salmonella*’s capacity to colonize the gut and establish a stronghold in viscera (Supplementary Fig. 2). Rather than using sulfate as a terminal acceptor for energy conservation, extracytoplasmic sulfate reductases facilitate sulfate assimilation. The expression of three redundant extracytoplasmic sulfate reductases may help *Salmonella* compete with resident microbiota and the host for the millimolar concentrations of this oxyanion^27^. In addition, the H_2_S produced during sulfate reduction can benefit *Salmonella* by promoting metabolism, antagonizing oxidative products of the phagocyte NADPH oxidase (herein), or inhibiting resident microbiota^8^. Our current investigations add nuances to the recent realization that *Salmonella* leverages several members of the MopB superfamily, including DmsABC, PhsABC and XsrABC, to generate bioactive H_2_S (refs. ^6,8^). The diversity of MopB enzymatic complexes offers *Salmonella* alternative conduits for the exploitation of a wide array of sulfur-based terminal electron acceptors in gut and viscera.

In addition to the extracytoplasmic sulfate reductases described herein, non-typhoidal *Salmonella* uses the cytoplasmic sulfate reductase CysIJ that catalyses the reaction of sulfate with ATP to form the adenosine-5’-phosphosulfate intermediate. The assimilatory sulfate-reducing cytoplasmic pathway is preserved in *S*. Typhi and *S*. Paratyphi. However, these human-adapted *Salmonella* serovars have accrued inactivating mutations in all three extracytoplasmic sulfate reductases^28^. Differences in the sulfate assimilatory pathways between typhoidal and non-typhoidal serovars may reflect disparities in their lifestyles. Whereas non-typhoidal *Salmonella* elicits oxidative stress from neutrophils and macrophages, the capsule and very long O-antigen in Typhi and Paratyphi limit the assembly of phagocyte NADPH oxidase enzymatic complexes^29^. ROS generated during the respiratory burst may impose greater demands on sulfate assimilation in non-typhoidal *Salmonella* to enhance metabolic endurance, thereby providing strong selective pressures to expand sulfate reductase capabilities in non-typhoidal *Salmonella*. Auxotrophies for cysteine^30^, therefore imposing lower demands for H_2_S in cysteine biosynthesis, may have also contributed to the pseudogenization of *dmsA* paralogues in typhoidal *Salmonella* serovars.

The synthesis of O_2_ by photosynthetic cyanobacteria 2.5 billion years ago brought about the GOE, a period in Earth’s history of profound geochemical changes and a formidable force of evolution, as illustrated by the Archaea and bacterial symbiogenesis that launched Eukaryota^31^. In addition to fomenting ATP synthesis in aerobic respiration, O_2_ enabled the synthesis of diverse terminal electron acceptors. Microorganisms exploited the versatility of the molybdenum-dependent DMSO reductase superfamily to exploit the newly formed terminal electron acceptors^7^. Genes of the DMSO reductase superfamily account for 2.2% of the *Salmonella* genome and power cellular energetics via the reduction of formate, nitrate, DMSO, methionine sulfoxide, tetrathionate, thiosulfate and trimethylamine *N*-oxide. We have discovered that three of the *dmsA* paralogues encode previously unknown assimilatory extracytoplasmic sulfate reductases that support gastrointestinal colonization while also fostering oxidative stress resistance in non-typhoidal *Salmonella*.

The electrochemistry catalysed by DMSO reductase family members spans more than 1 volt, allowing organisms to leverage a wide variety of redox substrates^32^. The rich chemistry catalysed by these reductases is founded on two-electron reactions in which molybdenum cycles between the IV and VI redox states. The catalytic versatility of molybdenum is dictated by the redox environment provided by coordinating pyranopterin dithiolenes and the side chains of neighbouring amino acids^16^. Extracytoplasmic sulfate reductases retain the structural fold and bear the conserved coordinating serine residue of canonical DmsA. Despite these conserved features, the catalytic cycle of *Salmonella* extracytoplasmic sulfate reductases does not involve the MOCO cofactor, as demonstrated by the excellent sulfate reductase activity of variants bearing mutations in the coordinating serine residue and MOCO-deficient Δmoa *Salmonella* strains. Instead, sulfate reduction by these extracytoplasmic proteins involves the [Fe–S] metal centre, as shown by the sulfate-induced changes in EPR spectra of the Xsr3 complex. Moreover, Asn^66^ and bridge Arg^105^, located near Cys^A^ and Cys^B^ residues coordinating the [4Fe–4S] cluster, are vital for sulfate reductase activity. The MOCO-independent function of extracytoplasmic sulfate reductases contrasts with the [Fe–S]-independent functions of the DorA and TorA branch of the MopB superfamily^7^. DmsA homologues with molybdenum-independent sulfate reduction activity were grouped with some distant *dmsA*-family lineages in our phylogenetic analysis, which could share some enzymology.

## Methods

### Bacterial strains, plasmids and growth conditions

*Salmonella enterica* serovar Typhimurium strain 14028s and its derivatives, along with *Escherichia coli* strains and the plasmids used in this study, are listed in Supplementary Tables. Deletion mutants were constructed using the λ-Red homologous recombination system as previously described^33^. Site-directed mutagenesis was performed using Pfu Ultra High-Fidelity DNA Polymerase (Agilent) following manufacturer instructions. The primers used in this study are described in Supplementary Tables. Bacteria were routinely grown in LB broth at 37 °C with shaking in ambient air. Where indicated, *Salmonella* was grown in morpholinopropanesulfonic acid (MOPS) minimal medium (40 mM MOPS, 1 mM tricine, 2 mM K_2_HPO_4_, 10 μM FeSO_4_, 9.5 mM NH_4_Cl, 276 μM K_2_SO_4_, 0.5 μM CaCl_2_, 50 mM NaCl, 525 µM MgCl_2_; pH 7.2) supplemented with 0.4% casamino acid (MOPS-CAA) or 0.4% D-glucose (MOPS-glucose [GLC])^34^. Where specified, alternative sulfur sources (Na_2_SO_3_, Na_2_S_2_O_3_, or Na_2_S) were used instead of K_2_SO_4_ to prepare MOPS-sulfite, MOPS-thiosulfate or MOPS-sulfide media, respectively. Bacteria were grown under aerobic conditions in a shaking incubator, anaerobic conditions in a Bactron anaerobic chamber (Shel Lab, Cornelius, OR), or hypoxic conditions in tightly closed tubes filled with medium and incubated standing. When appropriate, penicillin, chloramphenicol, tetracycline and kanamycin were added at final concentrations of 250, 40, 20 or 50 μg ml^−1^, respectively.

### Animal studies

All mice were bred according to protocol number 00059 approved for 3 years on 29/04/2025 by the Institutional Animal Care and Use Committee (IACUC) at the University of Colorado School of Medicine. Male and female mice were assigned randomly to the experimental groups. C57BL/6 mice (6–8-week-old) were inoculated i.p. with ~300 colony-forming units (c.f.u.s) of *Salmonella* mixtures containing equal numbers of wild-type and mutant strains. Bacterial burdens were quantified in livers and spleens at 3 days post-infection by plating onto LB agar containing the appropriate antibiotics. Competitive index was calculated as (strain 1/strain 2) output / (strain 1/strain 2) input. For oral infection, a streptomycin-pretreatment colitis model was used as previously described^35^. Briefly, 6–8-week-old C57BL/6 and congenic Cybb^−/−^ mice were deprived of food and water for 4 h before treatment with 20 mg of streptomycin by oral gavage. After 24 h of streptomycin treatment, mice were inoculated orally with 100 μl *Salmonella* mixture containing 3.5 × 10^8^ c.f.u.s of equal numbers of wild-type and mutant strains using gavage needles. Where indicated, some C57BL/6 mice that did not receive streptomycin were gavaged orally with 3.5 × 10^8^ c.f.u.s of *Salmonella* mixtures. The bacterial burden was assessed by enumerating c.f.u.s from the caecum, ileum, colon, mesenteric lymph nodes (MLN), liver and spleen at 4 days post-infection.

### Macrophage killing assays

Peritoneal macrophages were isolated from 6–8-week-old C57BL/6 and *Cybb*^−/−^ mice at 4 days after intraperitoneal injection with 1 ml of filter-sterilized sodium periodate (1 mg ml^−1^ in PBS; Millipore Sigma). Macrophages were collected in RPMI^+^ medium (RPMI medium supplemented with 10% heat-inactivated fetal bovine serum, 1 mM sodium pyruvate, 2 mM L-glutamine and 20 mM HEPES). Peritoneal exudate cells were collected by centrifugation at 500 *g* for 10 min and resuspended in RPMI^+^ medium at 3 × 10^6^ cells per ml. Of the peritoneal exudate cells, 100 μl were seeded per well in 96-well plates and incubated at 37 °C in a 5% CO_2_ tissue culture chamber. Confluent peritoneal macrophages were infected at a multiplicity of infection of 2 with *Salmonella* grown overnight in LB broth at 37 °C in a hypoxia chamber (Coy Laboratory Products) containing 1% O_2_. After 1 h of infection, cells were incubated for an additional hour with fresh RPMI^+^ medium containing 50 μg ml^−1^ gentamicin to eliminate extracellular bacteria. Medium was then replaced with RPMI^+^ medium containing 10 μg ml^−1^ gentamicin. Specimens were lysed in PBS with 0.1% Triton X-100 after 0, 2 and 20 h of culture in a hypoxic chamber. Lysates were serially diluted in PBS and plated on LB agar. C.f.u.s were enumerated after overnight incubation at 37 °C. Fold replication of intracellular *Salmonella* was calculated as c.f.u.s at 2 h and 20 h post-infection relative to the burden recovered at time zero.

### H_2_O_2_ killing assays

*Salmonella* grown overnight in LB broth at 37 °C in a shaker incubator was diluted to 2 × 10^6^ c.f.u.s/200 μl in PBS and seeded in 96-well plates. The specimens were challenged for 2 h at 37 °C with 400 μM of H_2_O_2_. Percent survival was calculated as c.f.u.s of the H_2_O_2_-treated / c.f.u.s of untreated samples. Where indicated, *Salmonella* grown aerobically overnight in LB broth at 37 °C were diluted to ~5 × 10^7^ cells per ml in MOPS-GLC minimum medium. Bacterial cultures were exposed to 100–300 μM H_2_O_2_ under either shaking or hypoxic conditions. At the designated time points, samples were serially diluted and plated on LB agar for c.f.u. enumeration. Bacterial survival was assessed by calculating the fold change in c.f.u.s relative to time zero.

### Anaerobic growth assay

*Salmonella* grown aerobically overnight in LB broth at 37 °C were diluted to ~5 × 10^7^ cells per ml in either MOPS-CAA minimal medium or LB broth. Cells were transferred to a Bactron anaerobic chamber (Shel Lab) and treated with 40 mM of the terminal electron acceptors DMSO or L-methionine sulfoxide (both from Millipore Sigma). Cell growth was monitored by measuring optical density at 600 nm (OD_600_), or by c.f.u. enumeration. Fold growth was calculated as the ratio of c.f.u.s recovered at 24 h versus time zero.

### Isolation of DmsABC::6His or Xsr3ABC::6His protein complexes

The *dmsABC* or *xsr3ABC* constructs cloned in the low-copy plasmid pWSK29 (Supplementary Tables) were engineered to express a 6His tag at the C terminus of the C subunit by site-directed mutagenesis using Pfu Ultra High-Fidelity DNA Polymerase (Agilent). Where indicated, a glycine mutation was engineered into the molybdenum-coordinating serine residue of the A subunit. The Δ5 *Salmonella* strain expressing pWSK29 plasmids carrying the 6His-tagged *dmsABC* or *xsr3ABC* constructs was anaerobically cultured overnight in LB broth in an anaerobic chamber (Shel Lab). Bacterial cells were collected by centrifugation at 8,000 *g* for 5 min and disrupted by sonication using a bulk tip at 60% amplitude using alternating 30-s pulses for a total of 3 min. Cell-free extracts were obtained after 30 min centrifugation at 10,000 *g*. 6His-tagged fusion proteins were purified according to manufacturer protocols using TALON metal-affinity chromatography (Clontech). To ensure isolation of A and B subunits along with the 6His-tagged C subunit, the salt concentration was reduced to 50 mM Tris-HCl pH 8.0 buffer. The purity and mass of the affinity-purified ABC complexes were assessed in SDS–PAGE gels. Protein concentration was measured using the bicinchoninic acid (BCA) assay.

### Membrane preparation

The membranes for DMSO or sulfate reductase activity assays and EPR spectroscopy were isolated as previously described^6^. Briefly, *Salmonella* cells grown overnight anaerobically in LB broth in an anaerobic chamber (Shel Lab) were collected by centrifugation at 8,000 *g* for 5 min and resuspended in 3 ml of 50 mM Tris-HCl buffer pH 8.0 containing 0.1 mg ml^−1^ RNase (Millipore Sigma), 0.1 mg ml^−1^ DNase (Promega) and 2 mM phenylmethylsulfonyl fluoride (PMSF). Bacterial cultures were disrupted on ice using a probe sonicator (Sonic Dismembrator model 100, Thermo Fisher) at 40% amplitude with alternating 5- and 10-s pulses for a total sonication time of 30 s. Crude cellular debris was removed by centrifugation at 10,000 *g* for 15 min at 4 °C. The resulting supernatant was ultracentrifuged at 150,000 *g* for 1.5 h at 4 °C in an Optima XL-100K ultracentrifuge (Beckman). The membrane-containing pellets were suspended in 20 mM sodium phosphate buffer pH 6.8. Freshly isolated membranes were used immediately for enzymatic assays.

To measure NADH dehydrogenase I (NDH-I) activity, inverted membranes were isolated as described previously^26^. Briefly, the vesicles were obtained from cells grown aerobically in MOPS-GLC minimal medium to an OD_600_ of ~0.4. Bacterial cells were pelleted by centrifugation at 10,000 *g* for 15 min at 4 °C and washed twice with ice-cold 50 mM MES buffer pH 6.0 containing 10% glycerol. The resulting bacterial suspensions were disrupted by sonication in the same buffer. Cell debris was removed by centrifugation at 10,000 *g* for 15 min at 4 °C. Supernatants were spun at 100,000 *g* for 2 h at 4 °C in an Optima XL-100K ultracentrifuge (Beckman). The resulting inverted membrane vesicles were resuspended in ice-cold MES buffer. Protein concentrations were determined using the BCA Protein Assay kit (Thermo Scientific) according to manufacturer instructions.

### Enzymatic assays

DMSO and sulfate reductase activities were assayed under anaerobic conditions by monitoring the oxidation of benzyl viologen as previously described^6^. Reactions were carried out in a total volume of 1 ml in 20 mM sodium phosphate buffer pH 6.8 containing 0.5 mM dithiothreitol, 0.2 mM benzyl viologen, 100 μg of the reducing agent sodium dithionate, 100 μg freshly prepared membrane protein, and 2 mM DMSO or sulfate. Benzyl viologen was reduced with 150 μM sodium dithionite. Where indicated, 100 µg of affinity-purified Xsr3 or DmsABC complex were assayed for sulfate or DMSO reductase as measured by benzyl viologen oxidation. The oxidation of benzyl viologen was monitored at 575 nm using a CARY 60 UV–Vis spectrophotometer (Agilent) immediately after the addition of DMSO or sulfate. The enzyme activity was calculated using the extinction coefficient of benzyl viologen (ε_benzyl viologen_ = 8.9 mM^−1^ cm^−1^)^36^.

The activity of the NADH-dependent glutamate synthase (GOGAT) was measured kinetically by monitoring the oxidation of NADH at 340 nm^37^. *Salmonella* cultures grown aerobically overnight in LB broth at 37 °C in a shaker incubator were diluted to ~5 × 10^7^ cells per ml in MOPS-GLC minimal medium supplemented with 250 μM sulfate and cultured at 37 °C to OD_600_ of ~0.4. Where indicated, cells were challenged with 400 μM H_2_O_2_ for 30 min. Following treatment, cells were collected and resuspended in 25 mM HEPES buffer pH 7.5. The bacterial cells were disrupted by sonication. Cell-free extracts were obtained by centrifugation at 17,000 *g* for 15 min at 4 °C. Protein concentrations were determined using the BCA Protein Assay kit (Thermo Scientific). Enzyme reactions were performed in 25 mM HEPES buffer pH 7.5 containing 5 mM α-ketoglutarate, 5 mM L-glutamine, 3 mM NADH and 50 μg of protein. NADH oxidation was monitored over 3 min at 340 nm using a microplate reader (Molecular Device).

NDH-I activity was assessed by monitoring the oxidation of NADH at 340 nm^26^. Reactions were performed in 50 mM MES buffer pH 6.0 containing 10% glycerol and 200 μM of nicotinamide hypoxanthine dinucleotide (deamino-NADH, Millipore Sigma). NDH-I is known to utilize diamino-NADH and NADH with equal efficiency. To evaluate the direct effect of H_2_O_2_ on NDH-I activity in vitro, membrane vesicles isolated from *Salmonella* were exposed to 100 μM H_2_O_2_ for 10 min at 37 °C. Enzymatic activity was subsequently measured by tracking the oxidation of deamino-NADH in a microplate reader (Molecular Devices).

### Sulfate consumption

Membranes and cytosolic fractions were isolated from *Salmonella* grown overnight aerobically in LB broth as described^6^. After ultracentrifugation, sulfate reductase activity was measured in both the membrane and cytosol fractions as described in the ‘Enzymatic Assays’ section. To assess the dependence on quinone electron carriers, 1 mg ml^−1^ ubiquinone-10 (coenzyme Q10, Millipore Sigma) or menaquinone-4 (MK-4, Millipore Sigma) was added in place of benzyl viologen. Selected reactions contained 0.2 mM ATP. After 20 min, sulfate concentration in the reaction mixtures was quantified using a sulfate test kit (Millipore Sigma) according to manufacturer instructions.

### Measurement of intracellular sulfite

*Salmonella* cultures grown aerobically overnight in LB broth at 37 °C were diluted to ~5 × 10^7^ cells per ml in 3 ml MOPS-GLC minimum medium supplemented with either 250 μM sulfate or sulfite. Cultures were incubated overnight at 37 °C under hypoxic conditions. Cells were collected by centrifugation at 17,000 *g* for 1 min, and pellets were resuspended in 250 μl of deionized water. Cells lysed by sonication were centrifuged at 17,000 *g* for 15 min at 4 °C. The concentration of sulfite in the resulting supernatants was quantified using the sulfite test kit (Millipore Sigma) according to manufacturer instructions. Intracellular sulfite concentration was normalized to OD_600_.

### Hydrogen sulfide measurements

Intracellular H_2_S concentrations in *Salmonella enterica* serovar Typhimurium were assessed using the specific fluorescent probes WSP-5 (Cayman Chemical) or 7-azido-4-methylcoumarin (Millipore Sigma) as previously described^6^. Bacteria were cultured in MOPS-GLC minimum medium containing 250 μM of either sulfate or sulfite as the sole sulfur source under aerobic conditions to OD_600_ of ~0.4. Where indicated, some of the bacteria were grown standing for 6 h. After incubation for 30 min with 10 μM of the designated H_2_S probe, the bacteria were pelleted by centrifugation at 15,000 *g* for 3 min. After washing once with PBS to remove excess probe, the bacterial pellets were resuspended in 2 ml PBS and incubated at room temperature for 30 min. Fluorescence intensity was measured using a Citole S fluorimeter (Beckman Coulter) at excitation/emission wavelengths of 500/533 nm. Relative fluorescence units were normalized to OD_600_ after subtraction of background fluorescence. Where indicated, H_2_S production was assessed in vitro using WSP-5 fluorescent probes on membranes isolated from *Salmonella* strains as described above. H_2_S production was normalized per μg of membrane protein.

### RNA isolation and quantitative real-time PCR

*Salmonella* cultures grown aerobically overnight in LB broth at 37 °C were diluted to ~5 × 10^7^ cells per ml in MOPS-GLC minimal medium supplemented with 250 μM sulfate. Total RNA was extracted from bacterial cultures grown to OD_600_ of ~0.4 under aerobic or anaerobic conditions using the High Pure RNA Isolation kit (Roche) according to protocols provided by the manufacturer. Complementary DNA (cDNA) was synthesized from 1 μg of purified RNA using Luna Universal Probe qPCR Master Mix (NEB) and 10 μM of N6 random primers (Thermo Fisher). Quantitative real-time PCR was carried out using 10 μM gene-specific primers and 10 μM probes containing 5′6-carboxyfluorescein and 3′ black-hole quencher 1 modifications (Supplementary Tables). Standard curves were generated using PCR-amplified DNA fragments containing target gene sequences. Transcript abundance was normalized to the internal *rpoD* housekeeping gene.

### EPR spectroscopy

Electron paramagnetic resonance was conducted in 20 mM sodium phosphate buffer pH 6.8 containing 2 mM sodium dithionite and 10 mg ml^−1^ membrane protein preparations isolated as described above. Reactions were conducted in the presence or absence of 50 mM DMSO or sulfate in a Bactron anaerobic chamber (Shel Lab) for 20 min. Samples were flash frozen in liquid nitrogen after loading into PTFE tubing. Measurements were performed using a finger dewar and liquid nitrogen. Spectra were recorded using an EMXnano Bruker spectrometer under the following conditions: 77 K temperature, 10 mW microwave power, 9.65 GHz microwave frequency and 6 G modulation amplitude. Traces are the average of 10 scans.

### ΔpH measurements

Cytoplasmic pH was assessed using the ratiometric, pH-sensitive GFP pHluorin derivative, expressed from the pBAD promoter as previously described^6,38^. Briefly, *Salmonella* harbouring the pHIuorin plasmid was grown aerobically overnight in LB broth supplemented with 0.2% arabinose and 100 μg ml^−1^ penicillin. Cultures were diluted to ∼ 2 × 10^8^ cells per ml in MOPS-GLC minimum medium containing 250 μM of either sulfate or sulfite, or 100 μM sulfide and equilibrated for 10 min at 37 °C. Fluorescence spectra (excitation at 300–490 nm, emission at 510 nm) were recorded for 4 min using a Shimadzu R5300C spectrofluorometer (Shimadzu). Bacterial cells were challenged with 400 μM H_2_O_2_, and spectra were monitored for 25 min. To determine the cytoplasmic pH values, fluorescence ratios (405/488 nm) were calibrated using cells equilibrated in MOPS-GLC media at pH 5.0–8.5 in the presence of protonophore potassium benzoate. Internal pH was calculated using a Boltzmann sigmoid best-fit curve.

### ATP quantification

Intracellular ATP levels were measured using a luciferase-based ATP Determination kit (Molecular Probes, Thermo Fisher). *Salmonella* grown aerobically overnight in LB broth at 37 °C were diluted to ~5 × 10^7^ cells per ml in MOPS-GLC minimum medium supplemented with 250 μM of either sulfate or sulfite. Bacterial cultures were incubated in a 1% O_2_ hypoxic chamber for 4 h at 37 °C. Where specified, some specimens were treated with 400 μM H_2_O_2_ for 30 min. Bacteria were collected by centrifugation at 15,000 *g* for 3 min. ATP in bacteria was extracted in 0.5 ml of ice-cold 380 mM formic acid containing 17 mM EDTA. The resulting specimens were pelleted for 1 min at 16,000 *g*. Supernatants were diluted 25-fold into 100 mM *N*-tris(hydroxymethyl)methyl-2-aminoethanesulfonic acid^13^ buffer pH 7.4. Samples or ATP standards (10 μl) were combined with 90 μl of a master mix consisting of 8.9 ml of water, 500 μl 20× buffer (200 mM Tris pH 7.5, 2 M NaCl, 20 mM EDTA and 0.2% Triton X-100**)**, 500 μl 10 mM D-luciferin, 100 μl 100 mM dithiothreitol and 2.5 μl 5 mg ml^−1^ firefly luciferase. The specimens were incubated for 5 min at room temperature in the dark. Luminescence was measured using an Infinite 200 PRO instrument (Tecan). ATP concentrations, determined by linear regression using ATP standards, were normalized to c.f.u.s ml^−1^.

### NADH and NAD^+^ measurements

Intracellular concentrations of NADH/NAD^+^ were determined as previously described^6^. *Salmonella* cultured aerobically overnight in LB broth at 37 °C were diluted to ~5 × 10^7^ cells per ml in MOPS-GLC minimal medium. Cells were cultured aerobically in MOPS-GLC minimal medium at 37 °C to OD_600_ of ~0.4. NADH and NAD^+^ were separately extracted from cell pellets obtained from 1.6 ml cultures using 120 μl 0.2 M NaOH and 0.2 M HCl, respectively. Extracts were neutralized by the addition of equal volumes of 0.1 M HCl (for NADH) or 0.1 M NaOH (for NAD^+^). Of the neutralized extracts, 10 μl were added to a 90 μl reaction containing 100 mM bicine pH 8.0, 4 mM EDTA, 1.6 mM phenazine methosulfate, 0.42 mM 3-(4,5-dimethylthiazol-2-yl)-2,5-diphenyltetrazolium bromide and 10% ethanol. Reactions were initiated by adding 100 μl 0.4 μg alcohol dehydrogenase (Millipore Sigma). Absorbance was measured spectrophotometrically at 570 nm using a microplate reader (Molecular Devices). NADH and NAD^+^ concentrations were calculated on the basis of standard curves and normalized to OD_600_.

### MopB superfamily sequence retrieval and curation

To place the *Salmonella* DmsA catalytic subunit within the context of the MopB superfamily, we first used the DmsA amino-acid sequence from *S. enterica* serovar Typhimurium 14028s as a profile HMM seed in jackHMMER (HMMER v.3.4) searches against the UniRef90 protein database. Searches were run for five iterations using a stringent reporting threshold (-E 1 × 10^−10^) while retaining default inclusion thresholds. These analyses recovered 246,760 candidate MopB-domain proteins distributed across major bacterial and archaeal phyla. To enrich bona fide MopB catalytic subunits, the hits were filtered by the presence of a conserved C-terminal MopB catalytic core and an N-terminal [4Fe–4S] cluster using NCBI’s Conserved Domain Database (CDD) and Pfam domain annotations. Truncated proteins and sequences falling outside the expected size range for MopB-family members were excluded from further analyses. Redundant sequences were collapsed using MMseqs2 with a sequence identity threshold of 90% and default coverage parameters. Obsolete or deprecated UniProt entries were excluded, yielding a curated set of 3,307 non-redundant MopB/[4Fe–4S]-containing proteins. MopB homologues (17) encoded by the *S. enterica* 14028s genome, identified by BLASTp/DELTA-BLAST within *Salmonella* (TaxID 588858), were then appended to this dataset to ensure comprehensive representation of *Salmonella* paralogues in the global MopB tree, resulting in a total of 3,324 sequences for global MopB phylogenetic analysis.

### Multiple sequence alignment and global MopB phylogeny

Amino acid sequences in the 3,324-sequence dataset were aligned using MAFFT v.7.515 with the L-INS-i algorithm and the options ‘–localpair–maxiterate 1000’ to optimize alignment accuracy in the presence of variable insertions and deletions. Obvious low-complexity regions, long indels and poorly aligned positions were pruned using trimAl v1.4 with the ‘-gappyout’ setting, and the resulting alignment was visually inspected in Jalview v.2.11.2 to confirm the integrity of the conserved MopB catalytic motifs and N-terminal [4Fe–4S] cluster regions. Maximum-likelihood phylogenetic reconstruction was performed with IQ-TREE2 (v.2.x) using ModelFinder Plus (MFP) to select the best-fitting amino-acid substitution model under the Bayesian information criterion. The MFP procedure favoured an LG + R mixture model with discrete site-rate heterogeneity. Tree searches were conducted with default nearest-neighbour interchange and subtree-pruning-regrafting moves, and node support was assessed using 1,000 ultrafast bootstrap (UFBoot) replicates. The resulting tree was annotated with taxonomic and NCBI CDD accessions, and clades were assigned to MopB_CT_Fdh-Nap-like (CD00508), MopB_CT_Nitrate-R-NapA-like (CD02791), MopB_Formate-Dh-H (CD02753), MopB_CT_Thiosulfate-R-like (CD02778/2781), MopB_CT (CD02775), MopB_3 (CD02766), MopB_Arsenate-R (CD02757) and MopB_DmsA_EC (CD02770) on the basis of the majority CDD call for each terminal and its surrounding clade. To root the global MopB tree, we used the MopB_Formate-Dh-H (CD02753) family as an outgroup, guided by previous DMSO reductase superfamily research^39^.

### Definition and subsampling of the DmsA_EC clade

Within the rooted global MopB tree, we operationally defined the MopB_DmsA_EC clade as the set of sequences carrying the DmsA_EC CDD signature (CD02770) that formed a single, well-supported monophyletic group reciprocally exclusive of other MopB families. This radiation encompassed 254 sequences (~8% of the global MopB dataset), which were taxonomically annotated at the phylum level using NCBI Taxonomy and the Genome Taxonomy Database (GTDB), where available. To generate a reduced taxonomically representative dataset suitable for detailed phylogenetic and temporal analyses, the 254 DmsA_EC sequences were clustered using CD-HIT (v.4.8.1) with a 90% amino-acid identity threshold, retaining the longest representative from each cluster to preserve alignment quality and phylogenetic signal. This procedure yielded 60 non-redundant exemplars. This set was manually augmented with the 5 chromosomal DmsA-like paralogues from *S. enterica* 14028s (STM1089, STM1809, STM1811, STM3103, STM5178), 11 well-annotated enterobacterial homologues selected on the basis of *dmsABC* operon context and 4 MopB_Arsenate_R outgroup sequences, resulting in an 80-sequence dataset.

### Focused DmsA_EC alignment and maximum-likelihood tree

The 80-sequence DmsA_EC dataset was aligned de novo using MAFFT L-INS-i with ‘–localpair–maxiterate 1000’ and inspected to ensure the conservation of catalytic motifs, [4Fe–4S] cluster ligands and MOCO-binding residues. Ambiguously aligned terminal regions and rare long insertions were trimmed with trimAl ‘-gappyout’. Visual curation in Jalview was used to remove residual poorly aligned segments and to ensure positional homology in the catalytic core. The final curated alignment comprised 80 sequences and 646 amino-acid positions.

A maximum-likelihood phylogeny was then inferred in IQ-TREE2 under the LG + F + R6 substitution model (empirical amino-acid frequencies and 6-category FreeRate heterogeneity), which was selected by ModelFinder as the best-fitting model for this reduced dataset. Bootstrap support was estimated with 1,000 UFBoot replicates. To infer branching direction, the focused DmsA_EC tree was rooted using 4 representative sequences from the MopB_Arsenate-R family (CD02757); 2 from Bacillota and 1 each from Pseudomonadota and Thermodesulfobacteriota, selected from the immediate sister clade to DmsA_EC identified in the global MopB phylogeny (Extended Data Fig. 8). The sister relationship between the Arsenate-R and DmsA_EC families is robustly supported across all tree topologies in previous comprehensive MopB superfamily phylogenies^7^, establishing the arsenate-R family as the appropriate outgroup for a focused DmsA_EC analysis. The use of the most closely related sister family as outgroup, rather than more distantly related sequences, follows current best practice for phylogenetic rooting to minimize random rooting artefacts^40^. Major bacterial clades were identified from taxonomic metadata and annotated using iTOL.

### Taxonomic distribution analysis

Organism names recovered at each filtering step were mapped to NCBI taxonomy using the R package taxonomirz, querying a local SQLite database built from NCBI taxonomy files downloaded in April 2026. Phylum-level representation was calculated as the percentage of sequences assigned to each phylum at each filtering step and visualized as a heat map using the pheatmap package, with colour intensity reflecting sequence representation on a log_10_ scale. Phyla contributing less than 0.1% of sequences across all filtering steps were excluded for clarity. Apparent absences of specific lineages in the final dataset may reflect gaps in database coverage rather than true biological absence, as sequences were retrieved from UniRef90 rather than a defined genome survey.

### Relative-rate timetrees under alternative substitution models

To evaluate the robustness of branching order and relative node ages to substitution model choice, we generated uncalibrated (relative-rate) molecular timetrees for the 80-sequence dataset under the LG + F + Γ+I (LG + F + G + I) model. Because RelTime does not support FreeRate models, Γ-based approximations were used for relative-rate and calibrated analyses. For each model, branch lengths were re-estimated on the fixed maximum-likelihood topology using IQ-TREE2 with the specified model and default optimization, and relative divergence times were inferred with the RelTime-ML method implemented in MEGA X v.12, scaling the root age arbitrarily to 1.0. Because we assessed for stability in the relative ordering and depth of key internal nodes, no external calibrations were imposed in these analyses. The relative timetrees, which were constructed using LG + F, LG + F+ Γ and LG + F + Γ+I amino-acid substitution models, were compared visually by inspecting relative node heights. The visual analysis revealed that the timetrees generated by the LG + F, LG + F + Γ and LG + F + Γ+I were highly concordant, justifying the use of LG + Γ+F for the subsequent calibrated RelTime analysis.

### Calibrated molecular-clock analysis and geochemical overlays

Absolute divergence times for DmsA_EC lineages were estimated using the RelTime-ML approach in MEGA X v.12 with the LG + Γ+F model and the 80-sequence alignment and topology described above. Because reliable fossil calibrations are not available for this single-gene family, we applied a small set of soft calibration bounds as secondary temporal constraints to anchor deep and intermediate nodes in a manner consistent with published bacterial phylogenomic timetrees and broad Earth redox history. Specifically, we constrained: (1) the split between the bacterial Arsenate-R outgroup and the Bacillota DmsA_EC lineage with a minimum bound of 2,500 Mya and a maximum bound of 3,500 Mya, consistent with the estimated timing of major bacterial phylum divergences and the Archaean origin of arsenate respiration^7^; (2) Actinomycetota–Campylobacteriotota split within the DmsA_EC clade to 1,200–2,000 Mya; and (3) the Gammaproteobacterial DmsA_EC radiation to 500–800 Mya. All constraints were implemented as soft bounds to permit moderate departures from these ranges during rate smoothing. Confidence intervals for node ages were obtained using MEGA’s default procedures. These calibrations were used to provide relative temporal ordering and approximate geological placement of major radiations. Given the limitations of single-gene molecular dating, absolute ages were interpreted cautiously and treated as approximate.

### [Fe–S] motif analysis and mapping onto phylogeny

To examine the diversity of [4Fe–4S] [Fe–S] coordination motifs within the DmsA lineage, we extracted the [Fe–S]-bearing region from the trimmed 80-sequence alignment and scanned for conserved cysteine patterns in Python. Motifs were classified on the basis of the spacing of cysteine residues in positions corresponding to the [Fe–S] motif, as inferred from alignment to the *E. coli* DmsA structure (PDB ID 1EU1). [Fe–S] motif classes were then mapped onto the branches of the DmsA tree through iToL annotations.

### Structural modelling

The five DmsA paralogues in *S. Typhimurium* 14028s were selected for structural analysis. Predicted three-dimensional structures of all five proteins were generated using AlphaFold3 via the AlphaFold Protein Structure Database or locally implemented in ColabFold with default settings. The top-ranked predicted structure for each protein, based on predicted local distance difference test (pLDDT) scores, was used for downstream analysis. Electrostatic surface potentials were computed in UCSF ChimeraX (v.1.6)^41^ using the Coulombic Surface Coloring tool. Default dielectric parameters were applied. The surfaces are coloured from red (negative potential) to blue (positive potential), highlighting charge distributions across the solvent-accessible surfaces. All five protein structures were oriented consistently to facilitate visual comparison. Pairwise sequence similarity between DmsA and each of the other four proteins was calculated using the EMBOSS Needle tool with default gap penalties and the BLOSUM62 matrix. Percentage identity values are annotated in blue text on the figure next to each corresponding structure. For the predicted molybdenum-bis pyranopterin guanosine dinucleotide (Mo-bis PGD) binding sites and structural model of Xsr3A, cofactor binding sites were modelled by structurally aligning the predicted protein models multiplicity of infection to the crystal structure of *E. coli* DmsA (PDB ID 1EU1) using the MatchMaker tool in ChimeraX. Coordinates of the Mo-bis PGD cofactor and proximal Fe–S clusters were extracted from the reference structure and fitted into the homologous binding clefts in DmsA and Xsr3A. Key coordinating residues were identified on the basis of geometric proximity (<4 Å) and canonical motifs conserved in the DMSO reductase family. Interacting residues were visualized in stick representation, and hydrogen bonds or salt-bridge interactions were annotated.

### Statistics

Statistical analysis was performed using GraphPad Prism v.10.4. Quantile–quantile plots were used to determine normality. One-way and two-way analysis of variance (ANOVA), and *t*-test were performed with *P* < 0.05 as the cut-off for statistical significance. Error bars indicate mean ± s.d. Mouse group sizes were determined using power analysis informed by variability observed in previous experiments of similar design. Group sizes of 4–10 animals were sufficient to provide 80% statistical power at *α* = 0.05 for detecting expected differences in bacterial burden. Male and female mice were randomized across groups. Mice were housed in groups of 3–5 in ventilated cages. No statistical methods were used to pre-determine sample sizes of all other experiments that did not involve mice, in which case sample sizes were chosen on the basis of those reported in previous publications^5,6^. Statistical tests, *P* values and replicate counts are listed in figure legends. Except for mouse experiments, all other data collection and analysis were not performed blind to the conditions of the experiments in this study. No animals or data points were excluded from the analyses.

### Reporting summary

Further information on research design is available in the Nature Portfolio Reporting Summary linked to this article.

## Supplementary information


Supplementary InformationLegend for Supplementary Data 1, References for supplementary tables, Supplementary Figs. 1 and 2, And uncropped image of Coomassie-stained gel for Extended Data 5c.
Reporting Summary
Peer Review file
Supplementary Data 1Excel workbook containing multiple sheets corresponding to the sequence sets obtained at each step of the analysis pipeline, illustrated in Extended Data Fig. 8a, can be seen in the Supplementary Data File. Starting from iterative JackHMMER searches, sequences were progressively filtered through domain-based screening, redundancy reduction using MMseqs2, removal of obsolete UniProt entries, and verification of conserved domains using the NCBI Conserved Domain Database (CDD). The final dataset was further curated to remove highly similar sequences, resulting in the representative set used for phylogenetic reconstruction. Each sheet lists the protein accessions and associated metadata corresponding to the output of that step.
Supplementary Data 2Bacterial strains, plasmids and oligomers.


## Source data


Source Data Figs. 1–5 and Extended Figs. 1–10Source data.


## Data Availability

Data and materials generated during these investigations are included in the main document, Extended Data figures, Extended Data tables, Supplementary Data file and accompanying Source Data files. Source data are provided with this paper.
