# Supplementary material for: Salmonella uses sulfate reductases with unique catalytic activity to promote gut colonization in mice
Source: Nat Microbiol. 2026 Aug 5;11(9):2619–33. doi: 10.1038/s41564-026-02443-y (PMC13524816; doi:10.1038/s41564-026-02443-y)

nature microbiology

Supplementary information

https://doi.org/10.1038/s41564-026-02443-y

Salmonella uses sulfate reductases with unique catalytic activity to promote gut colonization in mice

In the format provided by the authors and unedited

Salmonella uses sulfate reductases with unique catalytic activity to promote gut colonization in mice

Ju-Sim Kim[1], Siva Uppalapati[1], Alyssa Margolis[1], Michael McClelland[2], Hanan Elajaili[3], Eva Nozik[3], Lin Liu[1], and Andres Vazquez-Torres[1,4*]

[1]University of Colorado School of Medicine, Department of Immunology and Microbiology, Aurora, Colorado-80045, USA
[2]University of California, Irvine, School of Medicine, Department of Microbiology and Molecular Genetics, Irvine, CA, USA
[3]Cardiovascular Pulmonary Research Laboratories and Pediatric Critical Care Medicine, University of Colorado Anschutz Medical Campus, Aurora, Colorado-80045, USA
[4]Veterans Affairs, Eastern Colorado Health Care System, Aurora, Colorado-80045, USA

Lead contact: Andres Vazquez-Torres
Department of Immunology and Microbiology
University of Colorado School of Medicine
12800 East 19th Avenue MS 8333
RC1 North room P18-9131
Aurora, Colorado 80045, USA
Email: andres.vazquez-torres@cuanschutz.edu

Supplementary Data 1: Sequence datasets generated at each stage of the DmsA/MopB phylogenetic pipeline.

References for Supplementary Tables

1. Kim, J.-S. *et al.* Anaerobic respiration of host-derived methionine sulfoxide protects intracellular *Salmonella* from the phagocyte NADPH oxidase. *Cell Host Microbe* **32**, 411-424.e10 (2024).
2. Chakraborty, S. *et al.* Glycolytic reprograming in *Salmonella* counters NOX2-mediated dissipation of ∆pH. *Nat. Commun.* **11**, 1783 (2020).
3. Hanahan, D. Studies on transformation of *Escherichia coli* with plasmids. *J. Mol. Biol.* **166**, 557–580 (1983).
4. Rong Fu Wang & Kushner, S. R. Construction of versatile low-copy-number vectors for cloning, sequencing and gene expression in *Escherichia coli*. *Gene* **100**, 195–199 (1991).
5. Datsenko, K. A. & Wanner, B. L. One-step inactivation of chromosomal genes in *Escherichia coli* K-12 using PCR products. *Proc. Natl. Acad. Sci.* **97**, 6640–6645 (2000).
6. Miesenböck, G., De Angelis, D. A. & Rothman, J. E. Visualizing secretion and synaptic transmission with pH-sensitive green fluorescent proteins. *Nature* **394**, 192–195 (1998).

Supplementary Figure1: Identity and similarity of DmsA (STM14_1089) paralogs.

	Identity	Similarity
STM14_1809	66%	80%
STM14_1811	68%	80%
STM14_3103	40%	58%
STM14_5178	49%	64%

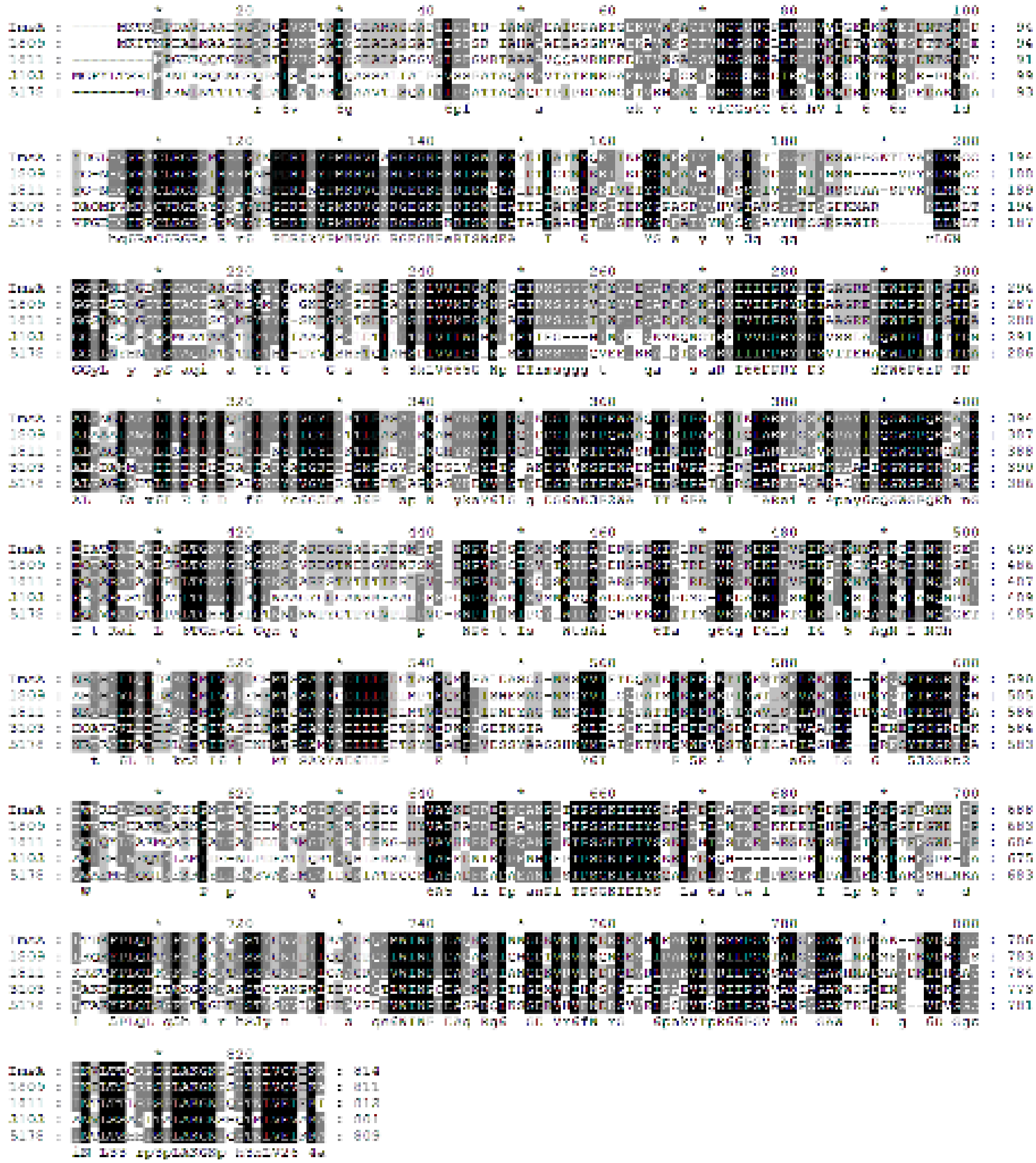

Supplementary Figure 2. Molecular mechanisms by which extracytoplasmic sulfate reductases promote *Salmonella* virulence. (Center panel), nontyphoidal *Salmonella* express three extracytoplasmic sulfate reductases (XsrABC) in the periplasmic space that reduce sulfate (SO_4^{2-}) to sulfite (SO_3^{2-}). SO_3^{2-} is reduced in the cytoplasm to hydrogen sulfide (H_2S), a gas that is incorporated into biomass via cysteine. For example, cysteine desulfurases such as the Isc system help assimilate sulfur from sulfate into [Fe-S] clusters. XsrABC enzymatic complexes operate under aerobic, and especially anaerobic, conditions, drawing reducing power from either ubiquinone (UBQ) or menaquinone (MNQ). Assimilation of sulfate by the enzymatic activity of XsrABC complexes helps *Salmonella* overcome colonization resistance in the gut (top panel), while resisting the antimicrobial activity of reactive oxygen species produced by the phagocyte NADPH oxidase of macrophages. H_2S produced downstream of XsrABC enzymatic activity may directly inhibit gut microbiota and scavenge ROS. In addition to playing direct effector roles, incorporation of H_2S into biomass my foster direct growth of *Salmonella* in gut and viscera. XsrABC complexes may enable *Salmonella* to compete with microbiota for SO_4^{2-} as a unique sulfur source in the gut. Artistic rendering by Enrique Bonet.

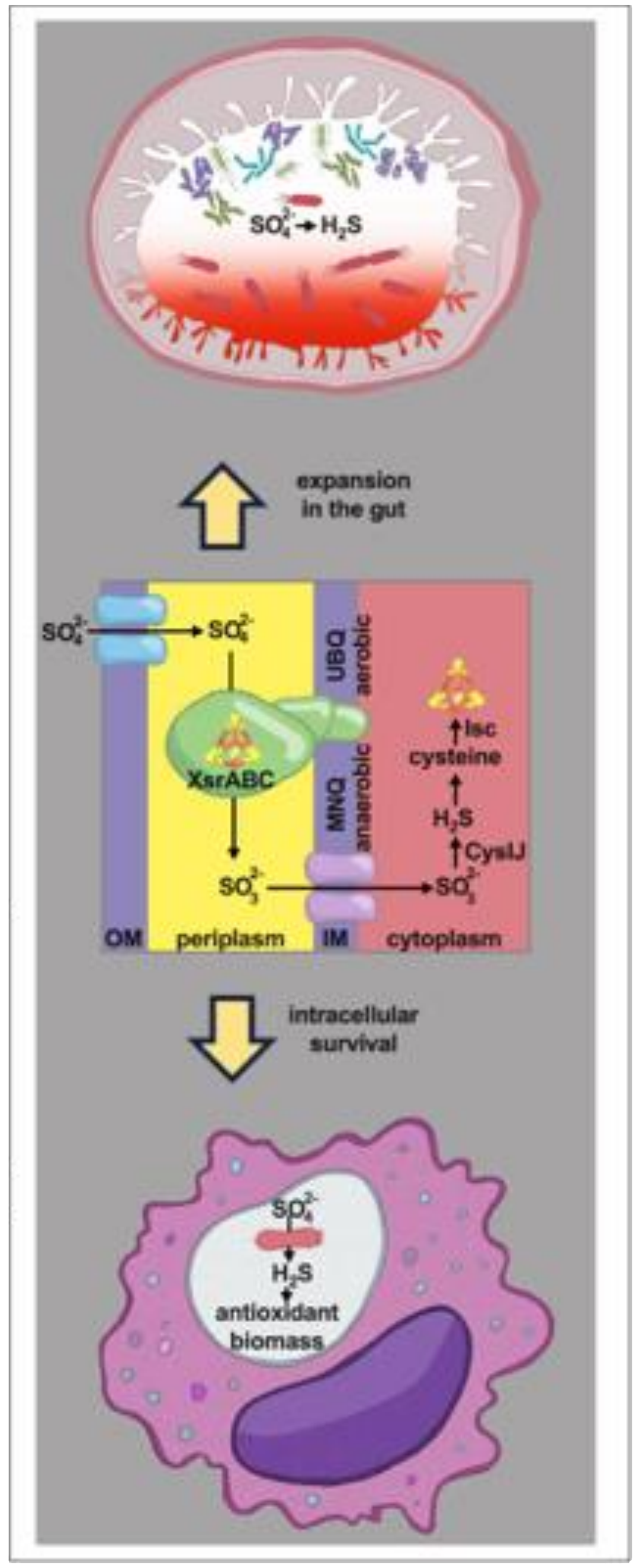

Uncropped image of Coomassie-stained gel for Extended Data 5c

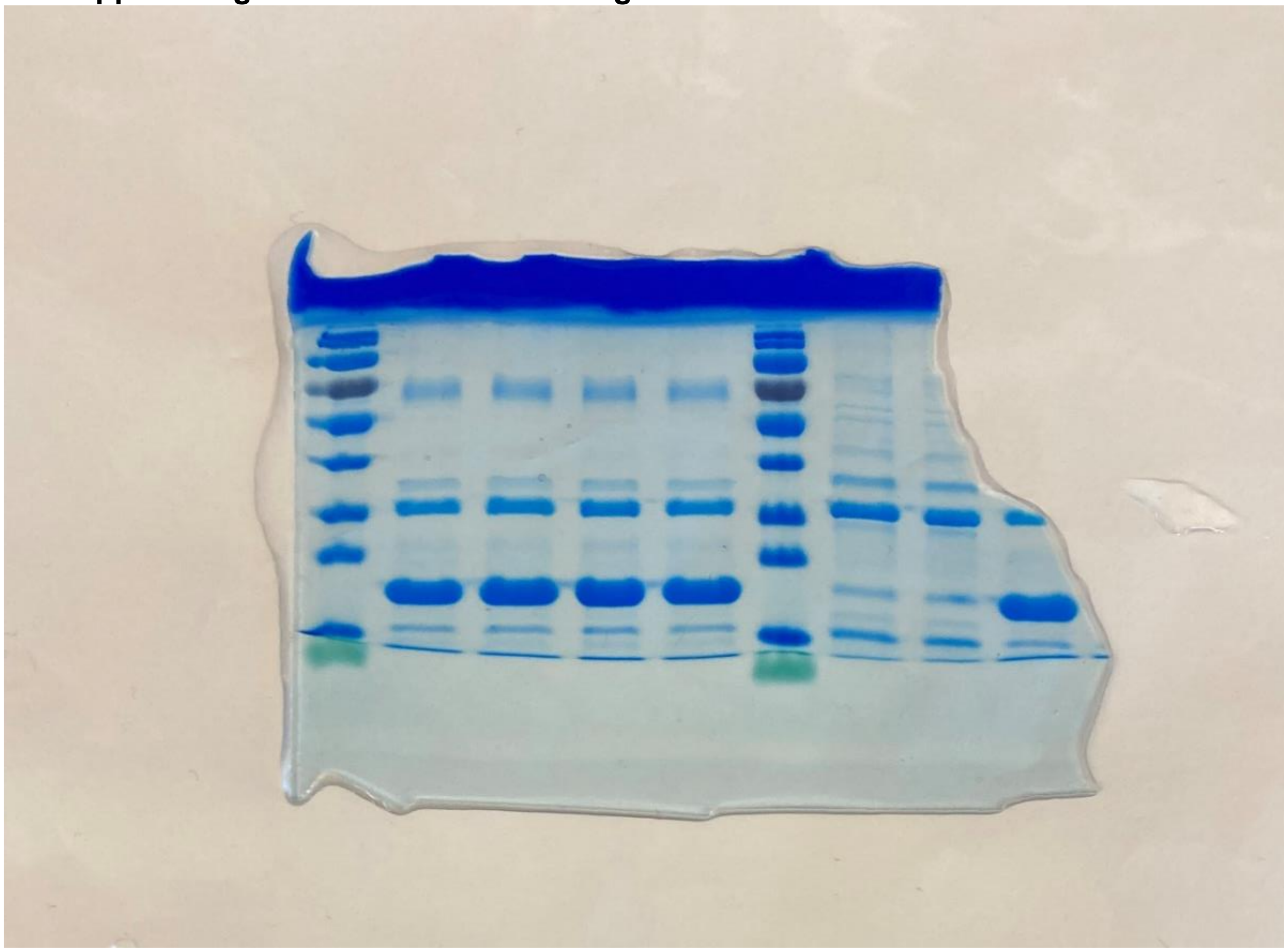

Supplement: Supplementary file 1 [file 41564_2026_2443_MOESM1_ESM.pdf]
