# Supplementary material for: Salmonella uses sulfate reductases with unique catalytic activity to promote gut colonization in mice
Source: Nat Microbiol. 2026 Aug 5;11(9):2619–33. doi: 10.1038/s41564-026-02443-y (PMC13524816; doi:10.1038/s41564-026-02443-y)
Supplement: Supplementary file 3 [file 41564_2026_2443_MOESM3_ESM.pdf]

nature portfolio

Peer Review File

Salmonella uses sulfate reductases with unique catalytic activity to promote gut colonization in mice

Corresponding Author: Professor Andres Vazquez-Torres

Version 0:

Reviewer comments:

Reviewer #1

(Remarks to the Author)
Kim et al. report on a novel DMSO reductase paralog involved in sulfur assimilation in a strain non-typhoid Salmonella. The work involved animal and macrophage studies, genetic knock-outs, growth, peroxide, and enzyme assays, transcription, EPR spectroscopy, bioinformatics, structural modeling, and phylogenic analyses. It is a continuation of their study published in Cell Host Microbe (Kim et al., 2024) in which they demonstrate macrophage generated methionine sulfoxide as an an alternative electron acceptor in anaerobic respiration in infectious Salmonella. While they demonstrated the involvement of a DMSO reductase (DsmABC)in the previous paper, here the focus is on three additional paralogs found in the genome. They propose that one of the paralogs does so by a mechanism that is MOCO independent.

While the discovery of a MOCO independent mechanism for methionine sulfoxide reductase would be novel, the manuscript suffers from trying to cram too many ideas into a short manuscript. The figures have way too many panels, many which don't relate, and sparse information in the legends. Data is shown but not fully explained and related figures are scattered between the main text and supplemental materials. The figures need to be reorganized so that related data isn't scattered. Figure 1a, 3a,b,c,d, and 4e are not necessary.

Reading the previous publication was also helpful in figuring out the rationale for the work. The evolution aspect detracts from the paper and has little relevance to what the title eludes to. What does the GOE have to do with Salmonella virulence?

Why are the paralogs "orphans"? What evidence is there that they are periplasmic? Please identify the leader sequence that targets the periplasm (this can be added to the alignment figure). The naming convention is also incorrect. Psr is already taken (polysulfide reductase). If these paralogs are indeed periplasmic then it should be Srp.

The models in figure 2b seem out of place with the rest of the data in the figure and are way too small. One can gather from the figures and Table S2 that a great number of mutants were generated to demonstrate that MOCO is not required in the paralogs, but few are identified in the text. The results presented in the figures need to be better explained in the text. How was it determined that all the subunits were necessary? How was it determined that the EPR spectra was due to the FS0 when only membrane fractions were used and not pure protein? And what evidence suggests that "these periplasmic sulfate reductases still load MOCO...for an undiscovered activity". Modeling is suggestive and EPR of membranes is not enough. Additional structural needs to be done. Lastly, there is a recent review (2024) by Hasnat and Leimkuehler (https://doi.org/10.1016/j.bbamcr.2024.119731) "Shared functions of Fe-S cluster assembly and Moco biosynthesis" that seems relevant to the current study.

Reviewer #2

(Remarks to the Author)
This manuscript entitled “Molybdenum-independent [4Fe-4S]-catalyzed Sulfate assimilation sustains Salmonella virulence” reports the identification and characterization of novel sulfate reductases. First the authors identify 4 paralogs of DmsA in Salmonella and show that they are responsible for protection against host during infection. Then, they show that they exhibit a sulfate reductase activity and suggest that the activity relies on [Fe-S] cluster rather than MoCo. Finally, the authors show the effect of these sulfate reductases on sulfur assimilation. The analysis is well designed and the experimental results are convincing. Nevertheless, it is on several occasions hard to read, with logical gaps and assumptions that appear at a first glance unjustified. I encourage the authors to follow the rigorous scientific framework throughout the manuscript, with a clear progression of i) hypothesis ii) method iii) observation iv) interpretation and not aggregate more and more results into single conclusions. Furthermore, the phylogenetic study needs further clarifications and expanded analyses. Overall, I find the analysis itself interesting but the manuscript in its current form struggles to capture the interest, especially for a broad audience.

Major comments

1) The phylogenetic analysis suffers from some issues and needs clarification.

A) To reach the conclusions that the authors claim, they have to carry a careful analysis in a database covering the diversity of prokaryotes, and from the M&M, one can only trust the authors with "a comprehensive prokaryotic protein database". The authors should provide the exhaustive list of genomes used, with quality parameters such as completeness and contamination percentage, and explain explicitly how this selection of genomes covers the diversity of prokaryotes.

B) I believe the authors are not specialists in phylogenetic analysis, as they appear to mix different concepts and overlook several important aspects required for robust inference. For example, the authors mix the phylogenetic inference with the dating of the phylogeny. I understand that the software infers both in a row but there are two distinct processes behind. That said, the authors should provide the phylogeny that is not dated, as for now, it is impossible to determine if the multifurcation in the dated tree is due to week support, short branches, or datation.

C) The robustness of branches should be assessed by bootstrapping.

D) The model of inference is not explicitly mentioned, a LG+G4+F is mentioned but only for the estimation of branch length during dating, which I think corresponds to the overall evolutionary model for phylogenetic inference.

E) The model used for datation is also not explicitly mentioned (strict molecular clock? Relaxed clock?). "divergence times were calibrated": what does "clean" mean? What does "taxon sister splits" means? Internal node? What does "support by operon context" means?

F) The characteristic of the alignment used for phylogeny should be provided (nb sequences and nb positions). Files should be provided in supplementary data (alignment, trimmed alignment, tree, outputs).

G) The sentence "The cross-species phylogenetic…" is vague and badly phrased (i.e. we do not "build" a phylogenetic analysis from queries). From what I understood, the authors used NCBI BLAST starting from 5 initial sequences, the hit resulting sequences have been used to build HMM profile using JackHMMER, used to query the local database. Rephrase and be more precise.

H) The taxonomic distribution should be presented, with presence/absence in each genome that the authors tested (Mapped on a species tree or at least in a table).

I) The authors should compare their results with that of Wells 2023.

2) The results and interpretation of phylogenetic analysis is not accurate.

A) "Molecular clock analysis placed" This is the phylogenetic tree that indicates the emergence of DmsA. I do not see any archaea in the phylogeny! "the time tree topology" Again, this is not the time tree, this is the tree topology. Overall, this sentence is not well written and the text is decorrelated to the figure. Where are the low-oxygen/sediment niches indicated? Where is Psr2? Be consistent with naming. Why underappreciated? It needs to be compared to literature.

B) "distant Proteus" This is relative, but at the scale of Bacteria, Proteus and Salmonella are very close from each other in the phylogeny (both Enterobacterales).
"indicating that this operon…" Overstatement. Every gene is retained in some species! This is the principle of natural selection and evolution. If the authors want to show that these genes are conserved, they should show the taxonomic distribution mapped on a species tree or at least in a table.

C) "The lack of synteny…":
- The figure S5 is hard to understand and the comparison between 3a and S5 is very challenging. The authors have to map the genomic context on the phylogeny (i.e. use iTOL).
- "The lack of synteny in deeper branches" Did the authors infer the genomic organization in deep branches? Otherwise rephrase.
- I do not see how a "lack of synteny" could "indicate" HGTs. The major tool to point HGTs is the phylogenetic tree and the mismatch between gene tree and species tree.
- Why to precise "over 100 million years"?
- "The group containing DmsA/Psr2A/Psr3A shares a characteristic $C3XC3XCX_nC$ FS0 architecture, whereas the ancestral Psr1A clade originating earlier in evolution bears a $C4XC3XCX_nC$" No indication on the phylogeny, hard to follow. The clade with C4XC does not branch earlier than the other clades. "ancestral Psr1A": To claim this, authors should reconstruct ancestral states by ML methods.
- "DmsA phylogeny" is it DmsA-like or DmsA phylogeny? I am confused.

D) The rest of the paragraph
This section is highly speculative, should be smoothed, and maybe placed in the discussion. The dating results should be interpreted with extreme caution for several reasons: i) The maximum-likelihood approach is not the most appropriate method for this type of analysis; a Bayesian framework is generally more suitable (and the authors do not specify which model was used). ii) The dataset includes only a single gene, which provides very limited information for reliable dating. This is consistent with the multifurcations observed. iii) The reliability of the calibration points is not discussed, further undermining the credibility of the

temporal estimates.

3) A final figure with the proposed model is essential at the end of the manuscript.

Specific comments

The lack of line and page numbering is very inconvenient and makes the review process unnecessarily challenging.

“thereby enabling Salmonella to grow in gut” Hard to understand the causal link.

“biogeochemistry of Earth for over 2.5 billion years” Why so precise on the date?

“evolving prokaryotes and archaea with a panoply” bacteria instead of prokaryotes? Or eukaryota instead of archaea?

“The dmsA gene (STM14_1089) encodes a novel methionine sulfoxide reductase within the DMSO reductase superfamily, while retaining its ability to metabolize DMSO” This sentence is confusing. Mention the activity DMSO reductase first (coherent with the name), and then the novel activity. Otherwise rename the gene.

I have a doubt on the usefulness of Figure 1a. 1) the method is outdated (NJ method is no longer used) 2) the authors underuse the figure 3) A phylogenetic tree with a single genome is not particularly informative. An equivalent of Figure 3a should replace 1a. The authors could use the dataset from Wells 2023 and include salmonella sequences inside.

“indicating a series of duplication events“ It could also be horizontal gene transfers.

“Construction of defined mutants” What “defined” means? Do you mean mutants of individual genes?

The gene STM14_1811 is suddenly not mentioned anymore in the text and figures, why?

STM14_1811 is mentioned in “systemic tissue than…”” but it does not appear in Fig 1d and S2a.

Why keeping the delta4 although the role of 1811 is not particularly studied compared to other genes?

In paragraph starting from “when complemented with the low…” the correspondence between plasmids for complementation and genes across text and figures is very hard to follow, use a single name for plasmids and define it in the text directly.

“mutant strains also become virulent…” Cybb is never defined. Fig 1f S3a should be at the end of the sentence. I am not specialist on this type of analysis but the statement “become virulent” seems odd for me. Indeed, the mutants are as virulent as the WT (index = 1), so they just did not lose their virulence. Why C57BL/6 is not mentioned in the text for 1f?

“Consistent with this, Salmonella used the added functions of the four…” This conclusion seems like overinterpretation. Either expand the logic behind the argument or moderate the conclusions.

“Based on the substrate specificity” What “based on” means? Is it at the level of the test that the authors used or in terms of concept? Rephrase.

Remove “duplicated” and “independently”.

“40-70%” There is no clear relationship between percentage of identity and functon(some protein families can harbor a high evolution rate and still keep the same function, and on the opposite, some can be overall very conserved in sequence but with different functions).

“encode hitherto unknown periplasmic” I did not follow why they are periplasmic, why not associated to the membrane in the cytoplasm or in the membrane? Did the authors predict the localization? Again, the logic is not sufficiently developed.

“Considering their phylogenetic relationships” The phylogenetic relationships do not seem to be related to the gene name chosen by the authors.

The gene nomenclature (STMxxx vs psrxx) varies throughout the text and figures, creating confusion and requiring frequent cross-referencing. Consistent naming should be applied throughout the manuscript.

“indicating that Salmonella harbors” the logic of this sentence is confusing and experiment on the membrane is not discussed.

“The sulfate reductases present in the membrane…” this sentence is confusing. What do the authors observe in the graphs? “induced further under anaerobiosis” is confusing (further = later or more?). “possibly reflecting transcription” What does this mean? Transcription changes induced by aerobiosis/anaerobiosis?

“The expression of psr” The authors did not test the expression, rephrase.

“harbored intermediate sulfate reductase” What about the double mutant UbiC MenA?

The colors in the figures are really confusing. The authors should either harmonize the color code (a set of color for mutants+WT,

a set for molecules) or put every graph in black and white.

“suggesting” instead of “indicating”. I do not agree with the conclusion “the sulfate reductase activity present in the …” It only suggests that the 4 proteins are sulfate reductases. The membrane localization is not tested in this experiment. I do not understand the term “initiates”. How do the authors explain the activity that is still remaining in the delta4 mutant? In this context, they should link this observation to the other sulfate reductases mentioned in the following sentences.

What do the authors mean by “distinct enzymology”? Do they mean different catalytic mechanism?

“periplasmic sulfate rely on” Fe-S cluster instead of “metal”.

Fig 3: the delta5 is never mentioned in the text.

“however, to our surprise” Why is it surprising knowing that the substrate is different?

“We noticed that Salmonella utilizes” Overinterpretation, no argumentation. Expand.

“Because H2S participates to” This statement is incorrect. Actually, FeS synthesis machineries (SUF and ISC) use cysteine as substrate. The ref 19 is outdated, several more recent reviews have been published since.

“NADH dehydrogenase” I think it contains 9 FeS, with 7 4Fe4S.

Discussion: Talking about the GOE seems far from the results.

Reviewer #3

(Remarks to the Author)
General Assessment
Kim et al. present an interesting and conceptually ambitious study demonstrating that Salmonella Typhimurium possesses a previously unappreciated metabolic flexibility in utilizing sulfide sources beyond cysteine and related metabolites. The authors identify three novel periplasmic sulfate reductases that confer oxidative stress protection and provide a fitness advantage in the streptomycin-pretreated mouse model. Mechanistic analyses suggest these enzymes support sulfate assimilation through a catalytic mechanism independent of the canonical MOCO active site, and the authors further speculate on an evolutionary link to Earth’s atmospheric history.
The work is intriguing and impactful, but the narrative currently feels uneven. Foundational concepts are introduced too briskly, several experimental rationales are unclear, and some important biological implications remain underdeveloped. The manuscript appears structured around a four-figure format. Since Nature Microbiology provides substantially more space, I encourage the authors to expand the presentation and give their very interesting data the room it deserves.
Major Comments
1. Introduction lacks essential biological context
The integration of Earth’s atmospheric history with Salmonella physiology is conceptually compelling; however, the introduction moves too quickly over foundational aspects of anaerobic respiration and sulfur assimilation. Before delving into mechanistic detail, the authors should expand on:
• The energetics of anaerobic terminal electron acceptors.
• The role of sulfur in bacterial metabolism.
• How sulfur assimilation pathways intersect with oxidative stress defences.
In addition, incorporating recent insights into Salmonella hydrogen sulfide production - particularly its roles in Fe–S cluster repair, intracellular redox balance, and stress tolerance - would strengthen the biological narrative and improve accessibility for a broad readership.
Figure 1
2. Link between oxidative stress findings and later sections is missing
The first results paragraph demonstrates that periplasmic sulfate reductases protect against oxidative stress and provide a fitness advantage in the streptomycin-pretreated mouse model. However, these findings are not revisited in the Discussion. The authors should address:
• Whether sulfur availability is limiting in the gut.
• Whether Salmonella kept these pathways to exploit alternative sulfur sources.
• The mechanistic link between sulfur metabolism and oxidative stress tolerance.
3. Clarify whether p.o. infections used streptomycin-treated mice
In the Fig. 1 infection experiments, it is not clear whether C57BL/6 mice were microbiota-perturbed with streptomycin in the main text. This should be explicitly stated.
4. Consider testing the Δ4 phenotype in additional models
As most fitness assays rely on streptomycin-pretreated mice, it would be valuable to assess the phenotype in a model that does not depend on antibiotic perturbation. Antibiotic treatment induces oxidative stress and alters luminal metabolism, potentially affecting sulfate availability.
5. Fig. 1d
What does a competitive index (CI) of ~0 indicate? Does it reflect a detection-limit issue, or is the x-axis mislabeled?
Figure 3
6. Speculation on possible undiscovered MOCO-associated activity of Psr3
The manuscript briefly hints that Psr3 may have additional, uncharacterized activity. The authors are encouraged to articulate

what functions they consider plausible.
7. Were Psr1 and Psr2 tested in a moa-deficient background?
It is unclear why the analysis focuses predominantly on Psr3. The authors should clarify whether:
• Psr1 and Psr2 were also examined in a MOCO-deficient background, and the emphasis on Psr3 is due to motif similarity with DmsA or another specific rationale?
Figure 4
8. PhsABC should be explicitly mentioned
In the text corresponding to Fig. 4f, it is unclear why PhsABC is not named in the main text.
Discussion:
The authors frame sulfur assimilation in the context of Earth's atmospheric history, yet Salmonella is fundamentally a gut-associated pathogen causing enteric disease. It would be valuable for the Discussion to address this contrast more directly - specifically, how these pathways operate and evolve within the ecological and metabolic constraints of the gut environment. And how additional sulfur assimilation pathways or oxidative stress–protection mechanisms might further promote Salmonella establishment and persistence in the gut.
Minor Comments
9. Rationale for gene prioritization needs clarification
The manuscript uses psr1, psr2, psr3, and STM14_1811 in different contexts without clearly explaining the reasoning. For example:
• Why is psr3 prioritized over psr2 in Fig. 3?
• Why is STM14_1811 introduced in Fig. 1 but not referenced again until Fig. 4?
A brief rationale at the start of each relevant section would make the work significantly easier to follow.
10. Integrate findings with recent work on sulfide-mediated competition
A recent PNAS study ("Salmonella produces sulfide to compete with Escherichia coli in the gut lumen") shows that hydrogen sulfide produced by PhsABC and AsrABC suppresses commensal E. coli, giving Salmonella a competitive edge. The authors should discuss:
• How the newly described pathways differ from PhsABC/AsrABC-mediated sulfide production,
• How these findings inform the ecological significance of sulfur metabolism in the gut.
A concise paragraph in the Discussion would suffice.

Decision Letter:

19th December 2025

*Please ensure you delete the link to your author homepage in this e-mail if you wish to forward it to your co-authors.

Dear Professor Vazquez-Torres,

Thank you for your patience while your manuscript "Molybdenum-independent [4Fe-4S]-Catalyzed Sulfate Assimilation Sustains Salmonella Virulence" was under peer-review at Nature Microbiology. It has now been seen by 3 referees, whose expertise and comments you will find at the end of this email. Although they find your work of some potential interest, they have raised a number of concerns that will need to be addressed before we can consider publication of the work in Nature Microbiology.

In particular, Reviewer #1 asks for further evidence to confirm a periplasmic location for the Psr proteins. Several reviewers ask for more insight into the proposed MOCO loading. Reviewer #2 raised significant concerns over the phylogenetic analyses, and analyses to make inferences regarding horizontal gene transfer, as well as a number of other points. These are essential to address in a revised paper as we think that these evolutionary aspects are an important element of the manuscript. Reviewer #3 asks for information to clarify whether animal experiments were done with streptomycin, has some questions about some of the CI results and asks for experiments in the absence of antibiotic treatment given that this can affect ROS and S availability in the gut environment. In general there are also a number of requests to rework the text and data presentation (with some useful guidance from Reviewer #3) towards improving the communication of the background, framing and rationale for the study, and for better communication of the data, which should be possible with the increased amount of space available within Nature Microbiology article formats compared to Nature. This is also important to address in revisions, to ensure that the findings and key conclusions are accessible also to non-specialists.

Should further experimental data allow you to address these criticisms, we would be happy to look at a revised manuscript.

We are committed to providing a fair and constructive peer-review process. Please do not hesitate to contact us if there are specific requests from the reviewers that you believe are technically impossible or unlikely to yield a meaningful outcome.

We strongly support public availability of data. Please place the data used in your paper into a public data repository, if one exists, or alternatively, present the data as Source Data or Supplementary Information. If data can only be shared on request, please explain why in your Data Availability Statement, and also in the correspondence with your editor. For some data types, deposition in a public repository is mandatory - more information on our data deposition policies and available repositories can be found at https://www.nature.com/nature-research/editorial-policies/reporting-standards#availability-of-data.

Please include a data availability statement as a separate section after Methods but before references, under the heading "Data Availability". This section should inform readers about the availability of the data used to support the conclusions of your study. This information includes accession codes to public repositories (data banks for protein, DNA or RNA sequences, microarray, proteomics data etc…), references to source data published alongside the paper, unique identifiers such as URLs to data repository entries, or data set DOIs, and any other statement about data availability. At a minimum, you should include the

following statement: “The data that support the findings of this study are available from the corresponding author upon request”, mentioning any restrictions on availability. If DOIs are provided, we also strongly encourage including these in the Reference list (authors, title, publisher (repository name), identifier, year). For more guidance on how to write this section please see: http://www.nature.com/authors/policies/data/data-availability-statements-data-citations.pdf

If revising your manuscript:

* Include a “Response to referees” document detailing, point-by-point, how you addressed each referee comment. If no action was taken to address a point, you must provide a compelling argument. This response will be sent back to the referees along with the revised manuscript.

* If you have not done so already we suggest that you begin to revise your manuscript so that it conforms to our Article format instructions at http://www.nature.com/nmicrobiol/info/final-submission. Refer also to any guidelines provided in this letter.

* Include a revised version of any required reporting checklist. It will be available to referees (and, potentially, statisticians) to aid in their evaluation if the manuscript goes back for peer review. A revised checklist is essential for re-review of the paper.

When submitting the revised version of your manuscript, please pay close attention to our href="https://www.nature.com/nature-portfolio/editorial-policies/image-integrity">Digital Image Integrity Guidelines. and to the following points below:

-- that unprocessed scans are clearly labelled and match the gels and western blots presented in figures.
-- that control panels for gels and western blots are appropriately described as loading on sample processing controls
-- all images in the paper are checked for duplication of panels and for splicing of gel lanes.

EXTENDED DATA FIGURES

When re-submitting your manuscript, please ensure that any supplementary figures and tables that are crucial to the manuscript’s conclusions are converted into Extended Data figures and tables to increase visibility of these data. Extended Data figures and tables are online-only (present in the online PDF and full-text HTML versions of the paper), peer-reviewed display items that provide essential background to the article but are not included in the main article due to space constraints. A maximum of ten Extended Data display items (figures and tables) is permitted.

Finally, please ensure that you retain unprocessed data and metadata files after publication, ideally archiving data in perpetuity, as these may be requested during the peer review and production process or after publication if any issues arise.

Please use the link below to submit a revised paper:

Link Redacted

Note: This url links to your confidential homepage and associated information about manuscripts you may have submitted or be reviewing for us. If you wish to forward this e-mail to co-authors, please delete this link to your homepage first.

Nature Microbiology is committed to improving transparency in authorship. As part of our efforts in this direction, we are now requesting that all authors identified as ‘corresponding author’ on published papers create and link their Open Researcher and Contributor Identifier (ORCID) with their account on the Manuscript Tracking System (MTS), prior to acceptance. This applies to primary research papers only. ORCID helps the scientific community achieve unambiguous attribution of all scholarly contributions. You can create and link your ORCID from the home page of the MTS by clicking on ‘Modify my Springer Nature account’. For more information please visit please visit www.springernature.com/orcid.

If you wish to submit a suitably revised manuscript we would hope to receive it within 6 months. If you cannot send it within this time, please let us know. We will be happy to consider your revision, even if a similar study has been accepted for publication at Nature Microbiology or published elsewhere (up to a maximum of 6 months).

In the meantime we hope that you find our referees' comments helpful.

Yours sincerely,

****
Reviewer Expertise:

Referee #1: DMSO reductases
Referee #2: phylogenetics, evolution
Referee #3: Salmonella infection biology

Reviewer Comments:

Reviewer #1 (Remarks to the Author):

Kim et al. report on a novel DMSO reductase paralog involved in sulfur assimilation in a strain non-typhoid Salmonella. The work involved animal and macrophage studies, genetic knock-outs, growth, peroxide, and enzyme assays, transcription, EPR spectroscopy, bioinformatics, structural modeling, and phylogenic analyses. It is a continuation of their study published in Cell Host Microbe (Kim et al., 2024) in which they demonstrate macrophage generated methionine sulfoxide as an an alternative electron acceptor in anaerobic respiration in infectious Salmonella. While they demonstrated the involvement of a DMSO reductase (DsmABC)in the previous paper, here the focus is on three additional paralogs found in the genome. They propose that one of the paralogs does so by a mechanism that is MOCO independent.

While the discovery of a MOCO independent mechanism for methionine sulfoxide reductase would be novel, the manuscript suffers from trying to cram too many ideas into a short manuscript. The figures have way too many panels, many which don't relate, and sparse information in the legends. Data is shown but not fully explained and related figures are scattered between the main text and supplemental materials. The figures need to be reorganized so that related data isn't scattered. Figure 1a, 3a,b,c,d, and 4e are not necessary.

Reading the previous publication was also helpful in figuring out the rationale for the work. The evolution aspect detracts from the paper and has little relevance to what the title eludes to. What does the GOE have to do with Salmonella virulence?

Why are the paralogs "orphans"? What evidence is there that they are periplasmic? Please identify the leader sequence that targets the periplasm (this can be added to the alignment figure). The naming convention is also incorrect. Psr is already taken (polysulfide reductase). If these paralogs are indeed periplasmic then it should be Srp.

The models in figure 2b seem out of place with the rest of the data in the figure and are way too small. One can gather from the figures and Table S2 that a great number of mutants were generated to demonstrate that MOCO is not required in the paralogs, but few are identified in the text. The results presented in the figures need to be better explained in the text. How was it determined that all the subunits were necessary? How was it determined that the EPR spectra was due to the FS0 when only membrane fractions were used and not pure protein? And what evidence suggests that "these periplasmic sulfate reductases still load MOCO...for an undiscovered activity". Modeling is suggestive and EPR of membranes is not enough. Additional structural needs to be done. Lastly, there is a recent review (2024) by Hasnat and Leimkuehler (https://doi.org/10.1016/j.bbamcr.2024.119731) "Shared functions of Fe-S cluster assembly and Moco biosynthesis" that seems relevant to the current study.

Reviewer #2 (Remarks to the Author):

This manuscript entitled “Molybdenum-independent [4Fe-4S]-catalyzed Sulfate assimilation sustains Salmonella virulence” reports the identification and characterization of novel sulfate reductases. First the authors identify 4 paralogs of DmsA in Salmonella and show that they are responsible for protection against host during infection. Then, they show that they exhibit a sulfate reductase activity and suggest that the activity relies on [Fe-S] cluster rather than MoCo. Finally, the authors show the effect of these sulfate reductases on sulfur assimilation. The analysis is well designed and the experimental results are convincing. Nevertheless, it is on several occasions hard to read, with logical gaps and assumptions that appear at a first glance unjustified. I encourage the authors to follow the rigorous scientific framework throughout the manuscript, with a clear progression of i) hypothesis ii) method iii) observation iv) interpretation and not aggregate more and more results into single conclusions. Furthermore, the phylogenetic study needs further clarifications and expanded analyses. Overall, I find the analysis itself interesting but the manuscript in its current form struggles to capture the interest, especially for a broad audience.

Major comments

1) The phylogenetic analysis suffers from some issues and needs clarification.

A) To reach the conclusions that the authors claim, they have to carry a careful analysis in a database covering the diversity of prokaryotes, and from the M&M, one can only trust the authors with “a comprehensive prokaryotic protein database”. The authors should provide the exhaustive list of genomes used, with quality parameters such as completeness and contamination percentage, and explain explicitly how this selection of genomes covers the diversity of prokaryotes.

B) I believe the authors are not specialists in phylogenetic analysis, as they appear to mix different concepts and overlook several important aspects required for robust inference. For example, the authors mix the phylogenetic inference with the dating of the phylogeny. I understand that the software infers both in a row but there are two distinct processes behind. That said, the authors should provide the phylogeny that is not dated, as for now, it is impossible to determine if the multifurcation in the dated tree is due to week support, short branches, or datation.

C) The robustness of branches should be assessed by bootstrapping.

D) The model of inference is not explicitly mentioned, a LG+G4+F is mentioned but only for the estimation of branch length during dating, which I think corresponds to the overall evolutionary model for phylogenetic inference.

E) The model used for datation is also not explicitly mentioned (strict molecular clock? Relaxed clock?). “divergence times were calibrated”: what does “clean” mean? What does “taxon sister splits” means? Internal node? What does “support by operon

context" means?

F) The characteristic of the alignment used for phylogeny should be provided (nb sequences and nb positions). Files should be provided in supplementary data (alignment, trimmed alignment, tree, outputs).

G) The sentence "The cross-species phylogenetic…" is vague and badly phrased (i.e. we do not "build" a phylogenetic analysis from queries). From what I understood, the authors used NCBI BLAST starting from 5 initial sequences, the hit resulting sequences have been used to build HMM profile using JackHMMER, used to query the local database. Rephrase and be more precise.

H) The taxonomic distribution should be presented, with presence/absence in each genome that the authors tested (Mapped on a species tree or at least in a table).

I) The authors should compare their results with that of Wells 2023.

2) The results and interpretation of phylogenetic analysis is not accurate.

A) "Molecular clock analysis placed" This is the phylogenetic tree that indicates the emergence of DmsA. I do not see any archaea in the phylogeny! "the time tree topology" Again, this is not the time tree, this is the tree topology. Overall, this sentence is not well written and the text is decorrelated to the figure. Where are the low-oxygen/sediment niches indicated? Where is Psr2? Be consistent with naming. Why underappreciated? It needs to be compared to literature.

B) "distant Proteus" This is relative, but at the scale of Bacteria, Proteus and Salmonella are very close from each other in the phylogeny (both Enterobacterales).
"indicating that this operon…" Overstatement. Every gene is retained in some species! This is the principle of natural selection and evolution. If the authors want to show that these genes are conserved, they should show the taxonomic distribution mapped on a species tree or at least in a table.

C) "The lack of synteny…":
- The figure S5 is hard to understand and the comparison between 3a and S5 is very challenging. The authors have to map the genomic context on the phylogeny (i.e. use iTOL).
- "The lack of synteny in deeper branches" Did the authors infer the genomic organization in deep branches? Otherwise rephrase.
- I do not see how a "lack of synteny" could "indicate" HGTs. The major tool to point HGTs is the phylogenetic tree and the mismatch between gene tree and species tree.
- Why to precise "over 100 million years"?
- "The group containing DmsA/Psr2A/Psr3A shares a characteristic $C3XC3XCX_nC$ FS0 architecture, whereas the ancestral Psr1A clade originating earlier in evolution bears a $C4XC3XCX_nC$" No indication on the phylogeny, hard to follow. The clade with C4XC does not branch earlier than the other clades. "ancestral Psr1A": To claim this, authors should reconstruct ancestral states by ML methods.
- "DmsA phylogeny" is it DmsA-like or DmsA phylogeny? I am confused.

D) The rest of the paragraph
This section is highly speculative, should be smoothed, and maybe placed in the discussion. The dating results should be interpreted with extreme caution for several reasons: i) The maximum-likelihood approach is not the most appropriate method for this type of analysis; a Bayesian framework is generally more suitable (and the authors do not specify which model was used). ii) The dataset includes only a single gene, which provides very limited information for reliable dating. This is consistent with the multifurcations observed. iii) The reliability of the calibration points is not discussed, further undermining the credibility of the temporal estimates.

3) A final figure with the proposed model is essential at the end of the manuscript.

Specific comments

The lack of line and page numbering is very inconvenient and makes the review process unnecessarily challenging.

"thereby enabling Salmonella to grow in gut" Hard to understand the causal link.

"biogeochemistry of Earth for over 2.5 billion years" Why so precise on the date?

"evolving prokaryotes and archaea with a panoply" bacteria instead of prokaryotes? Or eukaryota instead of archaea?

"The dmsA gene (STM14_1089) encodes a novel methionine sulfoxide reductase within the DMSO reductase superfamily, while retaining its ability to metabolize DMSO" This sentence is confusing. Mention the activity DMSO reductase first (coherent with the name), and then the novel activity. Otherwise rename the gene.

I have a doubt on the usefulness of Figure 1a. 1) the method is outdated (NJ method is no longer used) 2) the authors underuse the figure 3) A phylogenetic tree with a single genome is not particularly informative. An equivalent of Figure 3a should replace 1a. The authors could use the dataset from Wells 2023 and include salmonella sequences inside.

"indicating a series of duplication events" It could also be horizontal gene transfers.

“Construction of defined mutants” What “defined” means? Do you mean mutants of individual genes?

The gene STM14_1811 is suddenly not mentioned anymore in the text and figures, why?

STM14_1811 is mentioned in “systemic tissue than…”” but it does not appear in Fig 1d and S2a.

Why keeping the delta4 although the role of 1811 is not particularly studied compared to other genes?

In paragraph starting from “when complemented with the low…” the correspondence between plasmids for complementation and genes across text and figures is very hard to follow, use a single name for plasmids and define it in the text directly.

“mutant strains also become virulent…” Cybb is never defined. Fig 1f S3a should be at the end of the sentence. I am not specialist on this type of analysis but the statement “become virulent” seems odd for me. Indeed, the mutants are as virulent as the WT (index = 1), so they just did not lose their virulence. Why C57BL/6 is not mentioned in the text for 1f?

“Consistent with this, Salmonella used the added functions of the four…” This conclusion seems like overinterpretation. Either expand the logic behind the argument or moderate the conclusions.

“Based on the substrate specificity” What “based on” means? Is it at the level of the test that the authors used or in terms of concept? Rephrase.

Remove “duplicated” and “independently”.

“40-70%” There is no clear relationship between percentage of identity and functon(some protein families can harbor a high evolution rate and still keep the same function, and on the opposite, some can be overall very conserved in sequence but with different functions).

“encode hitherto unknown periplasmic” I did not follow why they are periplasmic, why not associated to the membrane in the cytoplasm or in the membrane? Did the authors predict the localization? Again, the logic is not sufficiently developed.

“Considering their phylogenetic relationships” The phylogenetic relationships do not seem to be related to the gene name chosen by the authors.

The gene nomenclature (STMxxx vs psrxx) varies throughout the text and figures, creating confusion and requiring frequent cross-referencing. Consistent naming should be applied throughout the manuscript.

“indicating that Salmonella harbors” the logic of this sentence is confusing and experiment on the membrane is not discussed.

“The sulfate reductases present in the membrane…” this sentence is confusing. What do the authors observe in the graphs? “induced further under anaerobiosis” is confusing (further = later or more?). “possibly reflecting transcription” What does this mean? Transcription changes induced by aerobiosis/anaerobiosis?

“The expression of psr” The authors did not test the expression, rephrase.

“harbored intermediate sulfate reductase” What about the double mutant UbiC MenA?

The colors in the figures are really confusing. The authors should either harmonize the color code (a set of color for mutants+WT, a set for molecules) or put every graph in black and white.

“suggesting” instead of “indicating”. I do not agree with the conclusion “the sulfate reductase activity present in the …” It only suggests that the 4 proteins are sulfate reductases. The membrane localization is not tested in this experiment. I do not understand the term “initiates”. How do the authors explain the activity that is still remaining in the delta4 mutant? In this context, they should link this observation to the other sulfate reductases mentioned in the following sentences.

What do the authors mean by “distinct enzymology”? Do they mean different catalytic mechanism?

“periplasmic sulfate rely on” Fe-S cluster instead of “metal”.

Fig 3: the delta5 is never mentioned in the text.

“however, to our surprise” Why is it surprising knowing that the substrate is different?

“We noticed that Salmonella utilizes” Overinterpretation, no argumentation. Expand.

“Because H2S participates to” This statement is incorrect. Actually, FeS synthesis machineries (SUF and ISC) use cysteine as substrate. The ref 19 is outdated, several more recent reviews have been published since.

“NADH dehydrogenase” I think it contains 9 FeS, with 7 4Fe4S.

Discussion: Talking about the GOE seems far from the results.

Reviewer #3 (Remarks to the Author):

General Assessment
Kim et al. present an interesting and conceptually ambitious study demonstrating that Salmonella Typhimurium possesses a previously unappreciated metabolic flexibility in utilizing sulfide sources beyond cysteine and related metabolites. The authors identify three novel periplasmic sulfate reductases that confer oxidative stress protection and provide a fitness advantage in the streptomycin-pretreated mouse model. Mechanistic analyses suggest these enzymes support sulfate assimilation through a catalytic mechanism independent of the canonical MOCO active site, and the authors further speculate on an evolutionary link to Earth's atmospheric history.
The work is intriguing and impactful, but the narrative currently feels uneven. Foundational concepts are introduced too briskly, several experimental rationales are unclear, and some important biological implications remain underdeveloped. The manuscript appears structured around a four-figure format. Since Nature Microbiology provides substantially more space, I encourage the authors to expand the presentation and give their very interesting data the room it deserves.
Major Comments
1. Introduction lacks essential biological context
The integration of Earth's atmospheric history with Salmonella physiology is conceptually compelling; however, the introduction moves too quickly over foundational aspects of anaerobic respiration and sulfur assimilation. Before delving into mechanistic detail, the authors should expand on:
• The energetics of anaerobic terminal electron acceptors.
• The role of sulfur in bacterial metabolism.
• How sulfur assimilation pathways intersect with oxidative stress defences.
In addition, incorporating recent insights into Salmonella hydrogen sulfide production - particularly its roles in Fe–S cluster repair, intracellular redox balance, and stress tolerance - would strengthen the biological narrative and improve accessibility for a broad readership.
Figure 1
2. Link between oxidative stress findings and later sections is missing
The first results paragraph demonstrates that periplasmic sulfate reductases protect against oxidative stress and provide a fitness advantage in the streptomycin-pretreated mouse model. However, these findings are not revisited in the Discussion. The authors should address:
• Whether sulfur availability is limiting in the gut.
• Whether Salmonella kept these pathways to exploit alternative sulfur sources.
• The mechanistic link between sulfur metabolism and oxidative stress tolerance.
3. Clarify whether p.o. infections used streptomycin-treated mice
In the Fig. 1 infection experiments, it is not clear whether C57BL/6 mice were microbiota-perturbed with streptomycin in the main text. This should be explicitly stated.
4. Consider testing the Δ4 phenotype in additional models
As most fitness assays rely on streptomycin-pretreated mice, it would be valuable to assess the phenotype in a model that does not depend on antibiotic perturbation. Antibiotic treatment induces oxidative stress and alters luminal metabolism, potentially affecting sulfate availability.
5. Fig. 1d
What does a competitive index (CI) of ~0 indicate? Does it reflect a detection-limit issue, or is the x-axis mislabeled?
Figure 3
6. Speculation on possible undiscovered MOCO-associated activity of Psr3
The manuscript briefly hints that Psr3 may have additional, uncharacterized activity. The authors are encouraged to articulate what functions they consider plausible.
7. Were Psr1 and Psr2 tested in a moa-deficient background?
It is unclear why the analysis focuses predominantly on Psr3. The authors should clarify whether:
• Psr1 and Psr2 were also examined in a MOCO-deficient background, and the emphasis on Psr3 is due to motif similarity with DmsA or another specific rationale?
Figure 4
8. PhsABC should be explicitly mentioned
In the text corresponding to Fig. 4f, it is unclear why PhsABC is not named in the main text.
Discussion:
The authors frame sulfur assimilation in the context of Earth's atmospheric history, yet Salmonella is fundamentally a gut-associated pathogen causing enteric disease. It would be valuable for the Discussion to address this contrast more directly - specifically, how these pathways operate and evolve within the ecological and metabolic constraints of the gut environment. And how additional sulfur assimilation pathways or oxidative stress–protection mechanisms might further promote Salmonella establishment and persistence in the gut.
Minor Comments
9. Rationale for gene prioritization needs clarification
The manuscript uses psr1, psr2, psr3, and STM14_1811 in different contexts without clearly explaining the reasoning. For example:
• Why is psr3 prioritized over psr2 in Fig. 3?
• Why is STM14_1811 introduced in Fig. 1 but not referenced again until Fig. 4?
A brief rationale at the start of each relevant section would make the work significantly easier to follow.
10. Integrate findings with recent work on sulfide-mediated competition
A recent PNAS study ("Salmonella produces sulfide to compete with Escherichia coli in the gut lumen") shows that hydrogen

sulfide produced by PhsABC and AsrABC suppresses commensal E. coli, giving Salmonella a competitive edge. The authors should discuss:
• How the newly described pathways differ from PhsABC/AsrABC-mediated sulfide production,
• How these findings inform the ecological significance of sulfur metabolism in the gut.
A concise paragraph in the Discussion would suffice.

****

Version 1:

Reviewer comments:

Reviewer #1

(Remarks to the Author)
The manuscript reads much better and addresses most if not all the reviewers' comments. A few minor corrections:

line 88: spell out "i.p." first time
line 117: replace "levels" with "cell densities"
line 139: what "reductase"? (grown Salmonella reduced reductase,)
line 169: add "periplasmic" (periplasmic nitrate reductase)
line 423: conditions
line 517: need reference for extinction coefficient as it changes at different wavelengths

reference section has italics missing for genus and species names

figure 2: percentages should be centered
figure 4: line 935 - evaluated for DMSO

Reviewer #2

(Remarks to the Author)
The manuscript by Kim et al. has undergone significant improvement and the scope of the analysis remains impressive. However, the text still does not fit with the standards expected for Nature Microbiology and would benefit from a thorough revision, including multiple corrections for clarity and precision. I still have doubts on phylogenetic analysis that require attention.

Major comments
The scientific narrative remains unclear in several sections, with missing conclusions and overly concise sentences that fail to adequately reflect the extensive work conducted. This is unfortunate, as it undermines the impact of the substantial efforts invested. While I have highlighted some specific points below, I strongly recommend a comprehensive rephrasing of the entire manuscript, possibly with the assistance of colleagues or language models, to enhance readability and coherence.

I am still not convinced by the phylogenetic analysis, particularly regarding the root used in Figure 6. The rationale for selecting the two archaeal sequences as an outgroup is not provided, yet this is a critical aspect that requires justification. The outgroup must be at least as ancient as the group of interest, otherwise, the quality of the interpretation could be severely compromised if the root is incorrect. Additionally, the authors mention introducing taxonomic distribution, but I did not find a proper distribution in sup. It is essential to provide a complete list of the studied genomes, including those in which no homologs were identified. Also, the last part of the results section is overly wordy and could be tightened, with some parts that read more like filler than analysis.

Specific comments
L53: Why use the term "founding"?
L54: Remove "significant".
L56: The term "revolution" is exaggerated. Consider a more precise term.
L76: Replace "reductase" with "reductase activity".
L76–78: There is no causal link between distinct gene clusters… and duplication/HGT events...
L89: A conclusion is missing here.
L96: A conclusion is needed.
L107–111: Justify why 1811 will no longer be studied. This seems very odd for a reader that does not know that the authors plan to study it in more detail.
L113–119: This section is confusing. Rephrase and reorganize for clarity.
L132–133: The conclusion is unclear. It would be more accurate to state that DsmA paralogs do not utilize MoCo.
L137–138: Why would it be surprising that CysIJ is expressed normally under these conditions? Clarify.
L147: As previously observed?
L164–165: And other quinones?
L184–185: These results need further development.

L189–190: This idea should precede the previous sentence for logical flow.
L190–192: This sentence condenses multiple experiments and should be expanded.
L194–195: There is no causal link between "given..." and "we examined...".
L196–198: Rephrase for better readability.
L198–200: There is no causal link between "All A, B..." and "suggesting that...".
L202: Use "determine" instead of "visualize" and "performed" instead of "pursued".
L206: The term "complemented" seems inappropriate here, as you are not restoring a phenotype.
L208–212: Expand on these results for better understanding.
L221: How can a reduction occur IN a [Fe-S] cluster? Consider rephrasing to: "[Fe-S] cluster is directly involved in sulfate reduction."
L231: "Was more toxic" is a conclusion, not an observation. Rephrase to reflect the data.
L255: How was it determined that NADH dehydrogenase activity is solely due to Nuo? Be more precise and develop.
L262: The title is unclear. What is meant by "discrete evolutionarily ordered radiation"? This terminology is ambiguous and potentially overinterpreted.
L263: How can a phylogeny "identify" something?
L270: Define "MopB_DmsA-EC".
L271–273: The claim that these niches are enriched requires justification. Was an ecological niche analysis performed? If not, clarify or remove this statement.
L274: Use "used to infer" instead of "subjected."
L286–298: This section is overly verbose and lacks focus. Consider revising for conciseness.
L299: Replace "RelTime analysis suggests" with "Dating analysis suggests."
L305: What is meant by "the crown"? This terminology is unclear and should be defined.
L311–317: This paragraph could be more impactful if compared to findings in Xsr3, particularly to assess the phylogenetic distribution of this type of Fe-S cluster.
Finally, the images in the figures are pixelated and unreadable. Higher-resolution images are necessary for clarity.

Reviewer #3

(Remarks to the Author)
The authors have thoroughly revised the manuscript and addressed all comments. I would like to congratulate them on this very interesting study.

Decision Letter:

24th April 2026

*Please ensure you delete the link to your author homepage in this e-mail if you wish to forward it to your co-authors.

Dear Professor Vazquez-Torres,

Thank you for your patience while your revised manuscript, "Molybdenum-independent [4Fe-4S]-Catalyzed Sulfate Assimilation Sustains Salmonella Virulence" was under peer-review at Nature Microbiology. It has now been seen by the original referees, whose comments you will find at the end of this email. You will see from these comments below that while they find the the revision has addressed many of their previous concerns and that the work has improved through this work, some important points are raised. We are very interested in the possibility of publishing your study in Nature Microbiology, but would like to consider your response to these concerns in the form of a revised manuscript before we make a final decision on publication.

In particular, you will see that Reviewer #2 has some remaining concerns about the phylogenetic analysis, which need to be addressed in the revised manuscript. The rest of the referees' reports are clear and the remaining issues should be straightforward to address.

We are committed to providing a fair and constructive peer-review process. Do not hesitate to contact us if there are specific requests from the reviewers that you believe are technically impossible or unlikely to yield a meaningful outcome.

If you have not done so already please begin to revise your manuscript so that it conforms to our Article format instructions at http://www.nature.com/nmicrobiol/info/final-submission/

We strongly support public availability of data. Please place the data used in your paper into a public data repository, if one exists, or alternatively, present the data as Source Data or Supplementary Information. If data can only be shared on request, please explain why in your Data Availability Statement, and also in the correspondence with your editor. For some data types, deposition in a public repository is mandatory - more information on our data deposition policies and available repositories can be found at https://www.nature.com/nature-research/editorial-policies/reporting-standards#availability-of-data.

Please include a data availability statement as a separate section after Methods but before references, under the heading "Data Availability". This section should inform readers about the availability of the data used to support the conclusions of your study. This information includes accession codes to public repositories (data banks for protein, DNA or RNA sequences, microarray, proteomics data etc…), references to source data published alongside the paper, unique identifiers such as URLs to data repository entries, or data set DOIs, and any other statement about data availability. At a minimum, you should include the following statement: "The data that support the findings of this study are available from the corresponding author upon request", mentioning any restrictions on availability. If DOIs are provided, we also strongly encourage including these in the Reference list

(authors, title, publisher (repository name), identifier, year). For more guidance on how to write this section please see: http://www.nature.com/authors/policies/data/data-availability-statements-data-citations.pdf

If your paper is accepted for publication, we will edit your display items electronically so they conform to our house style and will reproduce clearly in print. If necessary, we will re-size figures to fit single or double column width. If your figures contain several parts, the parts should form a neat rectangle when assembled. Choosing the right electronic format at this stage will speed up the processing of your paper and give the best possible results in print. We would like the figures to be supplied as vector files - EPS, PDF, AI or postscript (PS) file formats (not raster or bitmap files), preferably generated with vector-graphics software (Adobe Illustrator for example). Please try to ensure that all figures are non-flattened and fully editable. All images should be at least 300 dpi resolution (when figures are scaled to approximately the size that they are to be printed at) and in RGB colour format. Please do not submit Jpeg or flattened TIFF files. Please see also 'Guidelines for Electronic Submission of Figures' at the end of this letter for further detail.

Figure legends must provide a brief description of the figure and the symbols used, within 350 words, including definitions of any error bars employed in the figures.

When submitting the revised version of your manuscript, please pay close attention to our href="https://www.nature.com/nature-research/editorial-policies/image-integrity">Digital Image Integrity Guidelines. and to the following points below:

-- that unprocessed scans are clearly labelled and match the gels and western blots presented in figures.
-- that control panels for gels and western blots are appropriately described as loading on sample processing controls
-- all images in the paper are checked for duplication of panels and for splicing of gel lanes.

EXTENDED DATA FIGURES

When re-submitting your manuscript, please ensure that any supplementary figures and tables that are crucial to the manuscript's conclusions are converted into Extended Data figures and tables to increase visibility of these data. Extended Data figures and tables are online-only (present in the online PDF and full-text HTML versions of the paper), peer-reviewed display items that provide essential background to the article but are not included in the main article due to space constraints. A maximum of ten Extended Data display items (figures and tables) is permitted.

Finally, please ensure that you retain unprocessed data and metadata files after publication, ideally archiving data in perpetuity, as these may be requested during the peer review and production process or after publication if any issues arise.

Please include a statement before the acknowledgements naming the author to whom correspondence and requests for materials should be addressed. Please also make sure that a competing interests statement is included.

Finally, we require authors to include a statement of their individual contributions to the paper -- such as experimental work, project planning, data analysis, etc. -- immediately after the acknowledgements. The statement should be short, and refer to authors by their initials. For details please see the Authorship section of our joint Editorial policies at http://www.nature.com/authors/editorial_policies/authorship.html

When revising your paper:

* include a point-by-point response to any editorial suggestions and to our referees. Please include your response to the editorial suggestions in your cover letter, and please upload your response to the referees as a separate document.

* ensure it complies with our format requirements for Letters as set out in our guide to authors at www.nature.com/nmicrobiol/info/gta/

* state in a cover note the length of the text, methods and legends; the number of references; number and estimated final size of figures and tables

* resubmit electronically if possible using the link below to access your home page:

Link Redacted

*This url links to your confidential homepage and associated information about manuscripts you may have submitted or be reviewing for us. If you wish to forward this e-mail to co-authors, please delete this link to your homepage first.

Please ensure that all correspondence is marked with your Nature Microbiology reference number in the subject line.

Nature Microbiology is committed to improving transparency in authorship. As part of our efforts in this direction, we are now requesting that all authors identified as 'corresponding author' on published papers create and link their Open Researcher and Contributor Identifier (ORCID) with their account on the Manuscript Tracking System (MTS), prior to acceptance. This applies to primary research papers only. ORCID helps the scientific community achieve unambiguous attribution of all scholarly contributions. You can create and link your ORCID from the home page of the MTS by clicking on 'Modify my Springer Nature account'. For more information please visit please visit <a

href="http://www.springernature.com/orcid">www.springernature.com/orcid.

We hope to receive your revised paper within a month. If you cannot send it within this time, please let us know.

We look forward to hearing from you soon.

Yours sincerely,

****
Reviewers Comments:

Reviewer #1 (Remarks to the Author):

The manuscript reads much better and addresses most if not all the reviewers' comments. A few minor corrections:

line 88: spell out "i.p." first time
line 117: replace "levels" with "cell densities"
line 139: what "reductase"? (grown Salmonella reduced reductase,)
line 169: add "periplasmic" (periplasmic nitrate reductase)
line 423: conditions
line 517: need reference for extinction coefficient as it changes at different wavelengths

reference section has italics missing for genus and species names

figure 2: percentages should be centered
figure 4: line 935 - evaluated for DMSO

Reviewer #2 (Remarks to the Author):

The manuscript by Kim et al. has undergone significant improvement and the scope of the analysis remains impressive. However, the text still does not fit with the standards expected for Nature Microbiology and would benefit from a thorough revision, including multiple corrections for clarity and precision. I still have doubts on phylogenetic analysis that require attention.

Major comments
The scientific narrative remains unclear in several sections, with missing conclusions and overly concise sentences that fail to adequately reflect the extensive work conducted. This is unfortunate, as it undermines the impact of the substantial efforts invested. While I have highlighted some specific points below, I strongly recommend a comprehensive rephrasing of the entire manuscript, possibly with the assistance of colleagues or language models, to enhance readability and coherence.

I am still not convinced by the phylogenetic analysis, particularly regarding the root used in Figure 6. The rationale for selecting the two archaeal sequences as an outgroup is not provided, yet this is a critical aspect that requires justification. The outgroup must be at least as ancient as the group of interest, otherwise, the quality of the interpretation could be severely compromised if the root is incorrect. Additionally, the authors mention introducing taxonomic distribution, but I did not find a proper distribution in sup. It is essential to provide a complete list of the studied genomes, including those in which no homologs were identified. Also, the last part of the results section is overly wordy and could be tightened, with some parts that read more like filler than analysis.

Specific comments
L53: Why use the term "founding"?
L54: Remove "significant".
L56: The term "revolution" is exaggerated. Consider a more precise term.
L76: Replace "reductase" with "reductase activity".
L76–78: There is no causal link between distinct gene clusters… and duplication/HGT events...
L89: A conclusion is missing here.
L96: A conclusion is needed.
L107–111: Justify why 1811 will no longer be studied. This seems very odd for a reader that does not know that the authors plan to study it in more detail.
L113–119: This section is confusing. Rephrase and reorganize for clarity.
L132–133: The conclusion is unclear. It would be more accurate to state that DsmA paralogs do not utilize MoCo.
L137–138: Why would it be surprising that CysIJ is expressed normally under these conditions? Clarify.
L147: As previously observed?
L164–165: And other quinones?
L184–185: These results need further development.
L189–190: This idea should precede the previous sentence for logical flow.
L190–192: This sentence condenses multiple experiments and should be expanded.
L194–195: There is no causal link between "given..." and "we examined...".
L196–198: Rephrase for better readability.
L198–200: There is no causal link between "All A, B..." and "suggesting that...".

L202: Use "determine" instead of "visualize" and "performed" instead of "pursued".
L206: The term "complemented" seems inappropriate here, as you are not restoring a phenotype.
L208–212: Expand on these results for better understanding.
L221: How can a reduction occur IN a [Fe-S] cluster? Consider rephrasing to: "[Fe-S] cluster is directly involved in sulfate reduction."
L231: "Was more toxic" is a conclusion, not an observation. Rephrase to reflect the data.
L255: How was it determined that NADH dehydrogenase activity is solely due to Nuo? Be more precise and develop.
L262: The title is unclear. What is meant by "discrete evolutionarily ordered radiation"? This terminology is ambiguous and potentially overinterpreted.
L263: How can a phylogeny "identify" something?
L270: Define "MopB_DmsA-EC".
L271–273: The claim that these niches are enriched requires justification. Was an ecological niche analysis performed? If not, clarify or remove this statement.
L274: Use "used to infer" instead of "subjected."
L286–298: This section is overly verbose and lacks focus. Consider revising for conciseness.
L299: Replace "RelTime analysis suggests" with "Dating analysis suggests."
L305: What is meant by "the crown"? This terminology is unclear and should be defined.
L311–317: This paragraph could be more impactful if compared to findings in Xsr3, particularly to assess the phylogenetic distribution of this type of Fe-S cluster.
Finally, the images in the figures are pixelated and unreadable. Higher-resolution images are necessary for clarity.

Reviewer #3 (Remarks to the Author):

The authors have thoroughly revised the manuscript and addressed all comments. I would like to congratulate them on this very interesting study.

****

Version 2:

Reviewer comments:

Reviewer #2

(Remarks to the Author)
The authors followed most of my recommendations, and I am satisfied with the last version of the manuscript. A few minor comments below:

L28: “Diverse” instead of “Disparate”.
L75: Genes have no activity. Replace by protein.
L92-93: Develop this sentence. A “construction” can not “reveal”.
L153: “CysIJ” after “cytoplasmic sulfate reductase’”.
L201: The delta5 strain has not been defined before.
L222: Please replace FS0 by [Fe-S] cluster everywhere. This would be more explicit for most of readers.
L225: Why 5 DmsA paralogs? There are only 4. Do the authors mean DmsA and its 4 paralogs? Is it a different mutant from L201?
L226-228: Simplify these sentences.
L313: “Distinct” instead of “Discrete”.
L317 “clade” instead of “grade”.
L320 “grouped” instead of “ recovered”.

Decision Letter:

29th May 2026

*Please ensure you delete the link to your author homepage in this e-mail if you wish to forward it to your co-authors.

Dear Professor Vazquez-Torres,

Thank you for submitting your revised manuscript "Molybdenum-independent [4Fe-4S]-Catalyzed Sulfate Assimilation Sustains Salmonella Virulence" (NMICROBIOL-25104004B). It has now been seen by one of the original referees and their comments are below. The reviewer finds that the paper has improved in revision, and therefore we'll be happy in principle to publish it in Nature Microbiology, pending minor revisions to satisfy the referees' final requests and to comply with our editorial and formatting guidelines.

Our next steps will be to do some detailed checks of your manuscript. Before we can move forward with these checks, we need you to submit the Extended Data Figures as individual figure files [rather than in the supplementary information pdf], as done for the main figures. This is so that they can be screened alongside the main figures.

When ready, please resubmit electronically if possible using the link below to access your home page:

Link Redacted

*This url links to your confidential homepage and associated information about manuscripts you may have submitted or be reviewing for us. If you wish to forward this e-mail to co-authors, please delete this link to your homepage first.

Please ensure that all correspondence is marked with your Nature Microbiology reference number in the subject line.

Nature Microbiology is committed to improving transparency in authorship. As part of our efforts in this direction, we are now requesting that all authors identified as ‘corresponding author’ on published papers create and link their Open Researcher and Contributor Identifier (ORCID) with their account on the Manuscript Tracking System (MTS), prior to acceptance. This applies to primary research papers only. ORCID helps the scientific community achieve unambiguous attribution of all scholarly contributions. You can create and link your ORCID from the home page of the MTS by clicking on ‘Modify my Springer Nature account’. For more information please visit please visit www.springernature.com/orcid.

We hope to receive the revision within a week. If you cannot send it within this time, please let us know.

We look forward to hearing from you soon.

Yours sincerely,

****

Reviewers Comments:

Reviewer #2 (Remarks to the Author):

The authors followed most of my recommendations, and I am satisfied with the last version of the manuscript. A few minor comments below:

L28: “Diverse” instead of “Disparate”.
L75: Genes have no activity. Replace by protein.
L92-93: Develop this sentence. A “construction” can not “reveal”.
L153: “CysIJ” after “cytoplasmic sulfate reductase’”.
L201: The delta5 strain has not been defined before.
L222: Please replace FS0 by [Fe-S] cluster everywhere. This would be more explicit for most of readers.
L225: Why 5 DmsA paralogs? There are only 4. Do the authors mean DmsA and its 4 paralogs? Is it a different mutant from L201?
L226-228: Simplify these sentences.
L313: “Distinct” instead of “Discrete”.
L317 “clade” instead of “grade”.
L320 “grouped” instead of “ recovered”.

****

Version 3:

Decision Letter:

Our ref: NMICROBIOL-25104004C

8th June 2026

Dear Dr. Vazquez-Torres,

Thank you for submitting your revised manuscript "Molybdenum-independent [4Fe-4S]-Catalyzed Sulfate Assimilation Sustains Salmonella Virulence" (NMICROBIOL-25104004C).

We are now performing detailed checks on your paper and will send you a checklist detailing our editorial and formatting

requirements in about a week. Please do not upload the final materials and make any revisions until you receive this additional information from us.

Thank you again for your interest in Nature Microbiology Please do not hesitate to contact me if you have any questions.

Sincerely,

Nature Microbiology

Version 4:

Decision Letter:

6th July 2026

Dear Professor Vazquez-Torres,

I am pleased to accept your Article "Salmonella uses sulfate reductases with unique catalytic activity to promote gut colonization in mice" for publication in Nature Microbiology. Thank you for having chosen to submit your work to us and many congratulations.

Over the next few weeks, your paper will be copyedited to ensure that it conforms to Nature Microbiology style.

Once your paper is typeset, you will receive an email with a link to choose the appropriate publishing options for your paper and our Author Services team will be in touch regarding any additional information that may be required. Once your paper has been scheduled for online publication, the Nature press office will be in touch to confirm the details.

You may wish to make your media relations office aware of your accepted publication, in case they consider it appropriate to organize some internal or external publicity. Once your paper has been scheduled you will receive an email confirming the publication details. This is normally 3-4 working days in advance of publication. If you need additional notice of the date and time of publication, please let the production team know when you receive the proof of your article to ensure there is sufficient time to coordinate. Further information on our embargo policies can be found here: https://www.nature.com/authors/policies/embargo.html

After the grant of rights is completed, you will receive a link to your electronic proof via email with a request to make any corrections within 48 hours. If, when you receive your proof, you cannot meet this deadline, please inform us at rjsproduction@springernature.com immediately. You will not receive your proofs until the publishing agreement has been received through our system

Due to the importance of these deadlines, we ask you please us know now whether you will be difficult to contact over the next month. If this is the case, we ask you provide us with the contact information (email, phone and fax) of someone who will be able to check the proofs on your behalf, and who will be available to address any last-minute problems.

Acceptance of your manuscript is conditional on all authors' agreement with our publication policies (see https://www.nature.com/nmicrobiol/editorial-policies). In particular your manuscript must not be published elsewhere.

Authors may need to take specific actions to achieve compliance with funder and institutional open access mandates. If your research is supported by a funder that requires immediate open access (e.g. according to Plan S principles or the NIH public access policy) then you should select the gold OA route, and we will direct you to the compliant route where possible. Because authors warrant under our subscription licensing terms that they haven't committed to licensing any version of their article under a licence inconsistent with the terms of our agreement – including the applicable embargo period – publication under the subscription model isn't suitable for authors whose funders require no embargo.

If you have any questions about our publishing options, costs, Open Access requirements, or our legal forms, please contact ASJournals@springernature.com

An online order form for reprints of your paper is available at https://www.nature.com/reprints/author-reprints.html. All co-authors, authors' institutions and authors' funding agencies can order reprints using the form appropriate to their geographical region.

You can now use a single sign-on for all your accounts, view the status of all your manuscript submissions and reviews, access usage statistics for your published articles and download a record of your refereeing activity for the Nature journals.

To assist our authors in disseminating their research to the broader community, our SharedIt initiative provides you with a unique

shareable link that will allow anyone (with or without a subscription) to read the published article. Recipients of the link with a subscription will also be able to download and print the PDF.

As soon as your article is published, you will receive an automated email with your shareable link.

With kind regards,

P.S. Click on the following link if you would like to recommend Nature Microbiology to your librarian http://www.nature.com/subscriptions/recommend.html#forms

** Visit the Springer Nature Editorial and Publishing website at www.springernature.com/editorial-and-publishing-jobs for more information about our career opportunities. If you have any questions please click here.**

Open Access This Peer Review File is licensed under a Creative Commons Attribution 4.0 International License, which permits use, sharing, adaptation, distribution and reproduction in any medium or format, as long as you give appropriate credit to the original author(s) and the source, provide a link to the Creative Commons license, and indicate if changes were made.
In cases where reviewers are anonymous, credit should be given to 'Anonymous Referee' and the source.
The images or other third party material in this Peer Review File are included in the article's Creative Commons license, unless indicated otherwise in a credit line to the material. If material is not included in the article's Creative Commons license and your intended use is not permitted by statutory regulation or exceeds the permitted use, you will need to obtain permission directly from the copyright holder.
To view a copy of this license, visit https://creativecommons.org/licenses/by/4.0/

Reviewer #1 (Remarks to the Author):

Kim et al. report on a novel DMSO reductase paralog involved in sulfur assimilation in a strain non-typhoid Salmonella. The work involved animal and macrophage studies, genetic knock-outs, growth, peroxide, and enzyme assays, transcription, EPR spectroscopy, bioinformatics, structural modeling, and phylogenic analyses. It is a continuation of their study published in Cell Host Microbe (Kim et al., 2024) in which they demonstrate macrophage generated methionine sulfoxide as an an alternative electron acceptor in anaerobic respiration in infectious Salmonella. While they demonstrated the involvement of a DMSO reductase (DsmABC)in the previous paper, here the focus is on three additional paralogs found in the genome. They propose that one of the paralogs does so by a mechanism that is MOCO independent.

While the discovery of a MOCO independent mechanism for methionine sulfoxide reductase would be novel, the manuscript suffers from trying to cram too many ideas into a short manuscript. The figures have way too many panels, many which don't relate, and sparse information in the legends. Data is shown but not fully explained, and related figures are scattered between the main text and supplemental materials. The figures need to be reorganized so that related data isn't scattered. Figure 1a, 3a,b,c,d, and 4e are not necessary.

Response: As suggested, we have separated figures, data have been reorganized, and **Fig 3c**, **and 3d** have been erased. We have kept **Fig 1a** because it identifies the five *dmsA* paralogs encoded in the *Salmonella* genome. We prefer to show **3a** and **3b** (now **Fig 6a** and **6b**) as they show the distribution, phylogenetic relationships and calibrated ages of the *xsr* genes across bacteria. Having said that, **Fig 6a** and **6b** have been extensively modified in response to Reviewer #2's comments. We also prefer to keep **Fig 4e** as it shows the context in which each of these pathways synthesizes H_2S.

Reading the previous publication was also helpful in figuring out the rationale for the work. The evolution aspect detracts from the paper and has little relevance to what the title eludes to. What does the GOE have to do with Salmonella virulence?

Response: The evolutionary aspect of the project is important for understanding the diversification of a family of enzymes widely used across all domains of life, including 17 times in the *Salmonella* genome. Our new phylogenetic analyses indicate that Xsr3ABC, the closest relative of the DmsABC complex, arose during the GOE. The accumulation of O_2, Mo, sulfur, and novel terminal electron acceptors during the GOE drove the spectacular diversification of DMSO reductase family members. Our revised timeclock analyses indicate that Xsr1ABC and Xsr2ABC complexes predate the GOE, and are more widely distributed among Proteobacteria, Actinobacteria and Firmicutes. We hope that the extensive revision of the evolutionary relations of *xsr* genes clarifies why we deem important to present the evolutionary aspects of widely distributed *xsr* genes.

Why are the paralogs "orphans"? What evidence is there that they are periplasmic? Please identify the leader sequence that targets the periplasm (this can be added to the alignment figure). The naming convention is also incorrect. Psr is already taken (polysulfide reductase). If these paralogs are indeed periplasmic then it should be Srp.

Response: We have replaced "orphan" for “open reading frames of unknown function.” The evidence that these proteins are periplasmic is based on similarity with DMSO reductase family members, and membrane localization. Moreover, the RR (twin-arginine) signatures and cleavage sites in the N-terminus of STM14_3103, STM14_5178 and STM14_1809 are consistent with the idea that these oxidoreductases are substrates of the TAT secretion system. We now provide experimental evidence that shows that sulfate reductase activity is completely lost in membranes isolated from a Δ*tat* mutant. In addition to the TAT system, dedicated chaperones aid with the

interactions of substrates with the TAT system. Deletion of the *STM14_1806* gene encoding the DmsD chaperone within the *xsr* operon ablated the sulfate reductase activity of the *xsr3*+ strain, further suggesting that Xsr3 is periplasmic. Collectively, the new bioinformatic, genetic and biochemical analyses support the periplasmic localization of the sulfate reductases described during our investigations (**Fig 3**). We prefer not using *srp*, because this term has previously been used to identify signal recognition particles in bacteria. The sulfate reductases described here may also be present in organisms that lack a periplasm. Therefore, we have decided to call these genes *xsr* (extracytoplasmic sulfate reductase).

The models in figure 2b seem out of place with the rest of the data in the figure and are way too small. One can gather from the figures and Table S2 that a great number of mutants were generated to demonstrate that MOCO is not required in the paralogs, but few are identified in the text. The results presented in the figures need to be better explained in the text. How was it determined that all the subunits were necessary? How was it determined that the EPR spectra was due to the FS0 when only membrane fractions were used and not pure protein? And what evidence suggests that "these periplasmic sulfate reductases still load MOCO...for an undiscovered activity". Modeling is suggestive and EPR of membranes is not enough. Additional structural needs to be done. Lastly, there is a recent review (2024) by Hasnat and Leimkuehler (https://doi.org/10.1016/j.bbamcr.2024.119731) "Shared functions of Fe-S cluster assembly and Moco biosynthesis" that seems relevant to the current study.

Response: As requested, we have reorganized the data in **Fig 2**, enlarging **panel b**. We have also identified all mutations in the main text and expanded our explanations of the data more fully. Genetic, microbiological, and biochemical approaches demonstrate that all subunits of the complex are necessary for the sulfate reducing activity of the Xsr3 complex. To test the importance of the three subunits in the catalytic activity of Xsr3, a Δ5 mutant lacking all *dmsA* paralogs was complemented with a low copy plasmid bearing A, B, C, AB, AC, BC, or ABC subunits of the STM14_1809-07 operon. Only the construct expressing the ABC subunits supported sulfate reductase activity in the Δ5 mutant. These data, which have been moved from supplementary file to **Fig 4d** and **Fig S5e**, demonstrate that all A, B, and C subunits are necessary for sulfate reductase activity of the Xsr3 paralog.

To study the EPR signatures of specific DMSO reductase paralogs, EPR data were generated from membranes obtained from a mutant deleted for STM14_1811, *xsr1*, *xsr2*, and *xsr3* operons. Complementation of the resulting Δ*5* mutant with a plasmid harboring STM14_1809-1807 genes generated an EPR signature consistent with previously described [4Fe-4S] clusters. The EPR signal disappeared in membrane preparations from a strain bearing mutations in Asn^{66} or Arg^{105} residues that help coordinate the FS0 cluster (these mutations were selected based on previous characterization of *dmsA* FS0 cluster (references PMID: 21357619 and PMID: 8125918). Moreover, the addition of sulfate eliminated the EPR signal, consistent with oxidation of the [4Fe-4S] cluster during sulfate reduction.

We don’t have any evidence to show that the MOCO cofactor is loaded into the complex. For clarity, we have eliminated this comment from the revised manuscript.

As requested, we have now purified DmsABC and Xsr1ABC complexes from *Salmonella* cultures to high purity (**Fig S5c**). Consistent with our original findings, purified DmsABC complexes reduced DMSO in a reconstituted biochemical system, an activity that was lost in specimens containing the DmsA S199G variant (**Fig S5d**). The serine residue provides the pentacoordination of the catalytic Mo that is necessary for DMSO reductase activity of DmsA. This new information is consistent with the notion that reduction of DMSO by DmsABC relies on the MOCO cofactor. In sharp contrast, purified complexes containing wild-type Xsr3A or the Xsr3A S199A variant supported sulfate reduction in an *in vitro* reconstituted biochemical system. Consistent with our original findings, the new information generated with the purified Xsr3ABC complex demonstrates that sulfate reduction is independent of the penta-coordinating serine. In

other words, the canonical MOCO cofactor is dispensable for the Xsr3-catalyzed sulfate reduction. This finding is consistent with the excellent sulfate reductase activity of *xsr1*$^+$, *xsr*2$^+$*, or xsr3*$^+$ strains deficient in the *moa* operon (new experiments shown in **Fig 4c**), whose products are responsible for the synthesis and the loading of the MOCO cofactor into the catalytic A subunit.

As suggested, we have added the review by Hasnat and Leimkuehler to the revised document.

Reviewer #2 (Remarks to the Author):

This manuscript entitled "Molybdenum-independent [4Fe-4S]-catalyzed Sulfate assimilation sustains Salmonella virulence" reports the identification and characterization of novel sulfate reductases. First the authors identify 4 paralogs of DmsA in Salmonella and show that they are responsible for protection against host during infection. Then, they show that they exhibit a sulfate reductase activity and suggest that the activity relies on [Fe-S] cluster rather than MoCo. Finally, the authors show the effect of these sulfate reductases on sulfur assimilation. The analysis is well designed and the experimental results are convincing.

Response: Thank you.

Nevertheless, it is on several occasions hard to read, with logical gaps and assumptions that appear at a first glance unjustified. I encourage the authors to follow the rigorous scientific framework throughout the manuscript, with a clear progression of i) hypothesis ii) method iii) observation iv) interpretation and not aggregate more and more results into single conclusions. Furthermore, the phylogenetic study needs further clarifications and expanded analyses. Overall, I find the analysis itself interesting but the manuscript in its current form struggles to capture the interest, especially for a broad audience.

Response: This issue was raised by all three reviewers. Hopefully, the revised and expanded document will make it an easier read.

Major comments

1) The phylogenetic analysis suffers from some issues and needs clarification.

A) To reach the conclusions that the authors claim, they have to carry a careful analysis in a database covering the diversity of prokaryotes, and from the M&M, one can only trust the authors with "a comprehensive prokaryotic protein database". The authors should provide the exhaustive list of genomes used, with quality parameters such as completeness and contamination percentage, and explain explicitly how this selection of genomes covers the diversity of prokaryotes.

Response: Sorry this point did not come across more clearly in the original submission. We have incorporated the suggestions of the reviewer to increase the robustness of the phylogenetic analyses. The phylogenetic analyses originate from a domain-informed JackHMMER search against UniProt, yielding ~250,000 homologous sequences spanning broad prokaryotic diversity. This dataset was first filtered for conserved molybdopterin-binding and [Fe–S] cluster–associated domains that are characteristic of DmsA-like molybdoenzymes, yielding ~29,000 hits. The initial hits were subject to redundancy reduction using MMseqs2, narrowing the number of hits to ~9000. Deprecated UniProt entries were then removed. The resulting phylogenetic dataset (~3000 hits) spans representatives from all major prokaryotic lineages in which DmsA-like systems are known or predicted to occur, including Actinobacteria, Firmicutes, and multiple classes of Proteobacteria. This ensured broad phylogenetic coverage while maintaining functional coherence. A phylogenetic tree was constructed from this dataset together with 17 *Salmonella* MopB-family proteins, and clades clustering with *bona fide* DmsA homologs were identified. Upon examination of the genomic context and synteny, orphan DmsA-like

proteins were excluded from subsequent analyses. Table S5 lists the genomes included in the final phylogenetic analysis, along with accession numbers. Moreover, the expanded Methods section explicitly describes genome selection and diversity coverage.

B) I believe the authors are not specialists in phylogenetic analysis, as they appear to mix different concepts and overlook several important aspects required for robust inference. For example, the authors mix the phylogenetic inference with the dating of the phylogeny. I understand that the software infers both in a row but there are two distinct processes behind. That said, the authors should provide the phylogeny that is not dated, as for now, it is impossible to determine if the multifurcation in the dated tree is due to week support, short branches, or datation.

Response: We agree with the Reviewer that phylogenetic inference and temporal dating are conceptually distinct steps. In our analysis, phylogenetic inference was performed first using a maximum-likelihood framework to estimate tree topology and branch lengths. Temporal dating was applied later to this fixed topology, using the inferred branch lengths as input. We agree that this distinction was not made sufficiently explicit in the original manuscript. To address this concern, we have now included the undated maximum-likelihood phylogeny (iqtree UFBoot 1000 output) and the pruned phylogeny as a main figure (**Fig 6a**). We have revised the Methods and Results sections to explicitly distinguish between phylogenetic inference and temporal dating. We have also clarified that all biological interpretations are based on the inferred topology, which is consistent between the dated and undated trees.

C) The robustness of branches should be assessed by bootstrapping.

Response: As suggested, the robustness of branches has been assessed by bootstrapping (shown as grey spheres at respective nodes).

D) The model of inference is not explicitly mentioned, a LG+G4+F is mentioned but only for the estimation of branch length during dating, which I think corresponds to the overall evolutionary model for phylogenetic inference.

Response: Phylogenetic inference was performed using a maximum-likelihood framework, with the best-fitting amino acid substitution model selected using ModelFinder. For the final dataset, according to the Bayesian Information Criterion (BIC), the LG+F+R6 was identified as the best-fit model and used for inference of tree topology and branch lengths in substitutions per site. This information has been clarified in Materials and Methods and the analysis shown in **Fig 6, S8, and S9**.

E) The model used for datation is also not explicitly mentioned (strict molecular clock? Relaxed clock?). “divergence times were calibrated”: what does “clean” mean? What does “taxon sister splits” means? Internal node? What does “support by operon context” means?

Response: Divergence times were estimated via RelTime-ML (Tamura et al. 2018, PMID: 29893954) in MEGA X, a relaxed clock approach that infers lineage-specific relative rates (without assuming a strict global clock) before scaling branch lengths to absolute time (Ga) using calibration constraints. In our updated time tree, we used three geological calibration points with soft bound priors. This non-strict clock outperforms global rate assumptions on rate-heterogeneous bacterial datasets. Nodal age estimates in relative clock are reported with 95% CIs as supplementary **Fig S10**. We have revised the Methods to more accurately described the procedures.

F) The characteristic of the alignment used for phylogeny should be provided (nb sequences

and nb positions). Files should be provided in supplementary data (alignment, trimmed alignment, tree, outputs).

Response: As requested, we have now added explicit information on the number of sequences and alignment length used for phylogenetic inference as Supplementary Data file. In addition, we have deposited the multiple sequence alignment, trimmed alignment, maximum-likelihood tree, time-scaled tree, and relevant software output files in the Supplementary Data file. These materials will allow full reproducibility of the phylogenetic and time-tree analyses.

G) The sentence "The cross-species phylogenetic…" is vague and badly phrased (i.e. we do not "build" a phylogenetic analysis from queries). From what I understood, the authors used NCBI BLAST starting from 5 initial sequences, the hit resulting sequences have been used to build HMM profile using JackHMMER, used to query the local database. Rephrase and be more precise.

Response: We thank the reviewer for pointing out this imprecise wording. We did not build phylogenetic analysis directly from sequence queries, nor did we rely on a simple BLAST-based approach. Instead, the main DmsA paralog from *Salmonella enterica* serovar Typhimurium was used as seed sequence to initiate a large-scale homology search using JackHMMER against the UniProt database. This search yielded a comprehensive set of DmsA-like homologs across prokaryotes, which was subsequently refined through domain-based filtering, redundancy reduction, and phylogenetic clustering prior to tree inference. We have revised the sentence accordingly to accurately and explicitly describe this workflow (**Fig S8a**).

H) The taxonomic distribution should be presented, with presence/absence in each genome that the authors tested (Mapped on a species tree or at least in a table).

Response: We agree with the Reviewer that explicitly presenting the taxonomic distribution improves clarity and interpretability. As requested, the taxonomic distribution of the analyzed genomes is now presented in **Table S5**. Also, we annotated all the phylogenetic trees in the revised manuscript at phylum and class levels.

I) The authors should compare their results with that of Wells 2023.

Response: Thank you for this important suggestion. We have now explicitly compared our results to both Wells et al., 2020 (PMID: 32616801) and Wells et al., 2023(PMID: 36951557) in the Results and Discussion sections. Our phylogeny (9,462 → 80 sequences) confirms Wells et al., 2023 DmsA/NarG respiratory clade position with 6× greater sampling and calibrated timetree dates. Wells' 2023 data from 47k+ MopB superfamily analysis emphasizes cofactor loss (×3 independent events) and domain shuffling across the structural fold. Our JackHMMER search using Mi-bisPGD+FeS domains retrieved only cofactor-present catalytic subunits, complementing Wells' fold-based expansion by resolving finer subclade structure within the respiratory DmsA/NarG lineage.

2) The results and interpretation of phylogenetic analysis is not accurate.

A) "Molecular clock analysis placed" This is the phylogenetic tree that indicates the emergence of DmsA. I do not see any archaea in the phylogeny! "the time tree topology" Again, this is not the time tree, this is the tree topology. Overall, this sentence is not well written and the text is decorrelated to the figure. Where are the low-oxygen/sediment niches indicated? Where is Psr2? Be consistent with naming. Why underappreciated? It needs to be compared to literature.

Response: In response to the Reviewer's comment, we have clarified that the time-tree analysis infers relative divergence patterns within bacteria. We had labelled archaeal homologs with their genus names in the current phylogeny. We have also explained the usage of the *xsr* terminology. Finally, we have replaced the subjective term "underappreciated" with more precise

language and added appropriate literature references to contextualize the contribution of this work relative to previous studies.

B) “distant Proteus” This is relative, but at the scale of Bacteria, Proteus and Salmonella are very close from each other in the phylogeny (both Enterobacterales). “indicating that this operon…” Overstatement. Every gene is retained in some species! This is the principle of natural selection and evolution. If the authors want to show that these genes are conserved, they should show the taxonomic distribution mapped on a species tree or at least in a table.

Response: As requested, we have revised the text to remove the term “distant”. We have also softened language implying operon “retention” and now describe the observed distribution more cautiously, emphasizing that the DmsABC operon is repeatedly observed across multiple related and distinct bacterial lineages.

C) “The lack of synteny…”:

- The figure S5 is hard to understand and the comparison between 3a and S5 is very challenging. The authors have to map the genomic context on the phylogeny (i.e. use iTOL).

Response: As requested, we have now mapped genomic context directly onto the phylogenetic tree using iTOL. Specifically, operon organization (DmsABC present versus disrupted synteny), paralog class (DmsA, Xsr1A, Xsr2A), and FS0 motif type are now annotated alongside the phylogeny in **Fig 6** and **Fig S8**, **S9** and **S11**.

- “The lack of synteny in deeper branches” Did the authors infer the genomic organization in deep branches? Otherwise rephrase.

Response: We have eliminated the synteny analysis of the revised manuscript. This aspect deserves significant development that will be presented in a future paper.

- I do not see how a “lack of synteny” could “indicate” HGTs. The major tool to point HGTs is the phylogenetic tree and the mismatch between gene tree and species tree.

Response: As mentioned above, synteny is not considered in the revised manuscript.

- Why to precise “over 100 million years”?

Response: We agree with Reviewer #2 that specifying an absolute timescale may not be justified. The time-tree analysis performed here employed the RelTime method that infers relative divergence patterns and does not provide absolute ages in the absence of external constraints. We have therefore removed the reference to “over 100 million years” and any other exact ages and revised the text to describe these events in relative terms, referring instead to evolutionary time periods.

- “The group containing DmsA/Psr2A/Psr3A shares a characteristic C3XC3XCX$_n$C FS0 architecture, whereas the ancestral Psr1A clade originating earlier in evolution bears a C4XC3XCX$_n$C” No indication on the phylogeny, hard to follow. The clade with C4XC does not branch earlier than the other clades. “ancestral Psr1A”: To claim this, authors should reconstruct ancestral states by ML methods.

Response: In response to the Reviewer’s comment, we have downplayed the evolutionary directionality. While the phylogeny resolves relationships among DmsA/Xsr paralogs, it does not by itself justify inference of ancestral states without explicit ancestral state reconstruction analysis. We have therefore removed the term “ancestral” and revised the text to describe the observed distribution of FS0 motif types across clades without implying evolutionary polarity.

- “DmsA phylogeny” is it DmsA-like or DmsA phylogeny? I am confused.

Response: Throughout the manuscript, we use the term 'DmsA-like' to refer to homologous catalytic subunits of the DMSO reductase superfamily, including *bona fide* DmsA as well as related Xsr-type paralogs.

D) The rest of the paragraph
This section is highly speculative, should be smoothed, and maybe placed in the discussion. The dating results should be interpreted with extreme caution for several reasons: i) The maximum-likelihood approach is not the most appropriate method for this type of analysis; a Bayesian framework is generally more suitable (and the authors do not specify which model was used). ii) The dataset includes only a single gene, which provides very limited information for reliable dating. This is consistent with the multifurcations observed. iii) The reliability of the calibration points is not discussed, further undermining the credibility of the temporal estimates.

Response: We have revised the manuscript to substantially soften the more speculative interpretations of the time-tree analysis. We agree that single-gene time-trees provide limited temporal resolution. Accordingly, we base all primary conclusions on the well-supported maximum-likelihood phylogenetic topology rather than on absolute age estimates. While fully Bayesian relaxed-clock frameworks are often preferred for divergence dating, our objective was not to obtain precise absolute divergence times, but rather to examine relative temporal ordering of major diversification events within the DmsA-like family. The RelTime approach accommodates lineage-specific rate variation without assuming a strict global clock and provides an efficient framework for exploring relative divergence structure in single-locus datasets. Because reliable fossil calibrations are unavailable for this gene family, soft secondary constraints derived from bacterial phylogenomic chronograms were implemented conservatively to avoid overconstraining node ages. Specifically, the archaeal–bacterial divergence was constrained to 3.8–3.0 Ga, Actinomycetota diversification to 2.0–1.2 Ga, and Gammaproteobacterial radiation to 0.8–0.5 Ga, with all bounds consistent with divergence time estimates retrieved from the TimeTree5 database (PMID: 35932227). We have also explicitly evaluated the robustness of the time-tree results using varying models in the RelTime relative clock model (**Fig S10**). While absolute node ages vary under these perturbations, the relative temporal ordering of major diversification events and the inferred sequence of paralog radiations remain unchanged. We now explicitly state in the manuscript that these temporal estimates should be interpreted with caution and are intended to explore relative evolutionary patterns rather than to provide precise chronometric conclusions (lines 314-316).

3) A final figure with the proposed model is essential at the end of the manuscript.

Response: a model figure has been added to the revised manuscript.

Specific comments

The lack of line and page numbering is very inconvenient and makes the review process unnecessarily challenging.

Response: As requested, the revised document includes line and page numbering.

"thereby enabling Salmonella to grow in gut" Hard to understand the causal link.

Response: We have softened the causal link. The sentence now reads: "The three extracytoplasmic sulfate reductases facilitate the formation of hydrogen sulfide and [Fe-S] metalloproteins, enable the growth of *Salmonella* in gut and viscera, and defend against reactive species engendered by the phagocyte NADPH oxidase."

"biogeochemistry of Earth for over 2.5 billion years" Why so precise on the date?

Response: We have replaced the reference to “over 2.5 billion years” with more general contextual language referring to major redox transitions, including the Great Oxidation Event, without implying direct temporal placement or causality.

“evolving prokaryotes and archaea with a panoply” bacteria instead of prokaryotes? Or eukaryota instead of archaea?

Response: The changes in wording have been made.

“The dmsA gene (STM14_1089) encodes a novel methionine sulfoxide reductase within the DMSO reductase superfamily, while retaining its ability to metabolize DMSO” This sentence is confusing. Mention the activity DMSO reductase first (coherent with the name), and then the novel activity. Otherwise rename the gene.

Response: The suggested change in order has been made.

I have a doubt on the usefulness of Figure 1a. 1) the method is outdated (NJ method is no longer used) 2) the authors underuse the figure 3) A phylogenetic tree with a single genome is not particularly informative. An equivalent of Figure 3a should replace 1a. The authors could use the dataset from Wells 2023 and include salmonella sequences inside.

Response: We appreciate the reviewer’s concern and realize that the distinction between intra-genomic clustering (**Fig 1a**) and cross-species phylogenetic inference (**Fig 3a**) was not sufficiently explicit in the original manuscript. We would like to clarify that Fig. 1a is not intended as a phylogenetic inference, but rather as a descriptive analysis illustrating the diversity and organization of MopB-family paralogs within a single *Salmonella* genome. The Neighbor-Joining tree in **Fig 1a** is used solely for visualization of intra-genomic relationships and to highlight the subset of five closely related DmsA-like paralogs that motivated subsequent functional and evolutionary analyses. To complement the tree-based visualization of **Fig 1a**, we also analyzed pairwise sequence similarities among the 17 MopB-family proteins using hierarchical clustering (**Fig S1a**).

“indicating a series of duplication events“ It could also be horizontal gene transfers.

Response: The sentence has been modified to entertain the possibility of duplication or HGT.

“Construction of defined mutants” What “defined” means? Do you mean mutants of individual genes?

Response: By defined mutants, we mean sequence-confirmed mutants resulting from λ-Red recombination.

The gene STM14_1811 is suddenly not mentioned anymore in the text and figures, why?

Response: At the time of submission, we did not know the function of the STM14_1811 gene product. We now know that gene STM14_1811 encodes a selenate reductase. A manuscript describing STM14_1811 is underway.

STM14_1811 is mentioned in “systemic tissue than…”' but it does not appear in Fig 1d and S2a.

Response: As discussed above, the functional characterization of STM14_1811 will be the subject of a separate paper.

Why keeping the delta4 although the role of 1811 is not particularly studied compared to other genes?

Response: When we initiated the investigations described in this manuscript, we thought the STM14_1811 gene, given its proximity, upstream localization and lack of BC subunits, would be related to the STM14_1809 gene. We now know STM14_1811 encodes a selenate reductase

that can either use BC complexes from 1808-1807 or the *dmsABC* operon. As mentioned above, this will be the subject of a future publication.

In paragraph starting from "when complemented with the low…" the correspondence between plasmids for complementation and genes across text and figures is very hard to follow, use a single name for plasmids and define it in the text directly.

Response: As suggested, we have clarified in **Table S3** the genes expressed by each of the plasmids. **Table S2** shows the relevant genomic characteristics of the strains used, including complementing plasmids.

"mutant strains also become virulent…" Cybb is never defined. Fig 1f S3a should be at the end of the sentence. I am not specialist on this type of analysis but the statement "become virulent" seems odd for me. Indeed, the mutants are as virulent as the WT (index = 1), so they just did not lose their virulence. Why C57BL/6 is not mentioned in the text for 1f?

Response: *Cybb* is defined at the end of the sentence (verbal description and citation of work where this mouse strain was first published). We now specify that the *Cybb* gene encodes the gp91phox subunit of the phagocyte NADPH oxidase. We prefer to cite **Fig 1e** and **S2d** in their current location because they direct the reader to the data specific to mice and macrophages, respectively. For clarity, the data corresponding to **Fig 1f** is now presented in a separate sentence. The Reviewer is correct that the mutants are not different in macrophages and mice bearing the *Cybb*$^{-/-}$ deletion. This statement must be put in context with the attenuation of the *xsr* mutants in wild-type C57BL/6 mice. Therefore, the claim that *xsr* mutants become virulent in *Cybb*$^{-/-}$ mice and *Cybb*$^{-/-}$ macrophages is accurate.

"Consistent with this, Salmonella used the added functions of the four…" This conclusion seems like overinterpretation. Either expand the logic behind the argument or moderate the conclusions.

Response: The text now reads "The Δ*4* strain was more susceptible to bactericidal concentrations of hydrogen peroxide (H_2O_2) than any of the single *dmsA* paralog mutants (**Fig 1g).** Single *dmsA* paralog mutants complemented with the low copy pWSK29 plasmid expressing STM14-1809, STM14_3103 or STM14_5178 operons were as susceptible to H_2O_2 killing as wild-type *Salmonella* **(Fig S3b).** *Salmonella* strains expressing single *dmsA* paralogs (e.g., *1809*$^{+}$) were also hypersusceptible to the bacteriostatic activity of H_2O_2 **(Fig S3c**)."

"Based on the substrate specificity" What "based on" means? Is it at the level of the test that the authors used or in terms of concept? Rephrase.

Response: The text has been rephrased to "Based on the substrates used by DmsABC, we tested whether the DmsA paralogs reduce methionine sulfoxide or DMSO."

Remove "duplicated" and "independently".

Response: Done.

"40-70%" There is no clear relationship between percentage of identity and functon(some protein families can harbor a high evolution rate and still keep the same function, and on the opposite, some can be overall very conserved in sequence but with different functions).

Response: As requested, the revised text omits interpretation.

"encode hitherto unknown periplasmic" I did not follow why they are periplasmic, why not associated to the membrane in the cytoplasm or in the membrane? Did the authors predict the localization? Again, the logic is not sufficiently developed.

Response: Please, see above in our response to Reviewer #1 for a complete response to this important question. Briefly, bioinformatics, genetic analyses of TAT and *dmsD* chaperone

mutants, and membrane localization support the idea that the *xsr* gene products operate in the periplasm.

“Considering their phylogenetic relationships” The phylogenetic relationships do not seem to be related to the gene name chosen by the authors.

Response: The text has been edited following the Reviewer’s recommendations. Please, see text in lines 173-178.

The gene nomenclature (STMxxx vs psrxx) varies throughout the text and figures, creating confusion and requiring frequent cross-referencing. Consistent naming should be applied throughout the manuscript.

Response: STM nomenclature is used until our experiments demonstrate that the loci in question encode sulfate reductases, at which time we use the *xsr* nomenclature (lines 176-178).

“indicating that Salmonella harbors” the logic of this sentence is confusing and experiment on the membrane is not discussed.

Response: We have rephrased the sentence which now reads “Cytoplasmic extracts isolated from Δ*4 Salmonella* and wild-type *Salmonella* exhibited comparable sulfate reductase activity (**Fig 3b**), indicating that the cytoplasmic sulfate reductase is expressed normally is the Δ*4 Salmonella* strain.”

“The sulfate reductases present in the membrane…” this sentence is confusing. What do the authors observe in the graphs? “induced further under anaerobiosis” is confusing (further = later or more?). “possibly reflecting transcription” What does this mean? Transcription changes induced by aerobiosis/anaerobiosis?

Response: We have changed the text to: “Membranes of aerobically and anaerobically grown *Salmonella* reduced sulfate, with the latter exhibiting higher activity (**Fig 3c**). The increased sulfate reductase activity present in membranes isolated from anaerobically grown *Salmonella* may reflect the induction of *STM14_1809*, *STM14_3103*, and *STM14_5178* genes (**Fig S4b**).”

“The expression of psr” The authors did not test the expression, rephrase.

Response: We have modified the text to more accurately reflect our rationale.

“harbored intermediate sulfate reductase” What about the double mutant UbiC MenA?

Response: A double mutant was not tested because the growth of the *ubiA*$^{-}$ *menA*$^{-}$ strain in *E. coli* is severely compromised (PMID: 195602). Importantly, the addition of either ubiquinone or menaquinone supported sulfate reductase activity in the membrane fractions. These experiments demonstrate that the Xsr3 sulfate reductase can use both menaquinone and ubiquinone, a situation that has previously been reported for nitrate reductase (PMID: 11967083).

The colors in the figures are really confusing. The authors should either harmonize the color code (a set of color for mutants+WT, a set for molecules) or put every graph in black and white.

Response: We have tried to consistently use a color identifier for each strain. Given the number of strains used in these investigations, this can be a difficult task.

“suggesting” instead of “indicating”. I do not agree with the conclusion “the sulfate reductase activity present in the …” It only suggests that the 4 proteins are sulfate reductases. The membrane localization is not tested in this experiment. I do not understand the term “initiates”. How do the authors explain the activity that is still remaining in the delta4 mutant? In this context,

they should link this observation to the other sulfate reductases mentioned in the following sentences.
Response: As indicated, the revised manuscript uses “suggesting.”

What do the authors mean by “distinct enzymology”? Do they mean different catalytic mechanism?

Response: The ATP-independent activity of the sulfate reductases described here indicate different catalytic mechanism from the CysIJ system. As suggested by the reviewer, the text now reads “The enzymology of the sulfate reductase present in the membrane appears, however, to involve a catalytic mechanism that is different from the cytoplasmic CysIJ pathway,…”

“periplasmic sulfate rely on” Fe-S cluster instead of “metal”.

Response: We have made the change as suggested by the reviewer.

Fig 3: the delta5 is never mentioned in the text.

Response: Sorry for the omission. The Δ*5* strain is now mentioned in the text (see line 206).

“however, to our surprise” Why is it surprising knowing that the substrate is different?

Response: It is highly surprising because this is the first indication that the Mo, which is essential for the enzymatic activity of DMSO reductase family members might be dispensable for the sulfate reductase activity of the Xsr complexes. This concept is presented in Title and every section thereafter.

“We noticed that Salmonella utilizes” Overinterpretation, no argumentation. Expand.

Response: The revised text presents the information without interpretation, which now reads “Wild-type and Δ4 *Salmonella* grown in MOPS-GLC medium containing sulfite as sole sulfur source harbored similar intracellular concentrations of sulfite (**Fig 2d**).”

“Because H2S participates to” This statement is incorrect. Actually, FeS synthesis machineries (SUF and ISC) use cysteine as substrate. The ref 19 is outdated, several more recent reviews have been published since.

Response: The reviewer is correct that H_2S indirectly promotes [Fe-S] cluster biogenesis by serving as substrate for the synthesis of cysteine, which is the substrate of desulfurases such as SufS and IscS. The expanded text explains this pathway in more detailed. In addition, we have added a more recent review.

“NADH dehydrogenase” I think it contains 9 FeS, with 7 4Fe4S.

Response: Thanks for the clarification. This error has been fixed in the revised document.

Discussion: Talking about the GOE seems far from the results.

Response: The phylogenetic analysis indicates that DmsA and Xsr3 originated sometime during the GOE, whereas Xsr1 and Xsr2 may predate the biogeochemical GOE transition at the Archean-Proterozoic boundary. Therefore, we feel discussing the evolution of Xsr sulfate reductases relative to the GOE is justified.

Reviewer #3 (Remarks to the Author):

General Assessment

Kim et al. present an interesting and conceptually ambitious study demonstrating that Salmonella Typhimurium possesses a previously unappreciated metabolic flexibility in utilizing sulfide sources beyond cysteine and related metabolites. The authors identify three novel

periplasmic sulfate reductases that confer oxidative stress protection and provide a fitness advantage in the streptomycin-pretreated mouse model. Mechanistic analyses suggest these enzymes support sulfate assimilation through a catalytic mechanism independent of the canonical MOCO active site, and the authors further speculate on an evolutionary link to Earth's atmospheric history.

Response: Thank you.

The work is intriguing and impactful, but the narrative currently feels uneven. Foundational concepts are introduced too briskly, several experimental rationales are unclear, and some important biological implications remain underdeveloped. The manuscript appears structured around a four-figure format. Since Nature Microbiology provides substantially more space, I encourage the authors to expand the presentation and give their very interesting data the room it deserves.

Response: In response to the three Reviewers, we have now expanded the narrative. Hopefully, the added information makes the novelty of our findings more obvious.

Major Comments

1. Introduction lacks essential biological context

The integration of Earth's atmospheric history with Salmonella physiology is conceptually compelling; however, the introduction moves too quickly over foundational aspects of anaerobic respiration and sulfur assimilation. Before delving into mechanistic detail, the authors should expand on:

• The energetics of anaerobic terminal electron acceptors.
• The role of sulfur in bacterial metabolism.
• How sulfur assimilation pathways intersect with oxidative stress defences.

In addition, incorporating recent insights into Salmonella hydrogen sulfide production - particularly its roles in Fe–S cluster repair, intracellular redox balance, and stress tolerance - would strengthen the biological narrative and improve accessibility for a broad readership.

Response: We thank the reviewer for the suggestions, which have been incorporated into the revised Introduction. Some of these ideas have also been incorporated into the Discussion.

Figure 1

2. Link between oxidative stress findings and later sections is missing

The first results paragraph demonstrates that periplasmic sulfate reductases protect against oxidative stress and provide a fitness advantage in the streptomycin-pretreated mouse model. However, these findings are not revisited in the Discussion. The authors should address:

• Whether sulfur availability is limiting in the gut.
• Whether Salmonella kept these pathways to exploit alternative sulfur sources.
• The mechanistic link between sulfur metabolism and oxidative stress tolerance.

Response: The Discussion has been revised to incorporate these concepts.

3. Clarify whether p.o. infections used streptomycin-treated mice

In the Fig. 1 infection experiments, it is not clear whether C57BL/6 mice were microbiota-perturbed with streptomycin in the main text. This should be explicitly stated.

Response: As indicated in the M&M and **Fig 1c**, some of the p.o. infections were conducted in streptomycin-treated mice. In response to Reviewer #3, we have conducted some of the p.o. infection in mice that were not pre-treated with streptomycin. Please, see the revised **Fig 1c**.

4. Consider testing the Δ4 phenotype in additional models

As most fitness assays rely on streptomycin-pretreated mice, it would be valuable to assess the

phenotype in a model that does not depend on antibiotic perturbation. Antibiotic treatment induces oxidative stress and alters luminal metabolism, potentially affecting sulfate availability.

Response: As suggested by the reviewer and discussed above, we have now tested the Δ*4* strain in an oral model of infection without antibiotic pre-treatment. The results, which are shown in **Fig 1b**, show that the Δ*4* strain is attenuated when inoculated into C57BL/6 mice with an intact microbiota at the time of infection. This information, which has been added to the revised Results section, suggest that the extracytoplasmic sulfate reductases help *Salmonella* overcome colonization resistance without antibiotic perturbation.

5. Fig. 1d
What does a competitive index (CI) of ~0 indicate? Does it reflect a detection-limit issue, or is the x-axis mislabeled?

Response: The data are represented in linear scale. The lowest value is 0.0543.

Figure 3
6. Speculation on possible undiscovered MOCO-associated activity of Psr3
The manuscript briefly hints that Psr3 may have additional, uncharacterized activity. The authors are encouraged to articulate what functions they consider plausible.

Response: This criticism was raised by Reviewer #2 as well. As of now, we don't have any evidence to demonstrate that the MOCO cofactor is functional in Xsr enzymatic complexes. Therefore, this statement has been erased from the revised document. The loading of MOCO into the extracytoplasmic sulfate reductases will require significant experimentation, which is beyond the goals of the current work.

7. Were Psr1 and Psr2 tested in a moa-deficient background?
It is unclear why the analysis focuses predominantly on Psr3. The authors should clarify whether:
• Psr1 and Psr2 were also examined in a MOCO-deficient background, and the emphasis on Psr3 is due to motif similarity with DmsA or another specific rationale?

Response: As suspected by the Reviewer, we mostly focused on Xsr3 because of its closest relationship with DmsA. The phenotypes of strains bearing a mutation in the conserved serine residue known to coordinate the molybdenum cofactor in other DMSO reductase family members suggest that the Xsr1 and Xsr2 paralogs do not use the MOCO cofactor in their catalytic reduction of sulfate (see **Fig S5b**). In response to the Reviewer's comment, we have now tested the sulfate reductase activity of *xsr1*$^{+}$ Δ4 Δ*moa* and *xsr2*$^{+}$ Δ*4* Δ*moa* strains (see **Fig 4c**). The Δ*moa* mutant allele does not preclude sulfate reductase activity in any of the Xsr complexes. Together, our experiments demonstrate that Xsr enzymatic complexes perform sulfate reduction in the absence of the MOCO cofactor.

Figure 4
8. PhsABC should be explicitly mentioned
In the text corresponding to Fig. 4f, it is unclear why PhsABC is not named in the main text.

Response: The Δ*phsABC* mutant was used as a negative control, as in the absence of thiosulfate (i.e., MOPS-GLC minimum medium containing SO_4^{2-} as sole sulfur source), this MopB family member should not be a source of H_2S. As requested by the Reviewer, PhsABC is now mentioned in the text.

Discussion:
The authors frame sulfur assimilation in the context of Earth's atmospheric history, yet Salmonella is fundamentally a gut-associated pathogen causing enteric disease. It would be

valuable for the Discussion to address this contrast more directly - specifically, how these pathways operate and evolve within the ecological and metabolic constraints of the gut environment. And how additional sulfur assimilation pathways or oxidative stress–protection mechanisms might further promote Salmonella establishment and persistence in the gut.

Response: The evolution of *xsr* family members in the gut is probably best framed in the context of typhoidal and non-typhoidal serovars, with the former having acquired inactivating mutations in all *dmsA* paralogs. The negative selection of *dmsA* paralogs in Typhi and Paratyphi could be driven by the fact that these human-adapted serovars dampen the respiratory burst of phagocytic cells and are auxotrophs for cysteine, making H_2S a less pressing commodity. These concepts have now been discussed in the revised manuscript.

Minor Comments

9. Rationale for gene prioritization needs clarification

The manuscript uses psr1, psr2, psr3, and STM14_1811 in different contexts without clearly explaining the reasoning. For example:

• Why is psr3 prioritized over psr2 in Fig. 3?

Response: As discussed above, Xsr3 was prioritized over the other two extracytoplasmic sulfate reductases in the EPR studies because of its closest relatedness to DmsA. This information has been added to the revised document. Having said that, we have conducted extensive genetic, biochemical and functional characterization of *xsr1* and *xsr2*, and have added new evidence that shows that Xsr1 and Xsr2 complexes support sulfate reductase activity in a Δ*moa* background.

• Why is STM14_1811 introduced in Fig. 1 but not referenced again until Fig. 4?

A brief rationale at the start of each relevant section would make the work significantly easier to follow.

Response: As discussed above in relation to one of the questions raised by Reviewer #2, we now know that STM14_1811 encodes for a selenate reductase. We hope to submit a paper on the detailed characterization of STM14_1811 soon. As suggested by the Reviewer, we have added a brief rationale at the beginning of each section.

10. Integrate findings with recent work on sulfide-mediated competition

A recent PNAS study (“Salmonella produces sulfide to compete with Escherichia coli in the gut lumen”) shows that hydrogen sulfide produced by PhsABC and AsrABC suppresses commensal E. coli, giving Salmonella a competitive edge. The authors should discuss:

• How the newly described pathways differ from PhsABC/AsrABC-mediated sulfide production,

• How these findings inform the ecological significance of sulfur metabolism in the gut.

A concise paragraph in the Discussion would suffice.

Response: Thank you for pointing out this important paper. We find it remarkable that the activity of several of the MopB family members (DmsABC, PhsABC, ArsABC, Xsr1ABC, Xsr2ABC, and Xsr3ABC) directly or indirectly promote sulfide synthesis. The paper referred by the Reviewer has now been presented into the revised Introduction and Discussion sections.

Reviewer #1 (Remarks to the Author):

The manuscript reads much better and addresses most if not all the reviewers' comments. A few minor corrections:
Thank you very much.

line 88: spell out "i.p." first time
Response: Done

line 117: replace "levels" with "cell densities"
Response: Done

line 139: what "reductase"? (grown Salmonella reduced reductase,)
Response: Sorry for the typo. It was meant to say “sulfate.” The error has been corrected.

line 169: add "periplasmic" (periplasmic nitrate reductase)
Response: Done

line 423: conditions
Response: The typo has been corrected

line 517: need reference for extinction coefficient as it changes at different wavelengths
Response: A reference has been included.

reference section has italics missing for genus and species names
Response: Genus and species have now been italicized.

figure 2: percentages should be centered
Response: Percentages have been centered

figure 4: line 935 - evaluated for DMSO
Response: The sentence has been corrected.

Reviewer #2 (Remarks to the Author):

The manuscript by Kim et al. has undergone significant improvement and the scope of the analysis remains impressive. However, the text still does not fit with the standards expected for Nature Microbiology and would benefit from a thorough revision, including multiple corrections for clarity and precision. I still have doubts on phylogenetic analysis that require attention.
Response: We thank Reviewer 2 for the continued careful reading and for acknowledging the significant improvements made in the revised manuscript. We have addressed each of the remaining concerns below and hope that the revised manuscript now meets the standards expected for *Nature Microbiology* with respect to both writing quality and phylogenetic rigor.

Major comments
The scientific narrative remains unclear in several sections, with missing conclusions and overly concise sentences that fail to adequately reflect the extensive work conducted. This is unfortunate, as it undermines the impact of the substantial efforts invested. While I have highlighted some specific points below, I strongly recommend a comprehensive rephrasing of

the entire manuscript, possibly with the assistance of colleagues or language models, to enhance readability and coherence.

Response: We have undertaken a substantial revision of the manuscript with particular attention to the sections identified as problematic. We believe the revisions substantially improve the readability and scientific coherence of the phylogenetic sections and better reflect the scope and rigor of this component of the work.

I am still not convinced by the phylogenetic analysis, particularly regarding the root used in Figure 6. The rationale for selecting the two archaeal sequences as an outgroup is not provided, yet this is a critical aspect that requires justification. The outgroup must be at least as ancient as the group of interest, otherwise, the quality of the interpretation could be severely compromised if the root is incorrect.

Response: The rationale for outgroup selection has now been fully addressed. The sequences used to root the focused DmsA_EC tree belong to the MopB_Arsenate-R family (CD02757) that was identified as the immediate sister group to the DmsA_EC clade in our global MopB phylogeny of 3,324 sequences (**Extended Data Figure 8**). This approach is consistent with prior comprehensive MopB superfamily phylogenies (Wells *et al*. 2023), which consistently recovered DmsA_EC and MopB_Arsernate-R families as closely related sister lineages. Using the immediate sister MopB_Arsenate-R clade as outgroup follows standard phylogenetic practice for rooting focused clade analyses (PMCID: PMC7149615; PMID: 40839422; 37172862; 30364468; 23736103; 12805537; 19240196; 12270903; 34892859). In the original submission, archaeal Thermoproteota representatives from the Arsenate-R family were selected as outgroup on the premise that deep-branching archaeal members of ancient MopB families tend to occupy positions consistent with early divergence, providing a conservative root. However, following current best practice for phylogenetic rooting, which recommends selecting the most closely related relative to minimize random rooting artifacts (DeSalle et al. 2023, PMID: 37172862), we have revised the analysis to use bacterial Arsenate-R sequences as the outgroup. The new rooting strategy using bacterial Arsenate-R as outgroup yielded an identical tree topology to that reported in our previous version of the manuscript. The consistency of tree topology across both outgroup choices confirms that root placement is not sensitive to outgroup selection within the Arsenate-R sister family, demonstrating the robustness of our rooting strategy.

Additionally, the authors mention introducing taxonomic distribution, but I did not find a proper distribution in sup. It is essential to provide a complete list of the studied genomes, including those in which no homologs were identified.

Response: Our phylogenetic dataset was derived from UniRef90, a curated non-redundant protein sequence database, rather than from assembled genomes. Because we used JackHMMER to search protein sequences, rather than mining genome assemblies, conventional genome quality metrics such as completeness and contamination percentages, typically assessed using tools like CheckM, do not apply to our workflow. Our thorough filtering methodology ensured that only high-confidence, functionally coherent DmsA-like sequences were retained for phylogenetic analysis, providing a quality-controlled dataset equivalent in rigor to a genome completeness-filtered assembly-based approach. Our strategy follows a similar approach used in published work (PMID: 26454276; 20718988; 22039361; 25398609; 31777944; 33125078; 29035372; 29425356; 23060610). To address the Reviewer's concern regarding taxonomic distribution and completeness of metadata for all recovered sequences, Supplementary Data File 1 contains the full taxonomic lineages, protein name, protein length, and UniProt accessions. To make the taxonomic scope of our analysis immediately accessible, we have also added a new supplementary figure (**Extended Data Figure 10**) showing the phylum-level representation of DmsA_EC homologs at each sequential filtering step, from the initial 246,760 JackHMMER hits through the final curated dataset. This figure demonstrates that

taxonomic breadth was maintained throughout the curation workflow and that our filtering steps did not introduce systematic taxonomic bias.

Also, the last part of the results section is overly wordy and could be tightened, with some parts that read more like filler than analysis.
Response: The last section of the Results has been substantially revised to remove descriptive text and interpretive claims. We have also tightened the narrative to focus exclusively on directly supported analytical conclusions.

Specific comments
L53: Why use the term "founding"?
Response: The term “founding” has been erased from the revised document.

L54: Remove "significant".
Response: The term “significant” has been erased from the revised document.

L56: The term "revolution" is exaggerated. Consider a more precise term.
Response: The term “revolution” has been replaced with the term “diversification.”

L76: Replace "reductase" with "reductase activity".
Response: Done.

L76–78: There is no causal link between distinct gene clusters… and duplication/HGT events...
Response: As requested, we have added a clause with the conclusion that can be drawn from the findings.

L89: A conclusion is missing here.
Response: As requested, we have added a clause with the conclusion that can be drawn from the findings.

L96: A conclusion is needed.
Response: As requested, we have added a clause with the conclusion that can be drawn from the findings.

L107–111: Justify why 1811 will no longer be studied. This seems very odd for a reader that does not know that the authors plan to study it in more detail.
Response: We have now included the assessment of sulfate reductase activity in membranes isolated from a Δ*4* strain that was complemented with the 1811 gene. The results, which are presented in **Extended Data Figure 4a,** show the lack of sulfate reductase activity by the *STM14_1811*-encoded DmsA paralog. The added data provide the rationale for no longer studying the STM14_1811 gene product in this paper.

L113–119: This section is confusing. Rephrase and reorganize for clarity.
Response: The text has been edited for clarity.

L132–133: The conclusion is unclear. It would be more accurate to state that DsmA paralogs do not utilize MoCo.
Response: The experiments presented originally in L132-133 show that the Δ*4* strain respires on thiosulfate and dimethyl sulfoxide, which are substrates utilized by DMSO reductase family members encoded in *phsABC* and *dmsABC* gene clusters. These data demonstrate that other

members of the DMSO reductase superfamily remain functional in the *xsr* mutant strains. The text has been modified to clarify this conclusion.

L137–138: Why would it be surprising that CysIJ is expressed normally under these conditions? Clarify.
Response: We don't think this finding is surprising. We examined cytoplasmic extracts to determine if the canonical sulfate reductase is still present in the Δ*4* strain.

L147: As previously observed?
Response: The sentence has been edited as suggested by the Reviewer.

L164–165: And other quinones?
Response: *Salmonella* produces ubiquinone, menaquinone and demethylmenaquinone. We did not test demethylmenaquinone. Therefore, the conclusions in this passage are limited to ubiquinone and menaquinone.

L184–185: These results need further development.
Response: As requested, the genetic set up is more clearly explained in the revised document.

L189–190: This idea should precede the previous sentence for logical flow.
Response: As suggested, the sentence in question has been position immediately after the description of DMSO reductase activity.

L190–192: This sentence condenses multiple experiments and should be expanded.
Response: As suggested, the data are now discussed in two separate sentences.

L194–195: There is no causal link between "given..." and "we examined...".
Response: The sentence has been edited.

L196–198: Rephrase for better readability.
Response: As suggested, the sentence has been edited.

L198–200: There is no causal link between "All A, B..." and "suggesting that...".
Response: The sentence has been edited for clarity.

L202: Use "determine" instead of "visualize" and "performed" instead of "pursued".
Response: Done.

L206: The term "complemented" seems inappropriate here, as you are not restoring a phenotype.
Response: The text has been modified.

L208–212: Expand on these results for better understanding.
Response: As requested, we have expanded the text.

L221: How can a reduction occur IN a [Fe-S] cluster? Consider rephrasing to: "[Fe-S] cluster is directly involved in sulfate reduction."
Response: As suggested, the change has been made.

L231: "Was more toxic" is a conclusion, not an observation. Rephrase to reflect the data.
Response: The sentence has been modified.

L255: How was it determined that NADH dehydrogenase activity is solely due to Nuo? Be more precise and develop.
Response: The *nuo*-encoded, not the alternative *ndh*-encoded NADH dehydrogenase, utilizes deamino-NADH. The results section has been modified to clarify that the enzymatic activity of the *nuo*-encoded NADH dehydrogenase was assessed by following the oxidation of diamino-NADH. This information was originally presented in Materials and Methods.

L262: The title is unclear. What is meant by "discrete evolutionarily ordered radiation"? This terminology is ambiguous and potentially overinterpreted.
Response: The section title has been revised to read: "DmsA paralogs represent a widespread bacterial MopB lineage with diverse iron-sulfur cluster coordination".

L263: How can a phylogeny "identify" something?
Response: This sentence has been removed from the revised document.

L270: Define "MopB_DmsA-EC".
Response: MopB_DmsA_EC stands for DmsA-like extracytoplasmic subgroup of the MopB superfamily, as classified by the NCBI Conserved Domain Database (CDD accession CD02770). This information has been provided at first mention in the text.

L271–273: The claim that these niches are enriched requires justification. Was an ecological niche analysis performed? If not, clarify or remove this statement.
Response: The statement has been revised to reflect the isolation sources of the organisms represented in the dataset, rather than implying a formal ecological niche analysis. The revised text reads: "DmsA_EC homologs are broadly distributed across diverse bacterial and archaeal phyla, encompassing sequences from environmental, sedimental, and host-associated organisms. These findings indicate that the DmsA-like architecture is not restricted to Proteobacteria (**Extended Data Figure 9**, **Extended Data Figure 10**)."

L274: Use "used to infer" instead of "subjected."
Response: The change has been made.

L286–298: This section is overly verbose and lacks focus. Consider revising for conciseness.
Response: The revised text substantially condenses the description of the phylogenetic distribution of DmsA_EC homologs within Gammaproteobacteria, retaining only the key topological findings directly relevant to the *Salmonella* paralogs.

L299: Replace "RelTime analysis suggests" with "Dating analysis suggests."
Response: Done.

L305: What is meant by "the crown"? This terminology is unclear and should be defined.
Response: The term ‘crown’ has been removed.

L311–317: This paragraph could be more impactful if compared to findings in Xsr3, particularly to assess the phylogenetic distribution of this type of Fe-S cluster.
Response: The analysis in **Fig 6b** shows that any given FS0 architecture are present in phylogenetically distant bacteria across different branches of the ML tree. For example, DmsA and Xsr3A share identical $CX_3CX_3CX_{20\text{-}40}C$ spacing, but exhibit distinct substrate preference.

Therefore, FS0 per se might not be solely responsible for substrate specificity. Consequently, the passage mentioned by the Reviewer has been erased from the revised document.

Finally, the images in the figures are pixelated and unreadable. Higher-resolution images are necessary for clarity.
Response: The images were generated with Prism or Illustrator and saved at 300 dpi. They have now been exported as PDF files, and we anticipate that the vector format will provide improved resolution.

Reviewer #3 (Remarks to the Author):

The authors have thoroughly revised the manuscript and addressed all comments. I would like to congratulate them on this very interesting study.
Thank you for your support.

The authors followed most of my recommendations, and I am satisfied with the last version of the manuscript.
Thank you very much for your support.

A few minor comments below:

L28: “Diverse” instead of “Disparate”. Response: done.
L75: Genes have no activity. Replace by protein. Response: done.
L92-93: Develop this sentence. A “construction” can not “reveal”. Response: the word construction has been erased from the revised sentence.
L153: “CysIJ” after “cytoplasmic sulfate reductase”’. Response: done.
L201: The delta5 strain has not been defined before. Response: the strain is now described.
L222: Please replace FS0 by [Fe-S] cluster everywhere. This would be more explicit for most of readers. Response: done.
L225: Why 5 DmsA paralogs? There are only 4. Do the authors mean DmsA and its 4 paralogs? Is it a different mutant from L201? Response: as suspected by the reviewer, this strain is lacking *dmsA* and its four paralogs. This information has been clarified in the revised document.
L226-228: Simplify these sentences. Response: we believe these sentences are clear and appropriate as written.
L313: “Distinct” instead of “Discrete”. Response: done.
L317 “clade” instead of “grade”. Response: done.
L320 “grouped” instead of “ recovered”. Response: done.
